# Design, Synthesis, and Antibacterial and Antifungal Activities of Novel Trifluoromethyl and Trifluoromethoxy Substituted Chalcone Derivatives

**DOI:** 10.3390/ph13110375

**Published:** 2020-11-09

**Authors:** Surendra Babu Lagu, Rajendra Prasad Yejella, Richie R. Bhandare, Afzal B. Shaik

**Affiliations:** 1 Department of Pharmaceutical Sciences, Pharmaceutical Chemistry Division, A.U. College of Pharmaceutical Sciences, Andhra University, Visakhapatnam 530003, Andhra Pradesh, India; dryrp_au@rediffmail.com; 2 Department of Pharmaceutical Sciences, College of Pharmacy & Health Sciences, Ajman University, Ajman P.O. Box 346, UAE; 3 Department of Pharmaceutical Chemistry, Vignan Pharmacy College, Vadlamudi 522213, Andhra Pradesh, India

**Keywords:** fluorinated compounds, chalcones, trifluoromethyl, trifluoromethoxy, antibacterial activity, antifungal activity, minimum inhibitory concentration, cytotoxicity

## Abstract

Despite the availability of many drugs to treat infectious diseases, the problems like narrow antimicrobial spectrum, drug resistance, hypersensitivities and systemic toxicities are hampering their clinical utility. Based on the above facts, in the present study, we designed, synthesized and evaluated the antibacterial and antifungal activity of novel fluorinated compounds comprising of chalcones bearing trifluoromethyl (**A1**–**A10**) and trifluoromethoxy (**B1**–**B10**) substituents. The compounds were characterized by spectroscopic techniques and evaluated for their antimicrobial activity against four pathogenic Gram-positive (*Staphylococcus aureus* and *Bacillus subtilis*) and Gram-negative (*Escherichia coli* and *Bacillus subtilis*) bacterial and fungal (*Candida albicans* and *Aspergillus niger*) strains. In this study, the compounds with trifluoromethoxy group were more effective than those with trifluoromethyl group. Among the 20 fluorinated chalcones, compound **A3**/**B3** bearing an indole ring attached to the olefinic carbon have been proved to possess the most antimicrobial activity compared to the standard drugs without showing cytotoxicity on human normal liver cell line (L02). Further, the minimum inhibitory concentration (MIC) for **A3**/**B3** was determined by serial tube dilution method and showed potential activity. These results would provide promising access to future study about the development of novel agents against bacterial and fungal infections.

## 1. Introduction

Infectious diseases in human beings are caused by microbes including bacteria, fungi, and viruses. These diseases are treated by employing a range of antimicrobials available in the market. The utility of antimicrobials in therapy is ever-increasing, which is leading to the most dangerous problem, antimicrobial resistance (AMR) [1,2]. Resistance to antimicrobial agents is a major threat to public health and is responsible for significant rise in morbidity, mortality, and hospitalization. Keeping this in view, World Health Organization (WHO) introduced a preamble “*no action today no cure tomorrow*” to counteract the trouble of AMR [3,4,5]. The limitations of current antimicrobial agents like AMR, untoward side-effects, lengthier treatment period, and improper therapeutic outcomes compel the development of more effective novel antimicrobial chemical entities that can be employed as drugs. Scientists are in a continuous search for novel antimicrobial agents employing different strategies. One common strategy followed by researchers is the design and synthesis of small molecules for testing them as prospective antimicrobial drug candidates. Small molecule drugs play a significant role in treating different types of diseases. In recent years, according to United States-Food and Drug Administration (US-FDA), the development of small molecules has reduced to some extent (74% in 2017 and 71% in 2018). However, in 2019, nearly 70% of the total of approved targeted drugs were small molecules [6,7]. Advantages of small molecules include better pharmacokinetic properties, oral bioavailability, delivery, and production cost [8]. Small molecules bring chemical diversity in less time and with ease compared to isolation, structural elucidation and biological testing of natural products. Additionally, screening of small molecule libraries derived through laboratory synthesis is widely employed in the pharmaceutical industry to identify lead molecules with potential drug-like properties [9].

**Figure 1:**
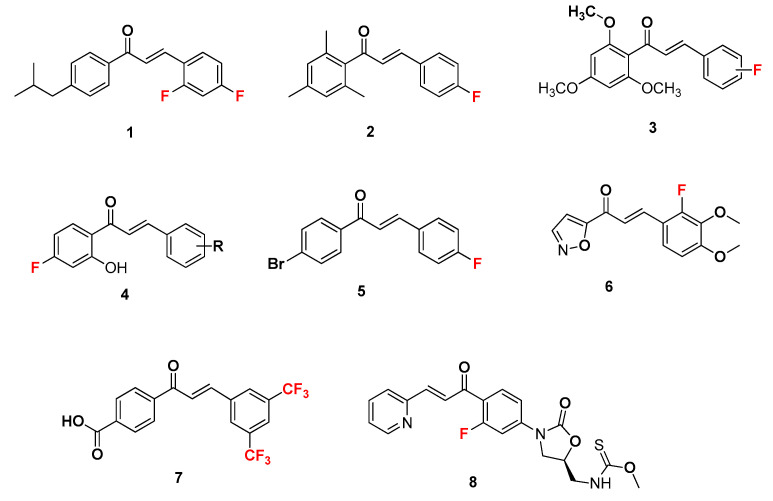
Structures of fluorinated chalcones with antibacterial and antifungal activities.

Chalcones are bichromophoric natural open-chain flavonoid small molecules containing a reactive propenone linkage connected to two aromatic rings. The propenone moiety of these molecules is highly reactive and is not only responsible for the assorted biological activities of chalcone derivatives [10] but also for the preparation of different heterocyclic derivatives from the chalcones. Chalcone is a privileged scaffold of interest to organic and medicinal chemists because of its ease of synthesis and multiple biological activities. Chulin et al. have published a critical and comprehensive review on this privileged scaffold [11]. Chalcone derivatives containing fluorine and or other types of substituents were reported to possess excellent anticancer, antimicrobial, antioxidant, anti-inflammatory, analgesic, cancer chemopreventive, antibacterial, and antifungal activities [12,13,14,15,16,17,18,19,20,21]. Xu et al. have reviewed the structural features of different chalcone derivatives and their influence on the antibacterial properties [16]. There is a difference in the biological potency of different chalcones, which is majorly attributed to the type of aromatic ring as well as the nature of the substituents present thereof. Literature survey and results from our own studies proved that fluorinated chalcones possess remarkable antibacterial and antifungal properties (Figure 1) [22,23,24,25,26,27,28,29,30]. The potent antimicrobial properties of the chalcones may be due to extra lipophilicity created by the fluorine atoms. With few exceptions, most of the antimicrobial fluorinated chalcones reported typically possess one or more fluorine atoms on the two aryl rings of the chalcone scaffold but not either a trifluoromethyl or trifluoromethoxy group. For instance, out of the eight compounds displayed in Figure 1, only compound **7** possesses a trifluoromethyl group. Hence, in the present study we considered synthesizing and evaluating novel chalcones with trifluoromethyl and trifluoromethoxy groups as prospective antibacterial and antifungal agents.

**Figure 2:**
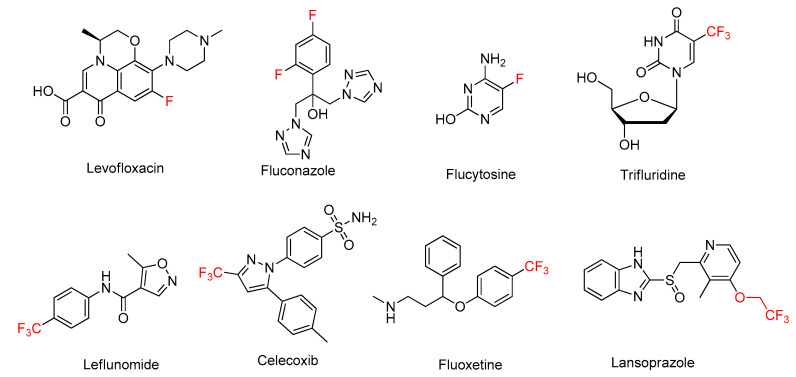
Structures of selected drugs containing one or more fluorine atoms.

Presence of one or more fluorine atoms can be seen in a range of drugs used for different disorders including the antibacterials: fluoroquinolones; antifungal: fluconazole; antivirals: efavirenz, trifluridine; anticancer agents: 5-fluorouracil, bicalutamide, leflunomide; antifungals: 5-flucytosine; antidepressants: fluoxetine, escitalopram; steroids: dexamethasone, triamcinolone, fludrocortisone; selective COX-II inhibitors: celecoxib; antiulcer: lansoprazole; antihyperlipidemic agents: atorvastatin, rosuvastatin, ezetimibe, and an antischizophrenic agent: risperidone (Figure 2). The presence of fluorine atoms in the medicinally active compounds have imparted some special properties, including increased binding interactions, potency, permeability, metabolic stability, decreased *pka*, clearance, alteration of the conformation, modified physical properties, and selective reactivities [31,32,33,34,35].

**Figure 3:**
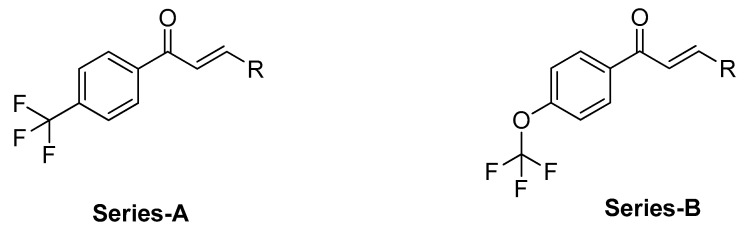
General structures of the designed target fluorinated chalcones.

Motivated by the aforementioned facts, herein we have designed and prepared two series of novel chalcones substituted with trifluoromethyl (series-A) and trifluoromethoxy (series-B) groups (Figure 3) and evaluated further for their antibacterial and antifungal activities against selected clinically significant bacterial and fungal strains.

## 2. Results and Discussion

### 2.1. Chemistry

The two series of chalcones were afforded by the Claisen–Schmidt condensation reaction of substituted aryl and unsubstituted heteroaryl aldehydes with two different types of ketones, i.e., 4′-trifluoromethyl acetophenone (series-A) and 4′-trifluoromethoxy acetophenone (series-B). The reaction time utilized for the formation of series-A chalcones was around 6–12 h, whereas for series-B chalcones, it was 12–15 h, and the yield of series-A chalcones was more than the latter. This may be due to the high electron-withdrawing nature of the trifluoromethyl substituent over the trifluoromethoxy group. The compounds were in yellow to orange color which may be due to the extensive conjugation of chalcone core and the additional electronic effects of the substituents on the ring-A and B.

All the compounds were characterized by elemental analysis, FT-IR and ^1^H NMR, whereas the compounds **A3** and **B3**, were additionally characterized by ^13^C NMR and mass spectral methods. The elemental analysis and spectroscopic data were consistent with the expected structures of the chalcones. All the compounds in their FT-IR spectrum showed two characteristic stretching absorption bands corresponding to the propenone linkage including C=O and C=C around the wave numbers 1656–1695 cm^−1^ and 1502–1514 cm^−1^ for the ten compounds in series-A and 1640–1656 cm^−1^ and 1502–1532 cm^−1^ for series-B compounds, respectively. The ^1^H NMR spectra showed two diagnostic doublet signals corresponding to *α*- and *β*-protons resonating between the chemical shift values of 7.32–7.75 ppm and 7.76–8.04 ppm (series-A) and 7.51–7.65 ppm and 7.60–8.18 ppm (series-B). The coupling constant value, *J*, for these doublets ranged between 15 to 17 Hz, and such large coupling constant values indicate that the synthesized compounds have *trans* geometry at the olefinic bond of the propenone linkage.

The FT-IR spectrum of **A3** illustrated intense carbonyl band (C=O) of chalcones at 1695 cm^−1^ and strong stretching band at 1573 cm^−1^ accounting for vinyl (CH=CH) double bond. In addition, the other absorption bands were seen at wave numbers 3230 cm^−1^ (-NH in indole) and 1263 cm^−1^ (-CF_3_). Two doublet peaks at 7.77 ppm and 8.13 ppm with the coupling constant value around 16 Hz in the ^1^H NMR spectrum confirmed the formation of chalcone bridge of **A3** with trans geometry. The indole -NH proton showed a peak at 8.78 and the other nine aromatic protons showed two multiplets around 7.94–8.14 and 7.05–7.61, respectively. The ^13^C NMR spectra of compound **A3** demonstrated three peaks at 189.93, 128.60, and 142.02 corresponding to the three carbons of the propenone moiety. The other ^13^C peaks include 140.11 (C-1′), 128.60 (C-2′ and 6′), 125.59 (C-3′ and 5′), 137.30 (C-4′), 123.77 (C-4,′), 129.52 (C-6′), 112.06 (C-1″), 125.30 (C-2″), 130.30 (C-4″), 114.06 (C-5″), 120.71 (C-6″), 122.02 (C-7″), 117.44 (C-8″), 125.56 (C-9″). The mass spectrum of **A3** recorded in positive mode has given an [M + H]^+^ peak at m/z 316.30. In the ^19^F-NMR spectrum, **A3** showed signal at 63.27 ppm corresponding to -CF_3._ The above spectral data confirmed the compound **A3** as (*E*)-3-(1″H-indol-3″-yl)-1-[4′-(trifluoromethyl)phenyl]prop-2-en-1-one.

**Figure 4:**
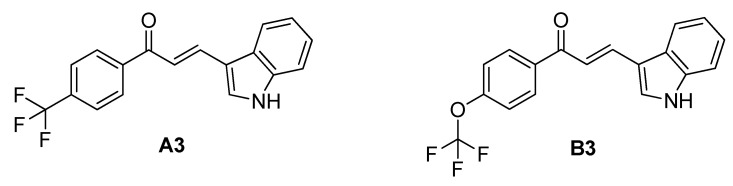
Structures of fluorinated chalcones **A3** and **B3.**

The FT-IR spectrum of **B3** illustrated diagnostic intense carbonyl (C=O) and strong vinyl (CH=CH) absorption bands of chalcone linkage at wave numbers 1651 cm^−1^ and 1595 cm^−1^, respectively. Additionally, the other absorption bands are seen at 3681 (-NH in indole), 1206 (-OCF_3_), and 1248 (C-O-C). The ^1^H NMR spectrum showed characteristic doublet signals at 7.53 ppm and 8.05 ppm with the coupling constant value (*J*) around 16 Hz. The larger coupling constant value represents the trans geometry of the chalcones. The other peaks seen are three singlets corresponding to the indole amine proton at chemical shift 8.78 and two multiplets around 7.38–8.02 and 7.05–7.66 corresponding to nine aromatic protons. The ^13^C NMR spectra of compound **B3** showed three peaks corresponding to the three carbons of the propenone moiety at 189.97, 125.33, and 137.37, respectively. The peaks of other carbon atoms are seen at the following chemical shift values: 130.23 (C-1′), 130.71 (C-2′ and 6′), 121.88 (C-3′ and 5′), 139.61 (C-4′), 121.88 (C-4,′), 112.08 (C-1″), 123.63 (C-2″), 137.35 (C-4″), 114.41 (C-5″), 120.47 (C-6″), 122.69 (C-7″), 117.81 (C-8″), 123.63 (C-9″). The mass spectrum of **B3** recorded in positive mode showed [M + H]^+^ peak at m/z 332.20. In the ^19^F-NMR spectrum, **A3** showed signal at 63.27 ppm corresponding to -CF_3._ The ^19^F-NMR spectrum of **B3** showed signal at 58.64 ppm corresponding to -OCF_3._ Based on the above spectral data, the compound **B3** was confirmed as (*E*)-3-(1″H-indol-3″-yl)-1-[4′-(trifluoromethoxy)phenyl]prop-2-en-1-one (Figure 4).

### 2.2. Biological Activities

#### 2.2.1. Antibacterial and Antifungal Activities

All the 20 target compounds (**A1**–**A10** and **B1**–**B10**) were evaluated for their antibacterial and antifungal activities on selected pathogenic Gram-positive (*Staphylococcus aureus* and *Bacillus subtilis*) and Gram-negative (*Escherichia coli* and *Proteus vulgaris*) bacterial (Table 1) and fungal (*Candida albicans* and *Aspergillus niger*) strains (Table 2). The compounds were tested at 0.1% concentration. Benzyl penicillin was employed as standard drug against the bacterial isolates and fluconazole against the fungal strains. The compounds were partitioned into three different categories based on the type of ring-B portion of chalcones. Among the 20 target derivatives, **A2(B2)**, **A4(B4)**, **A5(B5)**, **A8(B8)**, and **A9(B9)** represents chalcones bearing a monosubstituted aryl ring, **A1(B1)** denotes compounds with a disubstituted aryl ring, and **A3(B3)**, **A6(B6)**, **A7(B7)**, and **A10(B10)** designate the compounds containing an unsubstituted heteroaromatic ring.

The compounds exhibited varying degrees of activity (Table 1 and Table 2), i.e., some compounds were more active than the standard drugs, whereas some were moderately active and others were less active. When the results between the two series of compounds were compared, a greater number of the compounds belonging to series-B comprising of the -OCF_3_ group exhibited more activity than the series-A compounds bearing -CF_3_. However, against *Candida albicans*, five compounds belonging to series-A showed superior activity than series-B compounds. Out of the six microbial strains used in our study, the Gram-negative bacteria *Escherichia coli* and *Proteus vulgaris* were more vulnerable to both the series of chalcones than the Gram-positive bacterial species and fungal isolates. With few exceptions, the activity against Gram-positive bacterial and fungal strains is either intermediate or less.

Among all the compounds, **A3** and **B3** containing an unsubstituted heteroaromatic 3-indolyl moiety showed more activity than the standard drugs against the tested bacterial and fungal species. This illustrates that the presence of a bicyclic heteroaromatic scaffold is a major contributing factor for the activity of chalcones bearing -CF_3_ and -OCF_3_ groups. Against *Staphylococcus aureus*, **A3** and **B3** showed a zone of inhibition (ZOI) of 25 and 26 mm, respectively. The activity of **B3** (ZOI = 29 mm) was more than benzyl penicillin (ZOI = 27 mm) against *Bacillus subtilis* but the activity of **A3** (ZOI = 26 mm) was less than the standard. **A3** and **B3** also exhibited more activity than benzyl penicillin against *Escherichia coli* (ZOI = 20 and 22 mm) and *Proteus vulgaris* with a ZOI of 23 and 21 mm, respectively. **A3** and **B3** showed a ZOI of 20 and 22 mm against *Candida albicans* and a ZOI of 25 and 26 mm, respectively, against *Aspergillus niger*. These ZOI values were more compared to the ZOI values obtained with standard fluconazole (19.05 and 24.41 mm).

Except **B3**, all the other compounds displayed intermediate-to-low activity against *Staphylococcus aureus*, *Bacillus subtilis*, *Candida albicans* and *Aspergillus niger* compared to the standard drugs. When the activities of other compounds against *Escherichia coli* were compared with the standard drugs, the compounds **A1(B1)**, **A4(B4)**, **A5(B5)**, **A6(B6)**, **A7(B7)**, **A8,** and **A9** were found to be more active. Further, among the above 12 compounds, **A1** and **A7** containing 2,3-dichlorophenyl and 2-furfuryl rings were next in potency to **A3** in series-A and **B1**, **B4**, and **B5** containing 2,3-dichlorophenyl, 2-nitrophenyl, and 3-nitrophenyl scaffolds after **B3** in series-B. The chalcones **A1(B1)**, **B2**, **A4(B4)**, and **A5(B5)** were more active than benzyl penicillin against *Proteus vulgaris*, and the compounds **A1** (2,3-dichlorophenyl), **A4** (2-nitrophenyl), **A5** (3-nitrophenyl), **B2** (3-chlorophenyl), **B4** (2-nitrophenyl), and **B5** (3-nitrophenyl) showed equal activity with a ZOI value of 20 mm, whereas the compound **B1** containing 2,3-dichlrophenyl moiety showed activity similar to **B3** (ZOI = 21 mm). These results suggest that presence of an electron-withdrawing group like chlorine or nitro at 2nd and 3rd positions of aryl ring are essential for the activity. The compounds **A2** and **A7** bearing 3-chlorophenyl and 2-furfuryl rings showed equal activity as benzyl penicillin (ZOI = 19 mm) against *Proteus vulgaris*, and **B1** exhibited equal potency as fluconazole against *Candida albicans* with a ZOI of 19 mm. All the other compounds including **A8(B8)** and **A9(B9)** containing 4-chlorophenyl and 4-nitrophenyl rings as well as **A6(B6)** and **A10(B10)** containing heteroaromatic 2-thienyl and 2-pyrrolyl rings, respectively, showed intermediate-to-poor activity relative to the standard drugs. Based on these results, we can summarize that either in series-A or B, the conditions of the ring B-portion of chalcones being either an aromatic ring with electron-withdrawing groups in the ortho and meta positions or a bicyclic heteroaromatic scaffold are essential to the promising antimicrobial activity of the fluorinated chalcones.

#### 2.2.2. Determination of Minimum Inhibitory Concentration (MIC)

Minimum inhibitory concentration (MIC) is the lowest concentration of the antimicrobial agent that inhibits the visible growth of a microorganism after an overnight incubation. MICs are used to determine the resistance by the diagnostic labs mainly to confirm the resistance. However, frequently MIC is a research tool to determine the in vitro activity of novel natural and synthetic compounds. The two most potent compounds that emerged out of this study, i.e., **A3** and **B3** were further evaluated against all the six microbial strains by serial tube dilution to assess their minimum inhibitory concentration by serial tube dilution method (Table 3). Both the compounds showed MIC lower than the standard drugs benzyl penicillin and fluconazole against the tested bacterial and fungal strains and were in agreement with the zone of inhibition values. However, compound **A3** showed less activity against *Bacillus subtilis* (MIC = 101 µM) than benzyl penicillin (MIC = 95 µM). Both **A3** and **B3** exhibited nearly equal activity to that of fluconazole against the fungal strains and superior activity compared to benzyl penicillin. Compound **A3** (MIC = 51 µM) and **B3** (MIC = 48 µM) were 1.86- and 1.97-fold more active than benzyl penicillin (MIC = 95 µM) against *Staphylococcus aureus* and **B3** (MIC = 24 µM) was 3.95 times more active than benzyl penicillin against *Bacillus subtilis*. Against *Escherichia coli* and *Proteus vulgaris*, **A3** was 7.64 times more active than benzyl penicillin whereas **B3** was 7.95 and 3.97 times more active. These results show that the activity of **A3** containing a trifluoromethyl group favored the Gram-negative bacteria and **B3** with a trifluoromethoxy group favored Gram-positive bacteria. The obtained results were interesting and called for a synthesis and evaluation of other analogues to improve the potency.

#### 2.2.3. Cytotoxicity Studies

Compounds **A3** and **B3** were evaluated for their cytotoxicity study on normal human liver calls and were found to have an IC_50_ value greater than 50 µg/mL suggesting that the compounds were non-toxic against the tested normal human liver cell lines (Table 4).

## 3. Materials and Methods

### 3.1. Chemicals and Instruments

All the chemicals used were of analytical grade and purchased from commercial sources. The organic solvents such as methanol, hexane, and ethyl acetate were of spectral grade and were used as such without further purification. Anhydrous methanol was obtained by fractional distillation and stored over type 4A° molecular sieves. Some of the solvents were purchased from local manufacturers and some from S.D. Fine Chem. Ltd., Mumbai, India. All the chemicals used in the synthesis were obtained from standard commercial sources. TLC chromatography was carried out on Merck grade precoated TLC silica gel 60 F_254_ plates (Merck KGaA, Darmstadt, Germany) and the spots were visualized under a UV lamp. 4′-trifluoromethylacetophenone and 4′-trifluoromethoxyacetophenone were obtained from Thermo Fisher Scientific-Alfa Aesar (Powai, Mumbai, India). Aldehydes were procured from Avra synthesis Pvt. Ltd. (Hyderabad, India). The melting points were determined in open capillaries, using a digital melting point apparatus (EZmelt, Stanford Research Systems) (expressed in °C) and are uncorrected. FT-IR spectra were scanned using Bruker OPUS 8.0 (BRUKER biospin International AG., Zug) and the ^1^H- and ^13^C-NMR spectra of the compound were recorded on a Bruker 400 Avance NMR spectrophotometer using Tetramethylsilane (TMS) as an internal standard (values are expressed in *δ* ppm). Mass spectra were recorded on SHIMADZU Lab Solution (ESI-MS) spectrometer at Indian Institute of Chemical Technology, Hyderabad, India (refer to the supplementary material).

### 3.2. Synthesis

#### General Procedure for Synthesis

**Scheme 1:**
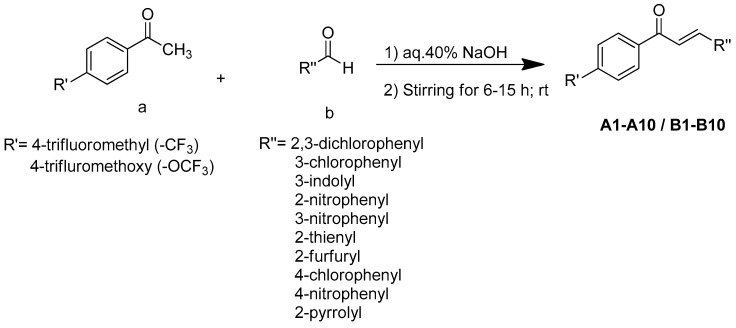
Synthesis of series-A (**A1**–**A10**) and series-B (**B1**–**B10**) fluorinated chalcones.

The two series of chalcones were prepared (Scheme 1) by following Claisen–Schmidt condensation reaction [36]. Initially, 1 mmol of the ketone (4′-trifluromethyacetophenone/4′-trifluromethoxyacetophenone) was dissolved in 8 mL of ethanol. To the above solution, 1 mmol of the corresponding aldehyde was added and then 7.5 mL of 40% sodium hydroxide solution was added dropwise and stirred on a magnetic stirrer at room temperature for about 6–15 h. The progress and the completion of the reaction was monitored on precoated silica gel-G TLC plates and the spots on the plates were visualized using UV lamp and iodine vapors. After the completion of the reaction, the contents of the reaction mixture were transferred into a beaker containing crushed ice and then the mixture was neutralized with 50% hydrochloric acid, which resulted in the separation of the crude precipitate of the chalcone. The precipitate was filtered under vacuum, washed thoroughly with HPLC grade water, and dried in a desiccator. The dried crude mixture was further subjected to column chromatographic purification to obtain the pure product. Column chromatography was performed on 100–200-mesh silica gel as the stationary phase and a 1:15 ratio of hexane and ethyl acetate as mobile phase.

A1: Synthesis of (*E*)-3-(2″,3″-dichlorophenyl)-1-[4′-(trifluoromethyl)phenyl]prop-2-en-1-one: Yield: 95%; m.p. 76 °C; R*_f_* = 0.81 (30% Ethyl acetate in Hexane); FT-IR (KBr, cm^−1^): 1660 (C=O), 1602 (C=C Ar), 1502 (CH=CH), 1255 (-CF_3_), 3011 (Ar C-H stretching), 832 (C-Cl); ^1^H NMR (400 MHz, CDCl_3_, ppm), δ: 7.76 (1H, d, *J* = 16.3 CO-CH=), 8.11 (1H, d, *J* = 16.3, Ar-CH=), 8.12–8.22 (4H, m, C-2′, 3′, 5′, 6′, Ar-H), 6.97–7.74 (3H, m, C- 4″, 5″, 6″, Ar-H); ESI-MS: 346.13 [M + H]^+^.

A2: Synthesis of (*E*)-3-(3″-chlorophenyl)-1-[4′-(trifluoromethyl)phenyl]prop-2-en-1-one: Yield: 90%; m.p. 68 °C; R*_f_* = 0.8 (30% Ethyl acetate in Hexane); FT-IR (KBr, cm^−1^): 1651 (C=O), 1611 (C=C Ar), 1507 (CH=CH), 1252 (-CF_3_), 3010 (Ar C-H stretching), 846 (C-Cl); ^1^H NMR (400 MHz, CDCl_3_, ppm), *δ:* 7.77 (1H, d, *J* = 16.3 Hz CO-CH=), 8.11 (1H, d, *J* = 16.2 Hz, Ar-CH=), 8.12–8.22 (4H, m, C-2′, 3′, 5′, 6′, Ar-H), 7.09–7.74 (4H, m, C-3″, 4″, 5″, 6″, Ar-H); ESI-MS: 311.13 [M + H]^+^.

A3: Synthesis of (*E*)-3-(1″H-indol-3″-yl)-1-[4′-(trifluoromethyl)phenyl]prop-2-en-1-one: Yield: 50%; m.p. 62 °C; R*_f_* = 0.9 (30% Ethyl acetate in Hexane); FT-IR (KBr, cm^−1^): 1695 (C=O), 1611 (C=C Ar), 1573 (CH=CH), 1263 (-CF_3_), 3010 (Ar C-H stretching), 3230 (-NH); ^1^H NMR (400 MHz, CDCl_3_, ppm): *δ*: 7.77 (1H, d, *J* = 16.3 Hz, -CO-CH=), 8.13 (1H, d, *J* = 15.9 Hz, Ar-CH=), 8.78 (1H, s, -NH), 7.94-8.14 (4H, m, C-2′, 3′, 5′, 6′, Ar-H), 7.05-7.61 (5H, m, C-2″, 4″, 5″, 6″, 7″, Ar-H); ^13^C NMR (125 MHz, CDCl3, ppm): 189.93 (C-1), 128.60 (C-2), 142.02 (C-3), 140.11 (C-1′), 128.60 (C-2′ and 6′), 125.59 (C-3′ and 5′), 137.30 (C-4′), 123.77 (C-4,′), 129.52 (C-6′), 112.06 (C-1″), 125.30 (C-2″), 130.30 (C-4″), 114.06 (C-5″), 120.71 (C-6″), 122.02 (C-7″), 117.44 (C-8″), 125.56 (C-9″); ESI-MS: 316.30 [M + H]^+^; ^19^F NMR (376 MHz, CDCl_3_): δ: 63.27 (3F, s).

A4: Synthesis of (*E*)-3-(2″-nitrophenyl)-1-[4′-(trifluoromethyl)phenyl]prop-2-en-1-one: Yield: 60%; m.p. 70 °C; R*_f_* = 0.7 (30% Ethyl acetate in Hexane); FT-IR (KBr, cm^−1^): 1646 (C=O), 1602 (C=C Ar), 1514 (CH=CH), 1249 (-CF_3_), 3010 (Ar C-H stretching), 1461 (NO_2_); ^1^H NMR (400 MHz, CDCl_3_, ppm): *δ*: 7.77 (1H, d, *J* = 17.1 Hz, -CO-CH=), 8.11 (1H, d, *J* = 16 Hz, Ar-CH=), 8.12–8.51 (4H, m, C-2′, 3′, 5′, 6′, Ar-H), 7.70–8.01 (4H, m, C-3″, 4″, 5″, 6″, Ar-H); ESI-MS: 322.11 [M + H]^+^.

A5: Synthesis of (*E*)-3-(3″-nitrophenyl)-1-[4′-(trifluoromethyl)phenyl]prop-2-en-1-one: Yield: 60%; m.p. 85 °C; R*_f_* = 0.5 (30% Ethyl acetate in Hexane); FT-IR (KBr, cm^−1^): 1659 (C=O), 1623 (C=C Ar), 1536 (CH=CH), 1250 (-CF_3_), 3010 (Ar C-H stretching), 1446 (NO_2_); ^1^H NMR (400 MHz, CDCl_3_, ppm): 7.92 (1H, d, *J* = 15.7 Hz, -CO-CH=), 8.15 (1H, d, *J* = 16 Hz, Ar-CH=), 7.82–8.65 (4H, m, C-2′, 3′, 5′, 6′, Ar-H), 7.68–7.89 (4H, m, C-2″, 3″, 5″, 6″, Ar-H); ESI-MS: 322.25 [M + H]^+^.

A6: Synthesis of (*E*)-3-(thiophen-2″-yl)-1-[4′-(trifluoromethyl)phenyl]prop-2-en-1-one: Yield: 80%; m.p. 86 °C; R*_f_* = 0.76 (30% Ethyl acetate in Hexane); FT-IR (KBr, cm^−1^): 1658 (C=O), 1504 (CH=CH), 1614 (C=C Ar), 3121 (Ar C-H stretching), 1250 (-CF_3_), 621 (C-S); ^1^H NMR (400 MHz, CDCl_3_, ppm): *δ*: 7.79 (1H, d, *J* = 16 Hz, -CO-CH=), 8.14 (1H, d, *J* = 16.2, 4.9 Ar-CH=), 7.80–8.12 (4H, m, C-2′, 3′, 5′, 6′, Ar-H), 7.17–7.77 (4H, m, C-3″, 4″, 5″, Ar-H); ESI-MS: 283.36 [M + H]^+^.

A7: Synthesis of (*E*)-3-(furan-2″-yl)-1-[4′-(trifluoromethyl)phenyl]prop-2-en-1-one: Yield: 80%; m.p. 72 °C; R*_f_* = 0.71 (30% Ethyl acetate in Hexane); FT-IR (KBr, cm^−1^): 1658 (C=O), 1514 (CH=CH), 1631 (C=C Ar), 3111 (Ar C-H stretching), 1254 (-CF_3_), 1700 (C-O); ^1^H NMR (400 MHz, CDCl_3_, ppm), *δ*: 7.75 (1H, d, *J* = 16.1 Hz, -CO-CH=), 8.14 (1H, d, *J* = 16.1 Hz, Ar-CH=), 7.59–8.12 (4H, m, C-2′, 3′, 5′, 6′, Ar-H), 6.52–7.53 (3H, m, C-3″, 4″, 5″, Ar-H); ESI-MS: 227.68 [M + H]^+^.

A8: Synthesis of (*E*)-3-(4″-chlorophenyl)-1-[4′-(trifluoromethyl)phenyl]prop-2-en-1-one: Yield: 90%; m.p. 68 °C; R*_f_* = 0.72 (30% Ethyl acetate in Hexane); FT-IR (KBr, cm^−1^): 1656 (C=O), 1609 (C=C Ar), 1505 (CH=CH), 1247 (-CF_3_), 3001 (Ar C-H stretching), 841 (C-Cl); ^1^H NMR (400 MHz, CDCl_3_, ppm): *δ*: 7.78 (1H, d, *J* = 16.3 Hz, -CO-CH=), 8.14 (1H, d, *J* = 16.2 Hz, Ar-CH=), 7.77–8.13 (4H, m, C-2′, 3′, 5′, 6′, Ar-H), 7.43–7.61 (4H, m, C-2″, 4″, 5″, 6″, Ar-H); ESI-MS: 311.62 [M + H]^+^.

A9: Synthesis of (*E*)-3-(4″-nitrophenyl)-1-[4′-(trifluoromethyl)phenyl]prop-2-en-1-one: Yield: 61%; m.p. 82 °C; R*_f_* = 0.7 (30% Ethyl acetate in Hexane); FT-IR (KBr, cm^−1^): 1654 (C=O), 1621 (C=C Ar), 1512 (CH=CH), 1252 (-CF_3_), 3010 (Ar C-H stretching), 1430 (NO_2_); ^1^H NMR (400 MHz, CDCl_3_, ppm): *δ*: 7.85 (1H, d, *J* = 17 Hz, -CO-CH=), 8.11 (1H, d, *J* = 16 Hz, Ar-CH=), 7.68–7.82 (4H, m, C-2′, 3′, 5′, 6′, Ar-H), 8.12–8.32 (4H, m, C-2″, 3″, 5″, 6″, Ar-H); ESI-MS: 322.11 [M + H]^+^.

A10: Synthesis of (*E*)-3-(1″H-pyrrol-2-yl)-1-[4′-(trifluoromethyl)phenyl]prop-2-en-1-one: Yield: 80%; m.p. 75 °C; R*_f_* = 0.68 (30% Ethyl acetate in Hexane); FT-IR (KBr, cm^−1^): 1650 (C=O), 1501 (CH=CH), 1618 (C=C Ar), 1242 (-CF_3_), 1370 (C-N), 3115 (Ar C-H stretching); ^1^H NMR (400 MHz, CDCl_3_, ppm), *δ*: 7.77 (1H, d, *J* = 16 Hz, -CO-CH=), 8.11 (1H, d, *J* = 16 Hz, Ar-CH=), 7.74–8.23 (4H, m, C-2′, 3′, 5′, 6′, Ar-H), 6.08–6.96 (3H, m, C-3″, 4″, 5″, Ar-H), 9.93 (1H, s, -NH); ESI-MS: 266.23 [M + H]^+^.

B1: Synthesis of (*E*)-3-(2″,3″-dichlorophenyl)-1-[4′-(trifluoromethoxy)phenyl]prop-2-en-1-one: Yield: 86%; m.p. 70 °C; R*_f_* = 0.8 (30% Ethyl acetate in Hexane); FT-IR (KBr, cm^−1^): 1664 (C=O), 1624 (Ar C=C), 1520 (CH=CH), 1242 (-CF_3_), 3023 (Ar C-H stretching), 1166 (-C-O-) 838 (C-Cl); ^1^H NMR (400 MHz, CDCl_3_, ppm), δ: 7.58 (1H, d, *J* = 16.3 Hz, CO-CH=), 8.03 (1H, d, *J* = 16.3 Hz, Ar-CH=), 7.64–8.22 (4H, m, C-2′, 3′, 5′, 6′, Ar-H), 6.97–7.62 (3H, m, C- 4″, 5″, 6″, Ar-H); ESI-MS: 362.12 [M + H]^+^.

B2: Synthesis of (*E*)-3-(3″-chlorophenyl)-1-[4′-(trifluoromethoxy)phenyl]prop-2-en-1-one: Yield: 84%; m.p. 65 °C; R*_f_* = 0.81 (30% Ethyl acetate in Hexane); FT-IR (KBr, cm^−1^): 1648 (C=O), 1607 (Ar C=C), 1512 (CH=CH), 1232 (-CF3), 3015 (Ar C-H stretching), 1156 (-C-O-), 840 (C-Cl); ^1^H NMR (400 MHz, CDCl_3_, ppm), *δ*: 7.37 (1H, d, *J* = 16.3 Hz, -CO-CH=), 8.02 (1H, d, *J* = 16.2 Hz, Ar-CH=), 7.70–8.22 (4H, m, C-2′, 3′, 5′, 6′, Ar-H), 7.05–7.68 (4H, m, C-3″, 4″, 5″, 6″, Ar-H); ESI-MS: 343.58 [M + H]^+^.

B3: Synthesis of (*E*)-3-(1″H-indol-3″-yl)-1-[4′-(trifluoromethoxy)phenyl]prop-2-en-1-one: Yield: 45%; m.p. 67 °C; R*_f_* = 0.74 (30% Ethyl acetate in Hexane); FT-IR (KBr, cm^−1^): 1651 (C=O), 1602 (Ar C=C), 1595 (CH=CH), 1521 (C-N), 3681 (-NH), 1206 (-OCF_3_), 3362 (Ar C-H stretching), 1248 (-C-O-); ^1^H NMR (400 MHz, CDCl_3_, ppm): *δ* 7.53 (1H, d, *J* = 16.3 Hz, -CO-CH=), 8.05 (1H d, *J* = 15.9, Ar-CH=), 8.78 (1H, s, -NH), 7.38–8.02 (4H, m, C-2′, 3′, 5′, 6′, Ar-H), 7.05–7.66 (5H, m, C-2″, 4″, 5″, 6″, 7″, Ar-H);

^13^C NMR (125 MHz, CDCl3, ppm): 189.97 (C-1), 125.33 (C-2), 137.37 (C-3), 130.23 (C-1′), 130.71 (C-2′ and 6′), 121.88 (C-3′ and 5′), 139.61 (C-4′), 121.88 (C-4,′), 112.08 (C-1″), 123.63 (C-2″), 137.35 (C-4″),114.41 (C-5″), 120.47 (C-6″), 122.69 (C-7″), 117.81 (C-8″), 123.63 (C-9″); ESI-MS: 332.20 [M + H]^+^; ^19^F NMR (376 MHz, CDCl_3_): *δ*, 58.64 (3F, s).

B4: Synthesis of (*E*)-3-(2″-nitrophenyl)-1-[4′-(trifluoromethoxy)phenyl]prop-2-en-1-one: Yield: 56%; m.p. 69 °C; R*_f_* = 0.87 (30% Ethyl acetate in Hexane); FT-IR (KBr, cm^−1^): 1640 (C=O), 1632 (Ar C=C), 1504 (CH=CH),1232 (-CF_3_), 3012 (Ar C-H stretching), 1126 (-C-O-), 1462 (NO_2_); ^1^H NMR (400 MHz, CDCl_3_, ppm): *δ*: 7.85 (1H, d, *J* = 17 Hz, -CO-CH=), 8.28 (1H, d, *J* = 16 Hz, Ar-CH=), 8.27–8.51 (4H, m, C-2′, 3′, 5′, 6′, Ar-H), 7.05–8.25 (4H, m, C-3″, 4″, 5″, 6″, Ar-H); ESI-MS: 338.08 [M + H]^+^.

B5: Synthesis of (*E*)-3-(3″-nitrophenyl)-1-[4′-(trifluoromethoxy)phenyl]prop-2-en-1-one: Yield: 55%; m.p. 71 °C; R*_f_* = 0.72 (30% Ethyl acetate in Hexane); FT-IR (KBr, cm^−1^): 1659 (C=O), 1626 (Ar C=C), 1532 (CH=CH), 1248 (-CF_3_), 3019 (Ar C-H stretching), 1127 (-C-O-), 1448 (NO_2_); ^1^H NMR (400 MHz, CDCl_3_, ppm): *δ*: 7.06 (1H, d, *J* = 16.3 Hz, CO-CH=), 8.05 (1H, d, *J* = 16.2 Hz, Ar-CH=), 7.76–8.03 (4H, m, C-2′, 3′, 5′, 6′, Ar-H), 7.08–7.79 (4H, m, C-2″, 4″, 5″, 6″, Ar-H); ESI-MS: 338.35 [M + H]^+^.

B6: Synthesis of (*E*)-3-(thiophen-2″-yl)-1-[4′-(trifluoromethoxy)phenyl]prop-2-en-1-one: Yield: 76%; m.p. 82 °C; R*_f_* = 0.68 (30% Ethyl acetate in Hexane); FT-IR (KBr, cm^−1^): 1656 (C=O), 1512 (CH=CH), 1610 (Ar C=C), 3126 (Ar C-H stretching), 1262 (-CF_3_), 1156 (-C-O-), 620 (C-S); ^1^H NMR (400 MHz, CDCl_3_, ppm): *δ*: ), 7.05 (1H, d, *J* = 16, -CO-CH=), 8.05 (1H, d, *J* = 6.2, 4.9 Ar-CH=), 7.20–8.02 (4H, m, C-2′, 3′, 5′, 6′, Ar-H), 7.17–7.79 (3H, m, C-3″, 4″, 5″, Ar-H); ESI-MS: 299.16 [M + H]^+^.

B7: Synthesis of (*E*)-3-(furan-2″-yl)-1-[4′-(trifluoromethoxy)phenyl]prop-2-en-1-one: Yield: 78%; m.p. 68 °C; R*_f_* = 0.66 (30% Ethyl acetate in Hexane); FT-IR (KBr, cm^−1^): 1651 (C=O), 1524 (CH=CH), 1635 (Ar C=C), 3111 (Ar C-H stretching), 1250 (-CF_3_), 1159 (-C-O-); ^1^H NMR (400 MHz, CDCl_3_, ppm), *δ*: 7.07 (1H, d, *J* = 16.1 Hz, -CO-CH=), 8.05 (1H, d, *J* = 16.1 Hz, Ar-CH=), 7.47–8.05 (4H, m, C-2′, 3′, 5′, 6′, Ar-H), 6.52–7.59 (3H, m, C-3″, 4″, 5″, Ar-H); ESI-MS: 283.47 [M + H]^+^.

B8: Synthesis of (*E*)-3-(4″-chlorophenyl)-1-[4′-(trifluoromethoxy)phenyl]prop-2-en-1-one: Yield: 84%; m.p. 75 °C; R*_f_* = 0.64 (30% Ethyl acetate in Hexane); FT-IR (KBr, cm^−1^): 1645 (C=O), 1629 (Ar C=C), 1512 (CH=CH), 1237 (-CF_3_), 3011 (Ar C-H stretching), 1155 (-C-O-), 837 (C-Cl); ^1^H NMR (400 MHz, CDCl_3_, ppm): *δ*: 8.33 (1H, d, *J* = 16 Hz, Ar-CH=), 7.05 (1H d, *J* = 15.7 Hz, -CO-CH=), 7.92–8.65 (4H, m, C-2′, 3′, 5′, 6′, Ar-H), 7.06–7.89 (4H, m, C-2″, 3″, 5″, 6″, Ar-H); ESI-MS: 343.19 [M + H]^+^.

B9: Synthesis of (*E*)-3-(4″-nitrophenyl)-1-[4′-(trifluoromethoxy)phenyl]prop-2-en-1-one: Yield: 57%; m.p. 68 °C; R*_f_* = 0.68 (30% Ethyl acetate in Hexane); FT-IR (KBr, cm^−1^): 1652 (C=O), 1614 (Ar C=C), 1510 (CH=CH), 1242 (-CF_3_), 3016 (Ar C-H stretching), 1122 (-C-O-), 1436 (NO_2_); ^1^H NMR (400 MHz, CDCl_3_, ppm): *δ*: 7.06 (1H, d, *J* = 16 Hz, -CO-CH=), 8.03 (1H, d, *J* = 16 Hz, Ar-CH=), 7.05–8.04 (4H, m, C-2′, 3′, 5′, 6′, Ar-H), 7.82–8.32 (4H, m, C-2″, 3″, 5″, 6″, Ar-H); ESI-MS [M + H]^+^: 338.72 and 339.72.

B10: Synthesis of (*E*)-3-(1″H-pyrrol-2-yl)-1-[4′-(trifluoromethoxy)phenyl]prop-2-en-1-one: Yield: 67%; m.p. 59 °C; R*_f_* = 0.70 (30% Ethyl acetate in Hexane); FT-IR (KBr, cm^−1^): 1646 (C=O), 1516 (CH=CH), 1621 (Ar-C=C-), 1232 (-CF_3_), 1336 (C-N), 3121 (Ar C-H stretching), 1166 (-C-O-); ^1^H NMR (400 MHz, CDCl_3_, ppm), *δ*: 7.07 (1H, d, *J* = 16.6 Hz, -CO-CH=), 8.03 (1H, d, *J*= 16.6 Hz, Ar-CH=), 7.08–8.23 (4H, m, C-2′, 3′, 5′, 6′, Ar-H), 6.09–7.06 (3H, m, C-3″, 4″, 5″, Ar-H), 9.93 (1H, s, -NH); ESI-MS: 282.02 [M + H]^+^.

### 3.3. Biological Activity Studies

#### 3.3.1. Antimicrobial Screening

The antimicrobial activity was performed against six different antimicrobial strains. The organisms selected are listed below:
Gram-positive bacteria *Bacillus subtilis* (NCIM-2079), *Staphylococcus aureus* (NCIM-2079)Gram-negative bacteria*Escherichia coli* (NCIM-2065), *Proteus vulgaris* (NCIM-2027)Fungi*Candida albicans* (MDCC-227), *Aspergillus niger* (MTCC 5889)

Glassware was cleaned and kept in a hot air oven at 160 °C for 2 h. The media were sterilized and the solutions of standard drugs (Benzyl penicillin and fluconazole) and A and B series of compounds were kept ready. In the meantime, nutrient agar medium was prepared (composition: peptone 0.5%, meat extract 0.3%, sodium chloride 0.5%, agar 2%, distilled water to make up to 100 mL, and pH adjusted to 7.2). The weighed quantities of peptone, meat extract, and sodium chloride were dissolved in 1000 mL of distilled water and the pH of the medium was adjusted to 7.2. After the dissolution of agar, the medium was distributed into conical flask each containing 25 mL. The media and sterile water were sterilized by autoclaving at 121 °C temperature and 15 lbs/sq. inch pressure for 20 min. Petri plates, test tubes, pipettes, and borer required for experiment were sterilized by dry heat sterilization using hot air oven. Cultures of respective organisms (18 h old) were taken and suspension of these microorganisms was made using sterile water. Later, 0.5 mL of this suspension was used as inoculum and pour plate technique was used for estimation of bacterial load in each sample. The inoculated agar medium was poured into sterile 10 cm-diameter petri dishes and the medium in the plates was allowed to solidify. The solutions of the test compounds in concentrations of 0.1 µg/mL were prepared in DMSO. The cups of 5 mm diameter were prepared using a borer in the corresponding medium. In each plate, 5 wells were prepared. Three wells were for test compounds, one for standard compound and another one was used as control. In each well, samples were poured and then plates were left for 45 min in a refrigerator for diffusion. After incubation for 18 h at 37 °C, the plates were examined for inhibition zones. The experiments were done in triplicate on the same day with the same conditions in order to minimize the experimental errors. The zone of inhibition values was calculated using vernier caliper and represented as a mean of three values and standard deviation was applied [10].

#### 3.3.2. Determination of Minimum Inhibitory Concentration (MIC)

MIC has become the current standard test for antibiotic sensitivity testing because it produces more pertinent information on minimal dosages. Hence, we determined the MIC of selected compounds, i.e., **A3** and **B3** employing the protocol prescribed in our previously published papers [37].

#### 3.3.3. Cytotoxicity Studies

The most potent compounds **A3** and **B3** out of the 20 compounds were tested in vitro for their cytotoxic activity on L02 (human normal liver cell line) by employing MTT assay according to Mosmann’s method as described in our previous paper [38]. The MTT assay is based on the reduction of the soluble MTT (0.5 mg mL^−1^, 100 µL) into a blue–purple formazan product, mainly by mitochondrial reductase activity inside living cells (Mosmann T et al., 1983). The cells used in cytotoxicity assay were cultured in RPMI 1640 medium supplemented with 10% fetal calf serum, penicillin, and streptomycin at 37 °C and humidified at 5% CO_2_. Briefly, cells were placed on 96-well plates at 100 µL total volume with a density of 1–2.5 × 10^4^ cells per mL and were allowed to adhere for 24 h before treatment with tested drugs in DMSO solution (10^−5^, 10^−6^, 10^−7^ mol L^−1^ final concentration). Triplicate wells were treated with media and agents. Cell viability was assayed after 96 h of continuous drug exposure with a tetrazolium compound. The supernatant medium was removed, and 150 µL of DMSO solution was added to each well. The plates were gently agitated using mechanical plate mixer until the color reaction was uniform and OD570 was determined using micro plate reader. The 50% inhibitory concentration (IC_50_) was defined as the concentration that reduced the absorbance of the untreated wells by 50% of the vehicle in the MTT assay. Assays were performed in triplicate on three independent experiments. The results showed good reproducibility between replicate wells with standard errors below 10%.

## 4. Conclusions

In this paper, we described the design, synthesis, characterization, and antimicrobial screening of 20 new fluorinated chalcones. Most of the compounds displayed promising antibacterial and antifungal activities and two compounds bearing indolyl scaffold, i.e., **A3** and **B3**, showed potential activities and were also non-toxic on the normal human liver cell lines (L02). Additionally, compounds bearing electron-withdrawing nitro or the chloro substituents at the ortho or the meta position showed valuable antimicrobial activity. Hence, these compounds are novel lead compounds identified through our study for the development of novel agents against bacterial and fungal infections. Although the present study gave us some lead molecules, future investigation needs to be done by synthesizing analogues of **A3** and **B3** by replacing the indole scaffold of **A3** and **B3** with benzofuran and benzothiophene moieties as well as by substituting more lipophilic -SCF_3_ for -OCF_3_ in **B3**. Further, a plausible mode of action for the proposed activities needs to be investigated.

## Figures and Tables

**Table 1:**
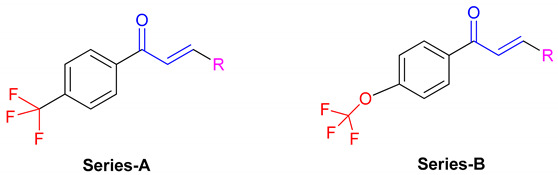
Antibacterial (zone of inhibition in mm) ^a, b^ of trifluoromethyl and trifluoromethoxy substituted chalcone derivatives (A1–A10 and B1–B10).

Entry	Microorganisms				Entry	Microorganisms			
Compound Code	*S.aureus*	*B.subtilis*	*E.coli*	*P.vulgaris*	Compound Code	*S.aureus*	*B.subtilis*	*E.coli*	*P.vulgaris*
**A1**	20.08 ± 0.02	21.16 ± 0.15	17.07 ± 0.02	20.02 ± 0.03	**B1**	21.06 ± 0.04	22.2 ± 0.11	18.05 ± 0.01	21.09 ± 0.12
**A2**	18.13 ± 0.04	19.1 ± 0.26	13.11 ± 0.09	19.02 ± 0.02	**B2**	18.04 ± 0.03	20.46 ± 0.25	14.04 ± 0.00	20.03 ± 0.03
**A3**	**25.05 ± 0.01**	**26.36 ± 0.32**	**20.03 ± 0.02**	**23.02 ± 0.02**	**B3**	**26.46 ± 0.04**	**29.43 ± 0.30**	**22.02 ± 0.02**	**21.02 ± 0.02**
**A4**	20.06 ± 0.32	20.2 ± 0.17	16.04 ± 0.04	20.03 ± 0.03	**B4**	21.56 ± 0.04	20.18 ± 0.14	17.21 ± 0.20	20.04 ± 0.04
**A5**	19.2 ± 0.81	20.13 ± 0.11	16.05 ± 0.04	20.06 ± 0.01	**B5**	23.53 ± 0.04	22.02 ± 0.02	17.20 ± 0.24	20.04 ± 0.03
**A6**	18.10 ± 0.07	17.13 ± 0.15	15.18 ± 0.27	18.06 ± 0.01	**B6**	20.53 ± 0.04	19.61 ± 0.32	16.05 ± 0.03	17.04 ± 0.00
**A7**	19.23 ± 0.04	18.66 ± 0.20	17.03 ± 0.02	19.20 ± 0.25	**B7**	21.36 ± 0.16	20.41 ± 0.33	15.05 ± 0.01	18.02 ± 0.01
**A8**	15.23 ± 0.21	10.16 ± 0.20	15.05 ± 0.04	14.22 ± 0.22	**B8**	15.15 ± 0.05	16.3 ± 0.10	9.03 ± 0.01	12.05 ± 0.01
**A9**	15.5 ± 0.16	17.13 ± 0.11	15.01 ± 0.02	12.09 ± 0.98	**B9**	16.3 ± 0.02	15.07 ± 0.04	8.06 ± 0.01	13.04 ± 0.52
**A10**	12.1 ± 0.08	11.36 ± 0.05	13.03 ± 0.03	11.18 ± 0.07	**B10**	14.1 ± 0.04	15.21 ± 0.21	9.46 ± 0.25	10.05 ± 0.01
**Benzyl penicillin**	24.06 ± 0.05	27.02 ± 0.02	14.05 ± 0.05	19.04 ± 0.03	**Benzyl penicillin**	24.06 ± 0.05	27.02 ± 0.02	14.05 ± 0.05	19.04 ± 0.03

**^a^** Mean value ± SD (standard deviation from three experiments); ^b^ Bold value indicates best compounds.

**Table 2:**
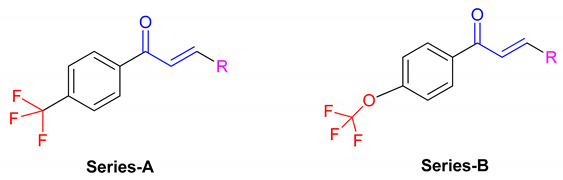
Antifungal activity (zone of inhibition in mm) ^a^ of trifluoromethyl and trifluoromethoxy substituted chalcone derivatives (A1–A10 and B1–B10).

Entry	Microorganisms		Entry	Microorganisms	
Compound Code	*C. albicans*	*A. niger*	Compound Code	*C. albicans*	*A.niger*
**A1**	18.03 ± 0.01	20.03 ± 0.02	**B1**	19.09 ± 0.01	21.04 ± 0.01
**A2**	15.05 ± 0.03	19.03 ± 0.03	**B2**	17.08 ± 0.01	20.05 ± 0.01
**A3**	20.05 ± 0.04	25.06 ± 0.01	**B3**	22.05 ± 0.03	26.07 ± 0.01
**A4**	17.07 ± 0.01	20.07 ± 0.01	**B4**	16.07 ± 0.01	20.05 ± 0.04
**A5**	17.05 ± 0.01	20.07 ± 0.01	**B5**	15.07 ± 0.01	20.02 ± 0.02
**A6**	10.08 ± 0.01	14.05 ± 0.04	**B6**	16.08 ± 0.01	15.04 ± 0.03
**A7**	11.07 ± 0.01	15.04 ± 0.04	**B7**	15.07 ± 0.01	17.08 ± 0.01
**A8**	11.03 ± 0.02	6.01 ± 0.01	**B8**	11.05 ± 2.32	12.05 ± 0.01
**A9**	12.05 ± 0.01	12.03 ± 0.02	**B9**	10.21 ± 0.24	11.05 ± 0.01
**A10**	13.04 ± 0.03	21.05 ± 0.03	**B10**	10.09 ± 0.01	12.05 ± 0.02
**Fluconazole**	19.05 ± 0.04	24.41 ± 0.52	**Fluconazole**	19.05 ± 0.04	24.41 ± 0.52

**^a^** Mean value ± SD (standard deviation from three experiments).

**Table 3:** Minimum inhibitory concentration (MIC in µM) of compounds **A3** and **B3.**

Entry	*Staphylococcus aureus*	*Bacillus subtilis*	*Escherichia coli*	*Proteus vulgaris*	*Candida albicans*	*Aspergillus niger*
**A3**	51	101	25	25	50	25
**B3**	48	24	24	48	48	24
**Benzyl Penicillin**	95	95	191	191	-	-
**Fluconazole**	-	-	-	-	52	26

**Table 4:** Cytotoxicity of compounds **A3** and **B3** against human normal liver cells (IC_50_, µg/mL) ^a.^

S. No	Compounds	Human Normal Liver Cells (L02)
1	**A3**	>50
2	**B3**	>50

^a^ Mean value from three experiments.

## Supplementary Materials

The supplementary materials are available online at https://www.mdpi.com/1424-8247/13/11/375/s1.

## References

[1] Richard J.F., Yitzhak T. (2014). Antibiotics and bacterial resistance in the 21st century. Perspect. Med. Chem..

[2] Halling-Sørensen B. (2001). Inhibition of aerobic growth and nitrification of bacteria in sewage sludge by antibacterial agents. Arch. Environ. Contam. Toxicol..

[3] Harish C., Parul B., Archana Y., Babita P., Abhay P.M., Anant R.N. (2017). Antimicrobial resistance and the alternative resources with special emphasis on plant-based antimicrobials-a review. Plants.

[4] Francesca P., Patrizio P., Annalisa P. (2015). Antimicrobial resistance: A global multifaceted phenomenon. Pathog. Glob. Health.

[5] Bingyun L., Thomas J.W. (2018). Bacteria antibiotic resistance: New challenges and opportunities for implant-associated orthopedic infections. J Orthop. Res..

[6] Novel Drug Approvals for 2017. https://www.fda.gov/drugs/new-drugs-fda-cders-new-molecular-entities-and-new-therapeutic-biological-products/novel-drug-approvals-2017.

[7] Novel Drug Approvals for 2018. https://www.fda.gov/drugs/new-drugs-fda-cders-new-molecular-entities-and-new-therapeutic-biological-products/novel-drug-approvals-2018.

[8] Wang W., Sun Q. (2020). Novel targeted drugs approved by the NMPA and FDA in 2019. Sig. Transduct. Target Ther..

[9] Nurken B., Mohamad A. (2020). An overview of drug discovery and development. Future Med. Chem..

[10] Lagu S.B., Rajendra P.Y., Srinath N., Afzal B.S. (2019). Synthesis. antibacterial, antifungal antitubercular activities and molecular docking studies of nitrophenyl derivatives. Int. J. Life Sci. Pharma Res..

[11] Zhuang C., Zhang W., Sheng C., Zhang W., Xing C., Miao Z. (2017). Chalcone: A Privileged Structure in Medicinal Chemistry. Chem. Rev..

[12] Maya Z.G., Minh A.N., Sarah K.Z. (2018). Design, synthesis and evaluation of (2-(Pyridinyl)methylene)-1-tetralone chalcones for Anticancer and Antimicrobial Activity. Med. Chem..

[13] Mei L., Prapon W., Mei-Lin G. (2001). Antimalarial alkoxylated and hydroxylated chalones: Structure-activity relationship analysis. J. Med. Chem..

[14] Serdar B., Oztekin A., Derya A.A., Arzu G., Gulay G.D., Ronak H.E., Nizami D. (2016). Synthesis and anti-proliferative activity of fluoro-substituted chalcones. Bioorg. Med. Chem. Lett..

[15] Ramesh C.K., Rita A., Geeta S., Dinesh K., Chetan S., Radhika J., Aneja K.R. (2010). Ecofriendly synthesis and antimicrobial activity of chalcones. Der. Pharma. Chem..

[16] Zhang X., Zhao D.-H., Quan Y.-C., Sun L., Yin X.-M., Guan L.-P. (2010). Synthesis and evaluation of antiinflammatory activity of substituted chalcone derivatives. Med. Chem. Res..

[17] Khaled R.A.A., Heba A.H.E., Samir A.S., Hany A.O. (2015). Synthesis, characterization and biological evaluation of novel 4-fluoro-2-hydroxychalcone derivatives as antioxidant, anti-inflammatory, analgesic agents. J Enzyme Inhib Med Chem..

[18] Shen-Jew W., Cheng-Tsung L., Lo-Ti T., Jing-Ru W., Horng-Huey K., Jih-Pyang W., Chun-Nan L. (2005). Synthetic chalcones as potential anti-inflammatory and cancer chemo preventive agents. Eur. J. Med. Chem..

[19] Nazifi S.I., Farediah A. (2014). Antimicrobial activities of some synthetic flavonoids. J. App. Chem..

[20] Pinki Y., Kashmiri, Lokesh K., Ashwani K., Anil K., Avijit K.P., Rajnish K. (2018). Synthesis, crystal structure and antimicrobial potential of some fluorinated chalcones-1,2,3-triazole conjugates. Eur. J. Med. Chem..

[21] Francesca B., Giovanna A.G., Francesca B., Silvia G., Angela R., Alessandra B., Federica B. (2019). Functionalization of the Chalcone Scaffold for the Discovery of Novel Lead Compounds Targeting Fungal Infections. Molecules.

[22] Man X., Piye W., Fan S., Jiayou J., Rakesh K.P. (2019). Chalcone derivatives and their antibacterial activities: Current development. Bioorg. Chem..

[23] Afzal B.S., Yejella R.P., Shaik S. (2017). Synthesis, Antimicrobial, and Computational Evaluation of Novel Isobutylchalcones as Antimicrobial Agents. Int. J. Med. Chem..

[24] Afzal B.S., Lohitha S.V.K., Puttagunta S.B., Shaik A., Supraja K., Sai H.K. (2019). Synthesis and screening of novel lipophilic diarylpropeones as prospective antitubercular, antibacterial and antifungal agents. Biointerface Res. Appl. Chem..

[25] Serdar B., Oztekin A., Arzu G., Derya A.A., Mahmut U., Busra G.E., Engin K., Aylin D., Gönül A. (2017). Design of potent fluoro-substituted chalcones as antimicrobial agents. J. Enzyme Inhib. Med. Chem..

[26] Kayode L.A., Issac A.B., Adebayo O.O. (2019). Synthesis, Characterization and Antibacterial Activities of New Fluorinated Chalcones. Chem. Afr..

[27] Prasad Y.R., Rao A.L., Rambabu R. (2008). Synthesis and antimicrobial activity of some chalcone derivatives. J. Chem..

[28] Afzal S., Richie R.B., Palleapati K., Srinath N., Venkata K., Shaik S. (2020). Antimicrobial, antioxidant, and anticancer activities of some novel isoxazole ring containing chalcone and dihydropyrazole derivatives. Molecules.

[29] Marcelo N.G., Eugene N.M., Maristela P., Josana C.P., Lucimar P.R., Pedro V.L.C., Carolina H.A., Bruno J.N. (2017). Chalcone derivatives: Promising starting points for drug design. Molecules.

[30] Selvakumar N., Sunil K.G., Malar A.A., Govinda R.G., Shikha S., Sitaram K.M., Jagattaran D., Javed I., Sanjay T. (2007). Synthesis, SAR and antibacterial studies on novel chalcone oxazolidinone hybrids. Eur. J. Med. Chem..

[31] Hans J.B., David B., Stefanie B., Manfred K., Bernd K., Klaus M., Ulrike O., Martin S. (2004). Fluorine in Medicinal Chemistry. Chem. Bio. Chem..

[32] Poonam S., Andrew D.W. (2007). The role of fluorine in medicinal chemistry. J. Enzyme Inhib. Med. Chem..

[33] Sophie P., Peter R.M., Steve S., Veronique G. (2008). Fluorine in Medicinal Chemistry. Chem. Soc. Rev..

[34] William K.H. (2008). The Many Roles for Fluorine in Medicinal Chemistry. J. Med. Chem..

[35] Eric P.G., Kyle J.E., Matthew D.H., David J.D., Nicholas A.M.E., Matthew D.H., David J.D., Nicholas A.M. (2015). Applications of Fluorine in Medicinal Chemistry. J. Med. Chem..

[36] Claisen L., Claparede B.A. (1881). Condensationen von Ketonen mit Aldehyden. Berichte Deutschen Chemischen Gesellschaft..

[37] Shaik A.B., Bhandare R.R., Nissankararao S., Edis Z., Tangirala N.R., Shahanaaz S., Rahman M.M. (2020). Design, facile synthesis and characterization of dichloro substituted chalcones and dihydropyrazole derivatives for their antifungal, antitubercular and antiproliferative activities. Molecules.

[38] Lokesh B.V.S., Prasad Y.R., Shaik A.B. (2019). Novel pyrimidine derivatives from 2,5-dichloro-3-acetylthienyl chalcones as antifungal, antitubercular and cytotoxic agents: Design, synthesis, biological activity and docking study. Asian J. Chem..

