# Supplementary material for: Design, Synthesis, and Antibacterial and Antifungal Activities of Novel Trifluoromethyl and Trifluoromethoxy Substituted Chalcone Derivatives
Source: Pharmaceuticals (Basel). 2020 Nov 9;13(11):375. doi: 10.3390/ph13110375 (PMC7695348; doi:10.3390/ph13110375)
Supplement: Supplementary file 1 [file pharmaceuticals-13-00375-s001.pdf]

Supplementary Data

Design, synthesis, and antibacterial and antifungal activities of novel trifluoromethyl and trifluoromethoxy substituted chalcone derivatives

Lagu Surendra Babu[1,*], Yejella Rajendra Prasad[2], Richie R. Bhandare [3,*] and Afzal B. Shaik [4,*]

FT-IR, ^{1}H NMR, ^{13}C NMR & MS

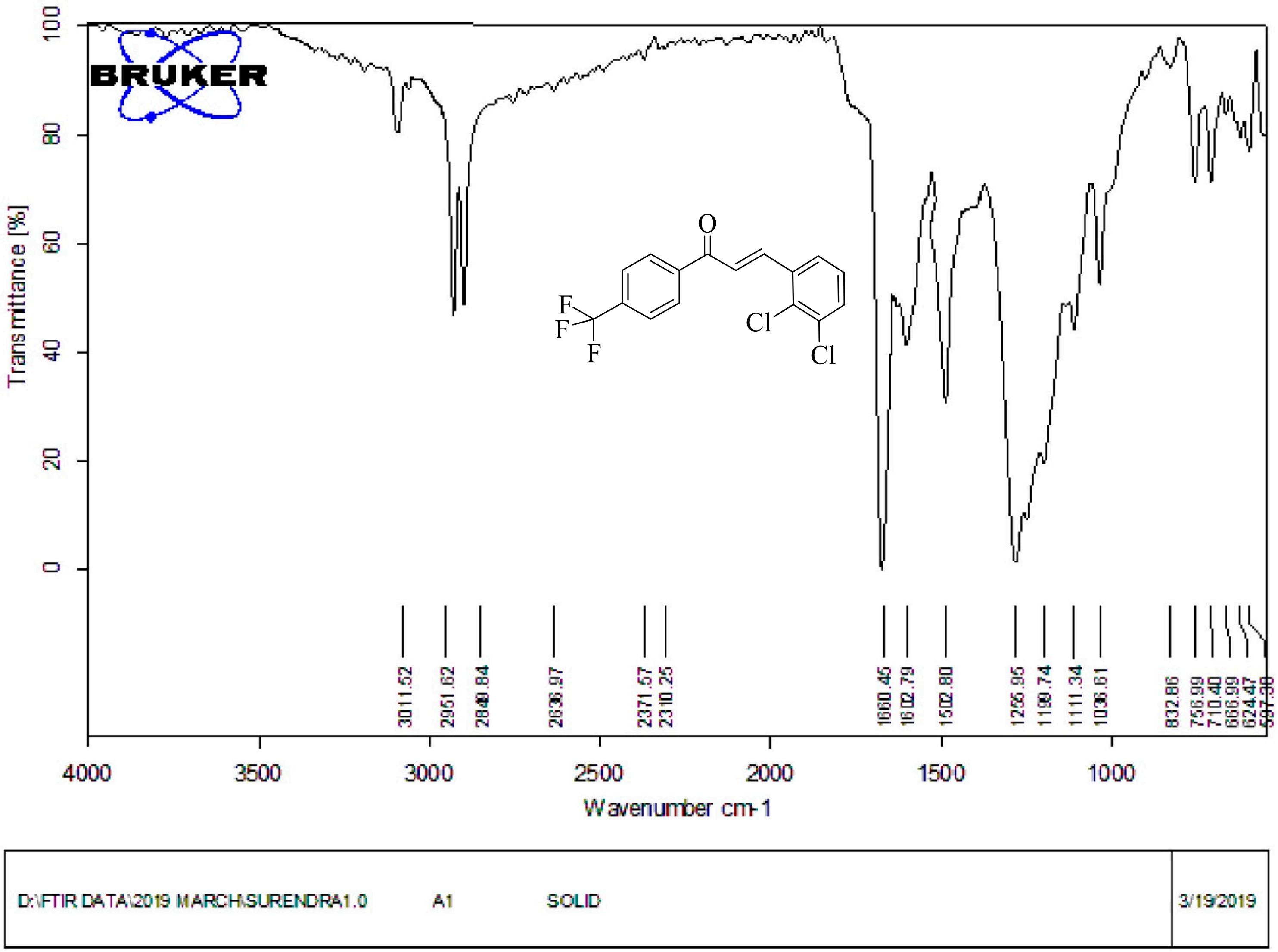

FT-IR Spectrum of *(E)*-3-(2'',3''-dichlorophenyl)-1-[4'-(trifluoromethyl)phenyl]prop-2-en-1-one (A1)

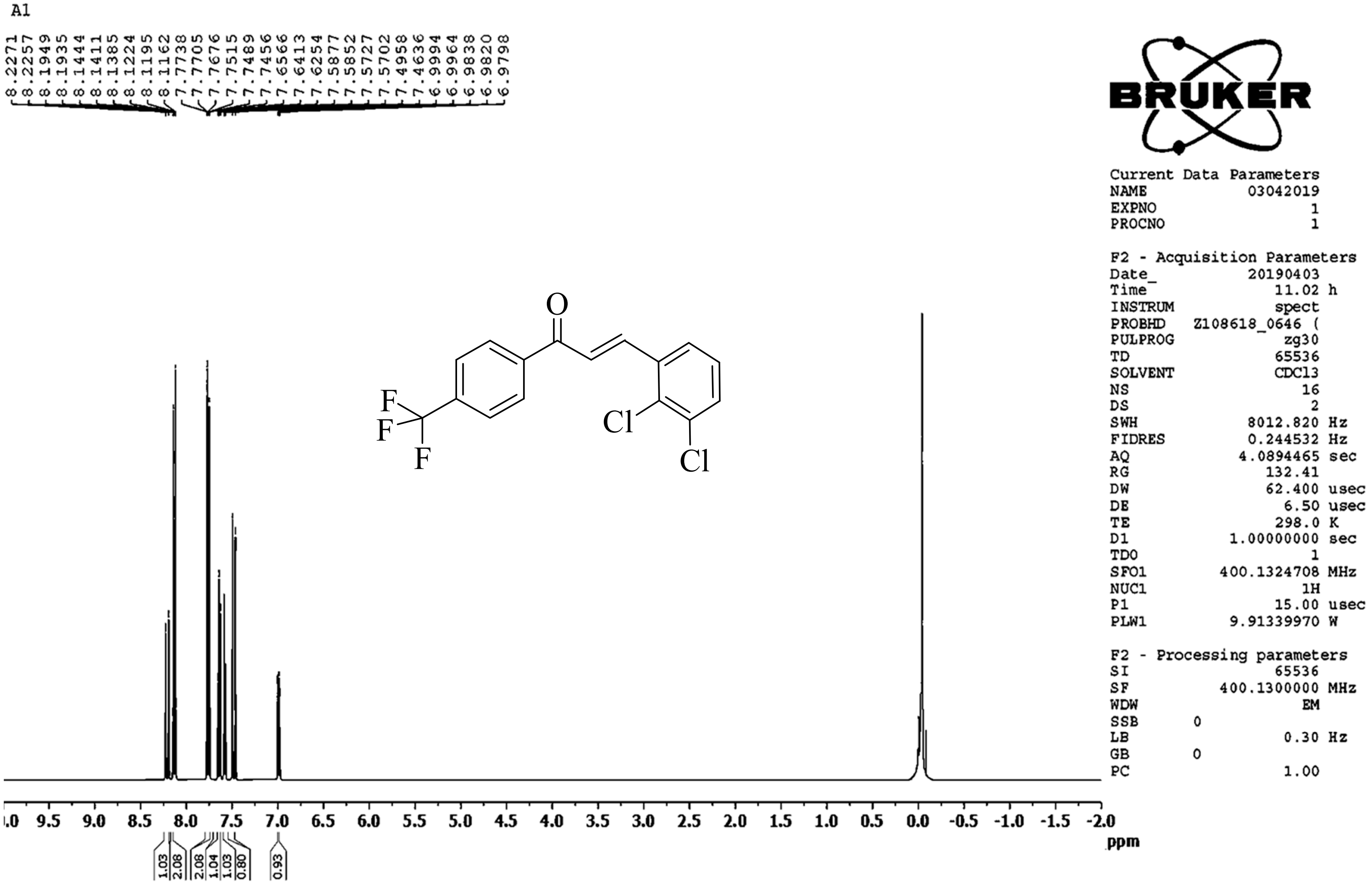

^{1}H NMR Spectrum of *(E)*-3-(2'',3''-dichlorophenyl)-1-[4'-(trifluoromethyl)phenyl]prop-2-en-1-one (A1)

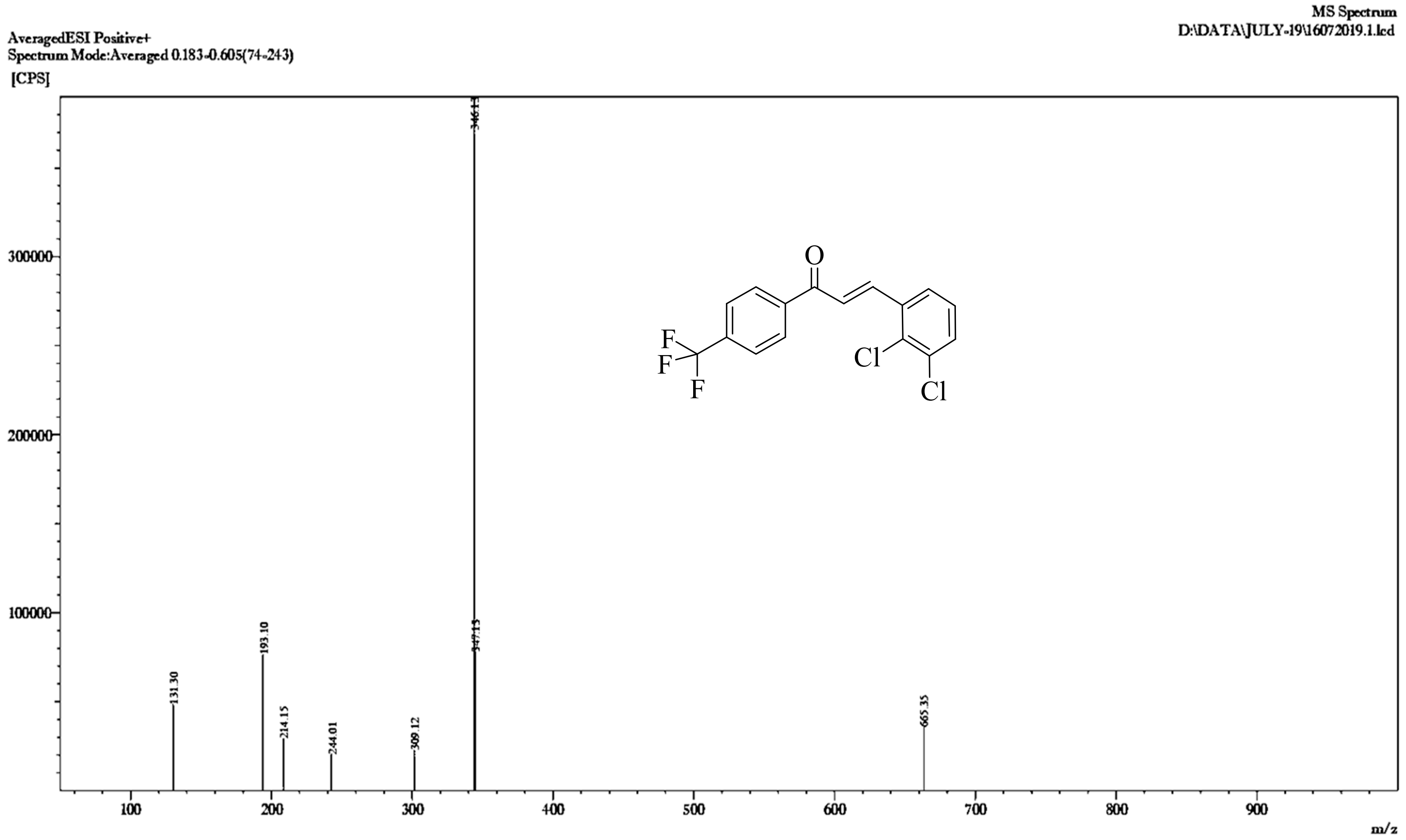

Mass Spectrum of *(E)*-3-(2'',3''-dichlorophenyl)-1-[4'-(trifluoromethyl)phenyl]prop-2-en-1-one (A1)

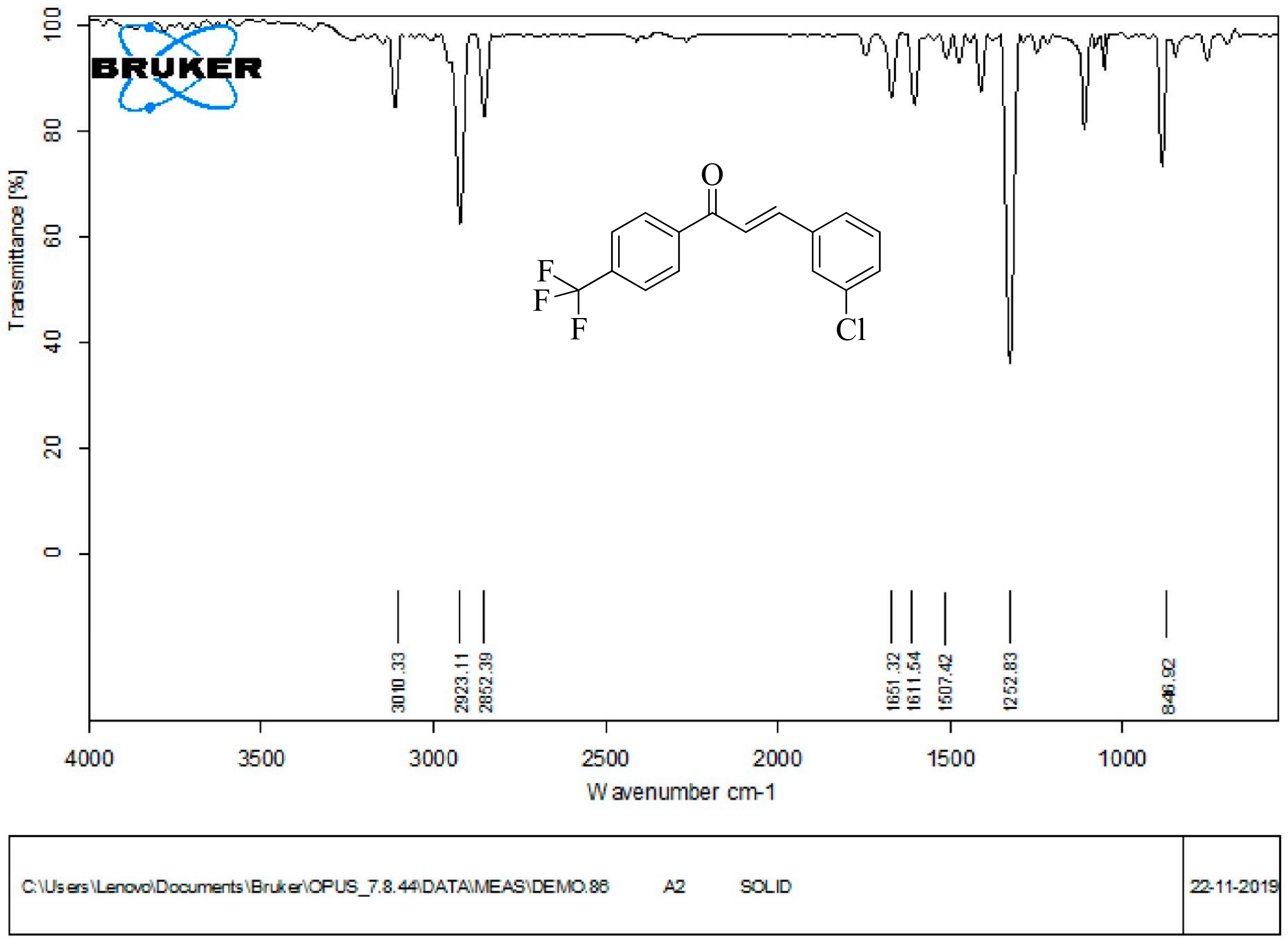

FT-IR Spectrum of *(E)*-3-(2''-chlorophenyl)-1-[4'-(trifluoromethyl)phenyl]prop-2-en-1-one (A2)

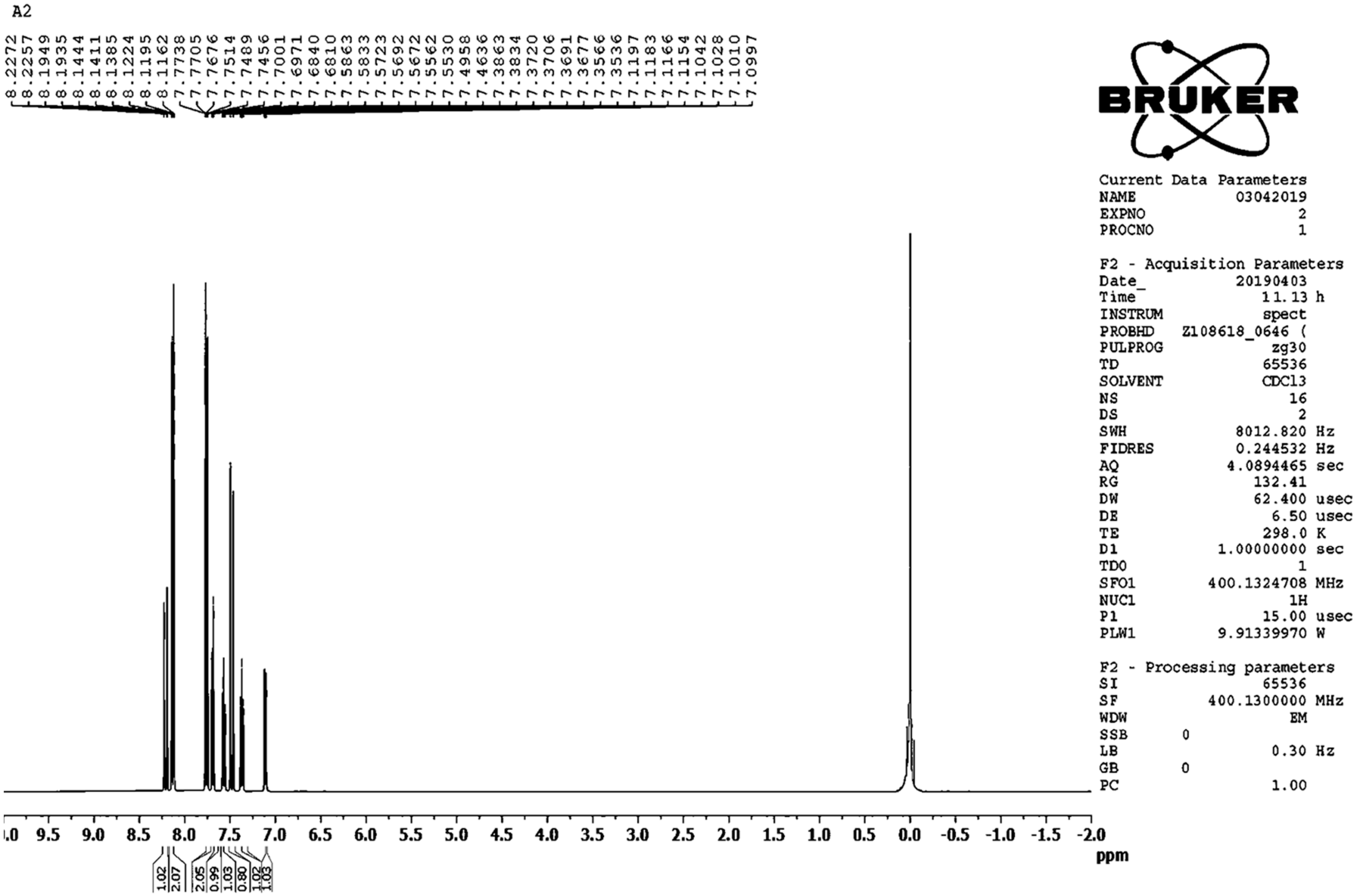

^{1}H NMR Spectrum of *(E)*-3-(3''-chlorophenyl)-1-[4'-(trifluoromethyl)phenyl]prop-2-en-1-one (A2)

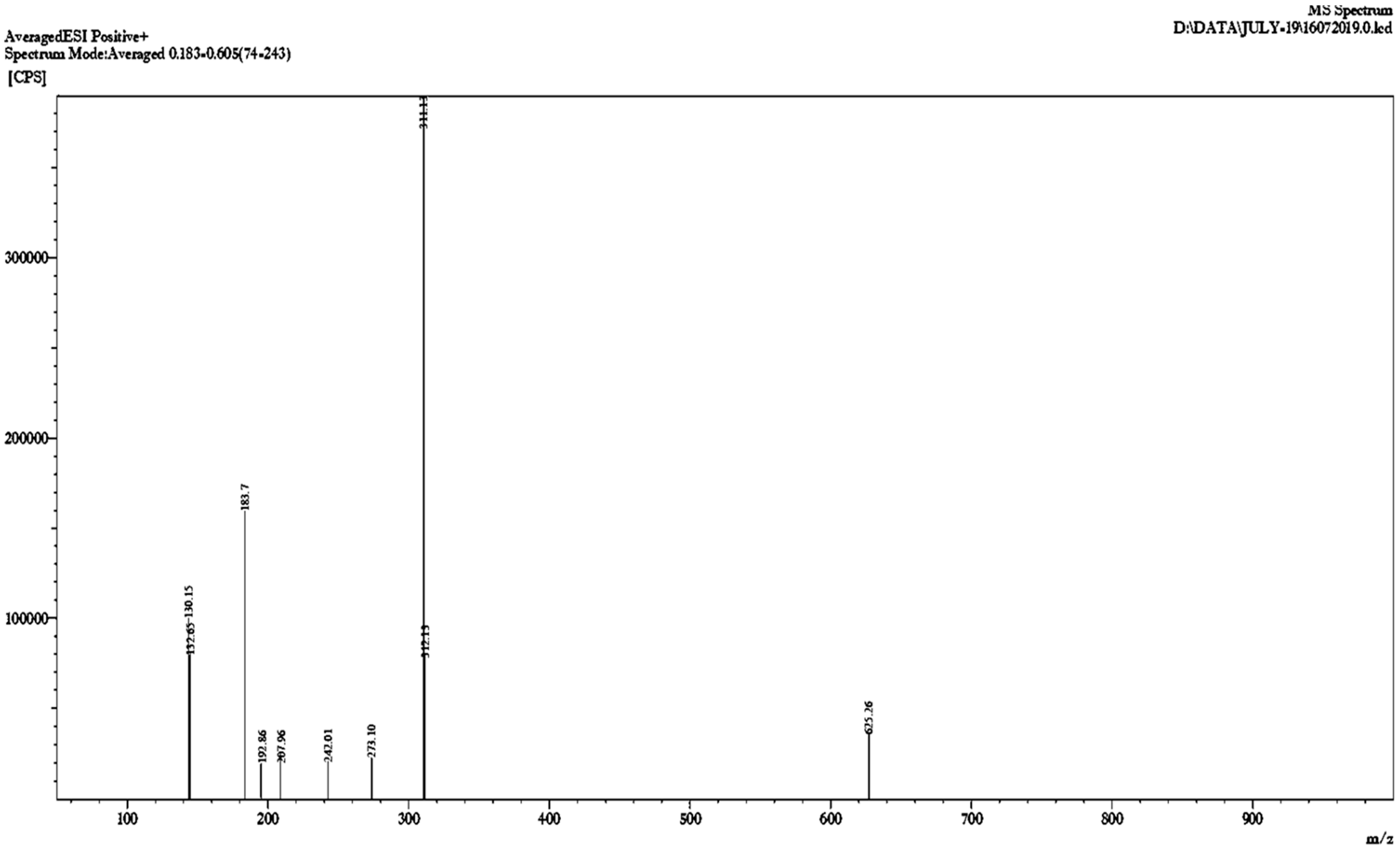

Mass Spectrum of *(E)*-3-(2''-chlorophenyl)-1-[4'-(trifluoromethyl)phenyl]prop-2-en-1-one (A2)

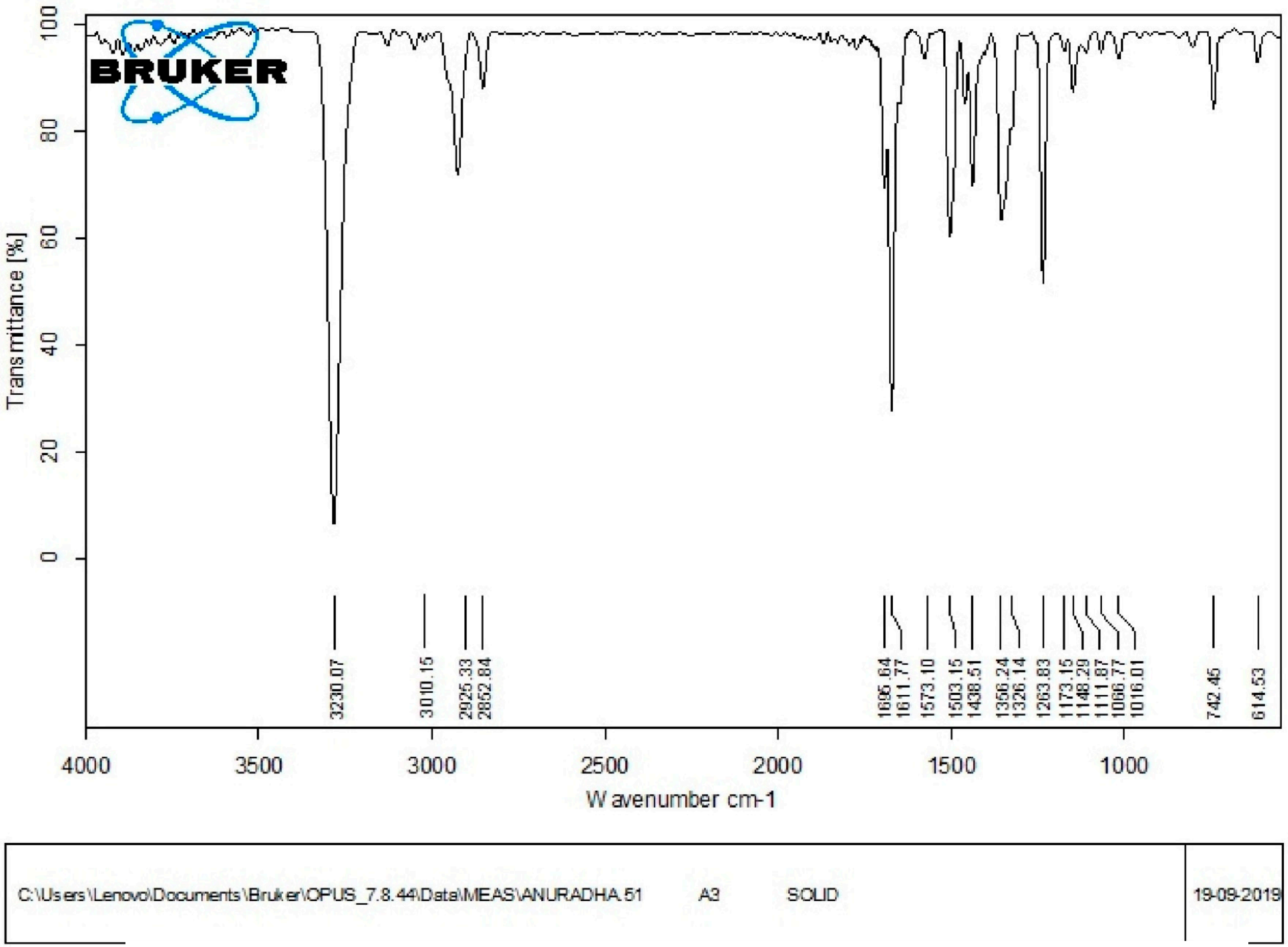

FT-IR Spectrum of (*E*)-3-(1''H-indol-3''-yl)-1-[4'-(trifluoromethyl)phenyl]prop-2-en-1-one (A3)

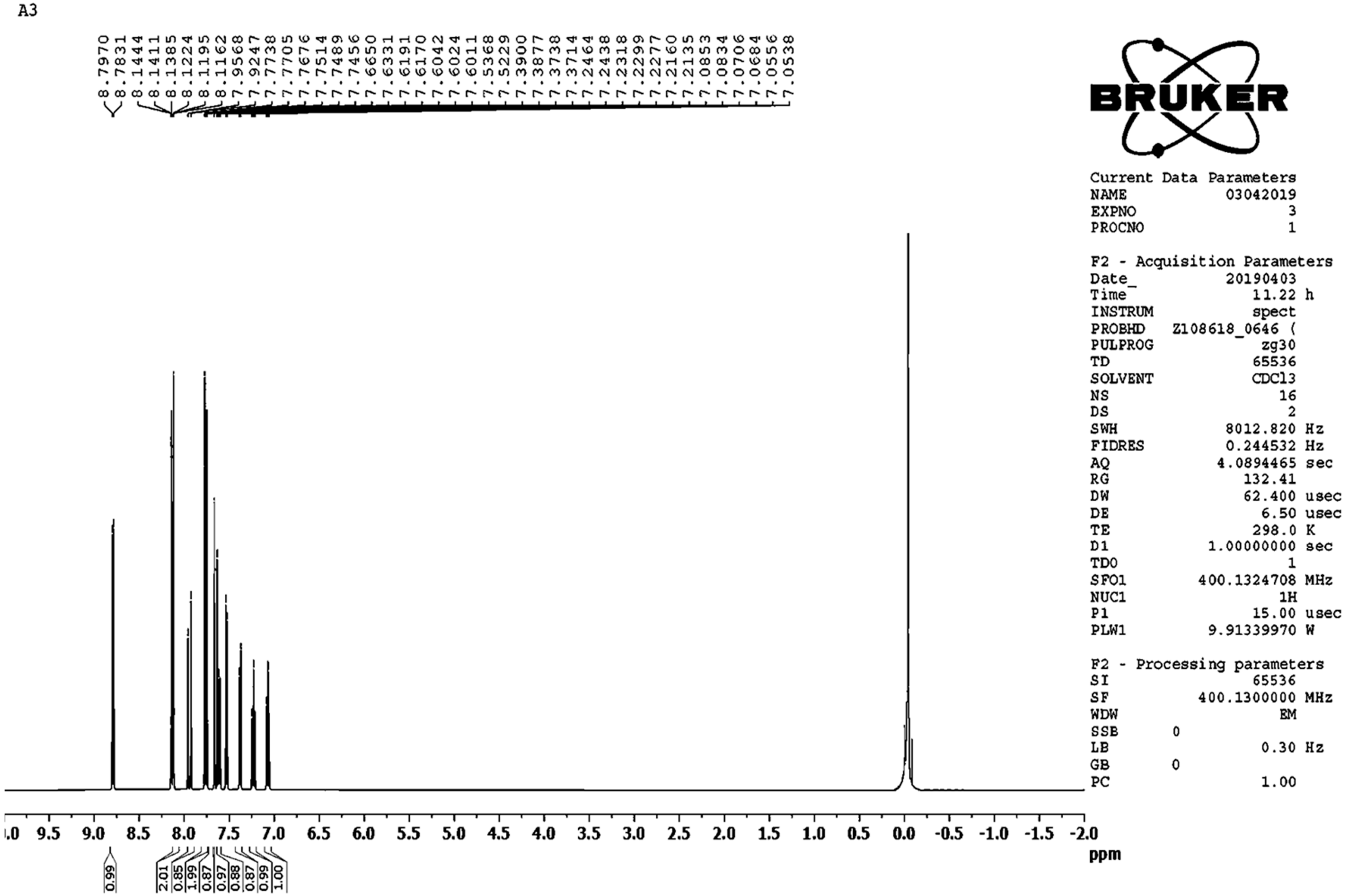

BRUKER

Current Data Parameters
NAME 03042019
EXPNO 3
PROCNO 1

F2 - Acquisition Parameters
Date_ 20190403
Time 11.22 h
INSTRUM spect
PROBHD Z108618_0646 (
PULPROG zg30
TD 65536
SOLVENT CDCl3
NS 16
DS 2
SWH 8012.820 Hz
FIDRES 0.244532 Hz
AQ 4.0894465 sec
RG 132.41
DW 62.400 usec
DE 6.50 usec
TE 298.0 K
D1 1.00000000 sec
TD0 1
SFO1 400.1324708 MHz
NUC1 1H
P1 15.00 usec
PLW1 9.91339970 W

F2 - Processing parameters
SI 65536
SF 400.1300000 MHz
WDW EM
SSB 0
LB 0.30 Hz
GB 0
PC 1.00

^{1}H NMR Spectrum of (*E*)-3-(1''H-indol-3''-yl)-1-[4'-(trifluoromethyl)phenyl]prop-2-en-1-one (A3)

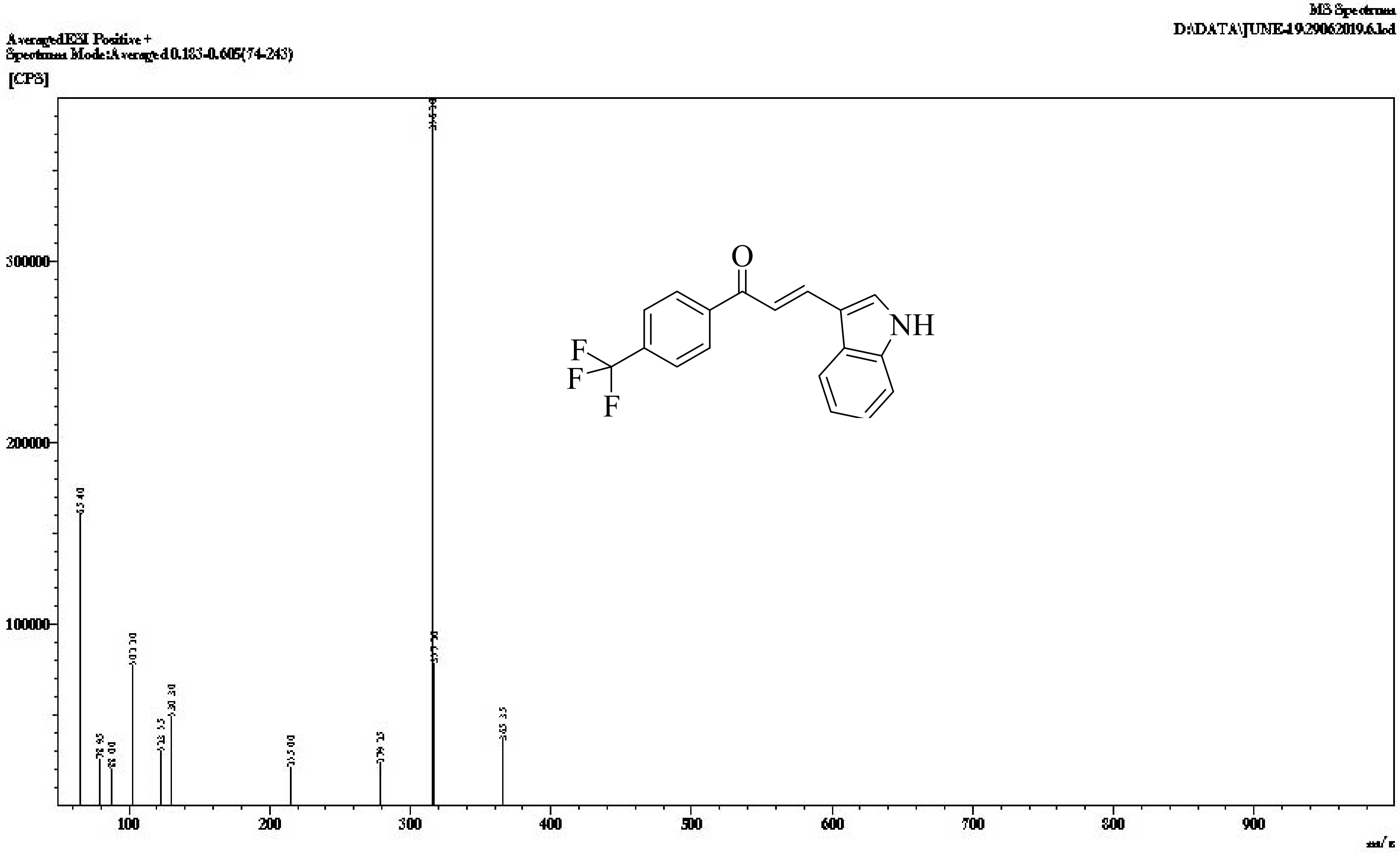

Mass Spectrum of (*E*)-3-(1''H-indol-3''-yl)-1-[4'-(trifluoromethyl)phenyl]prop-2-en-1-one (A3)

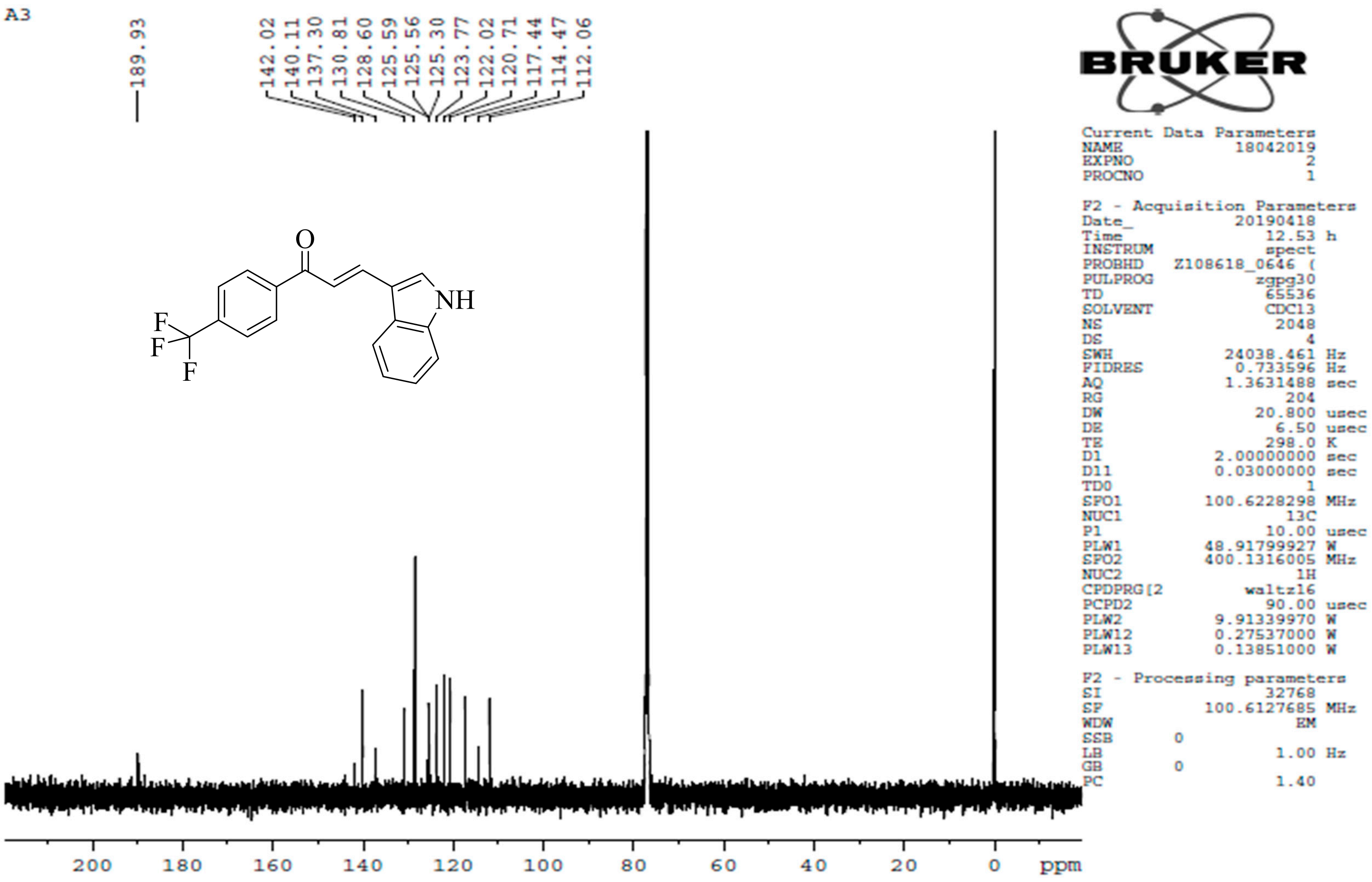

^{13}C NMR Spectrum of (*E*)-3-(1''H-indol-3''-yl)-1-[4'-(trifluoromethyl)phenyl]prop-2-en-1-one (A3)

FA3

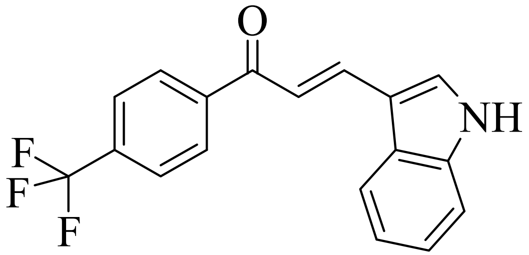

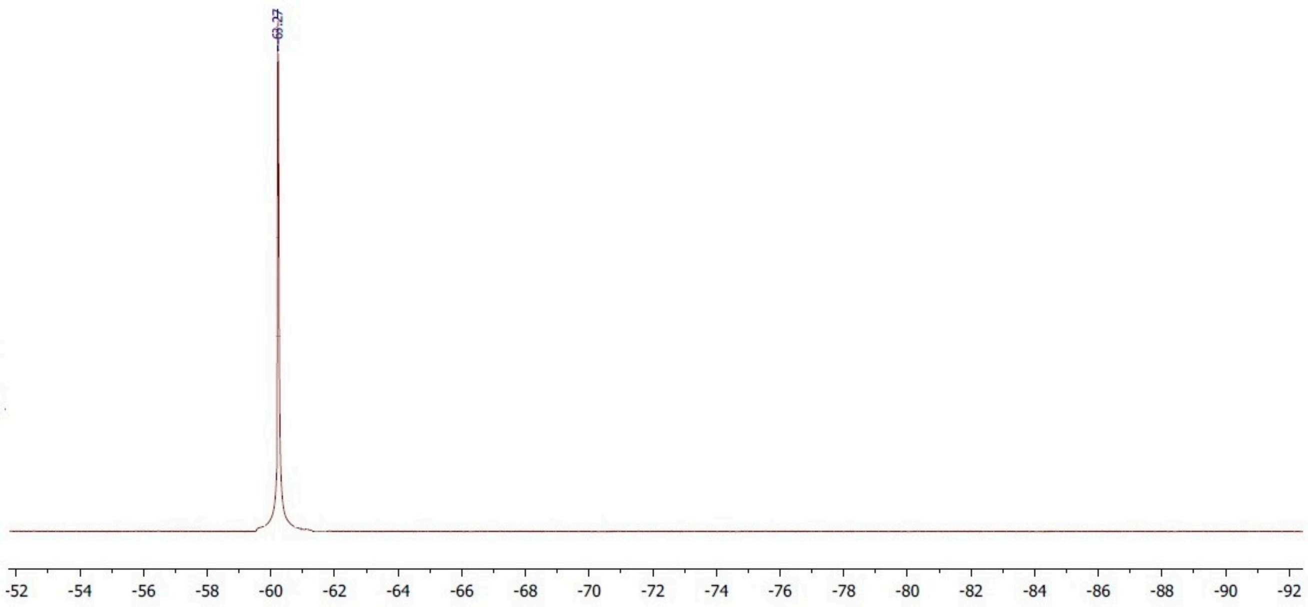

Current Data Parameters
NAME 24042019
EXPNO 1
PROCNO 1

F2 - Acquisition Parameters
Date_ 20190424
Time 12.20 h
INSTRUM spect
PROBHD Z108618_0646 (
PULPROG zg30
TD 65536
SOLVENT CDCl3
NS 16
DS 2
SWH 8012.820 Hz
FIDRES 0.244532 Hz
AQ 4.0894465 sec
RG 132.41
DW 62.400 usec
DE 6.50 usec
TE 298.0 K
D1 1.00000000 sec
TD0 1
SFO1 376.1524708 MHz
NUC1 1H
P1 15.00 usec
PLW1 9.91339970 W

F2 - Processing parameters
SI 65536
SF 376.1500000 MHz
WDW EM
SSB 0
LB 0.30 Hz
GB 0
PC 1.00

[10]F NMR Spectrum of (*E*)-3-(1''H-indol-3''-yl)-1-[4'-(trifluoromethyl)phenyl]prop-2-en-1-one (A3)

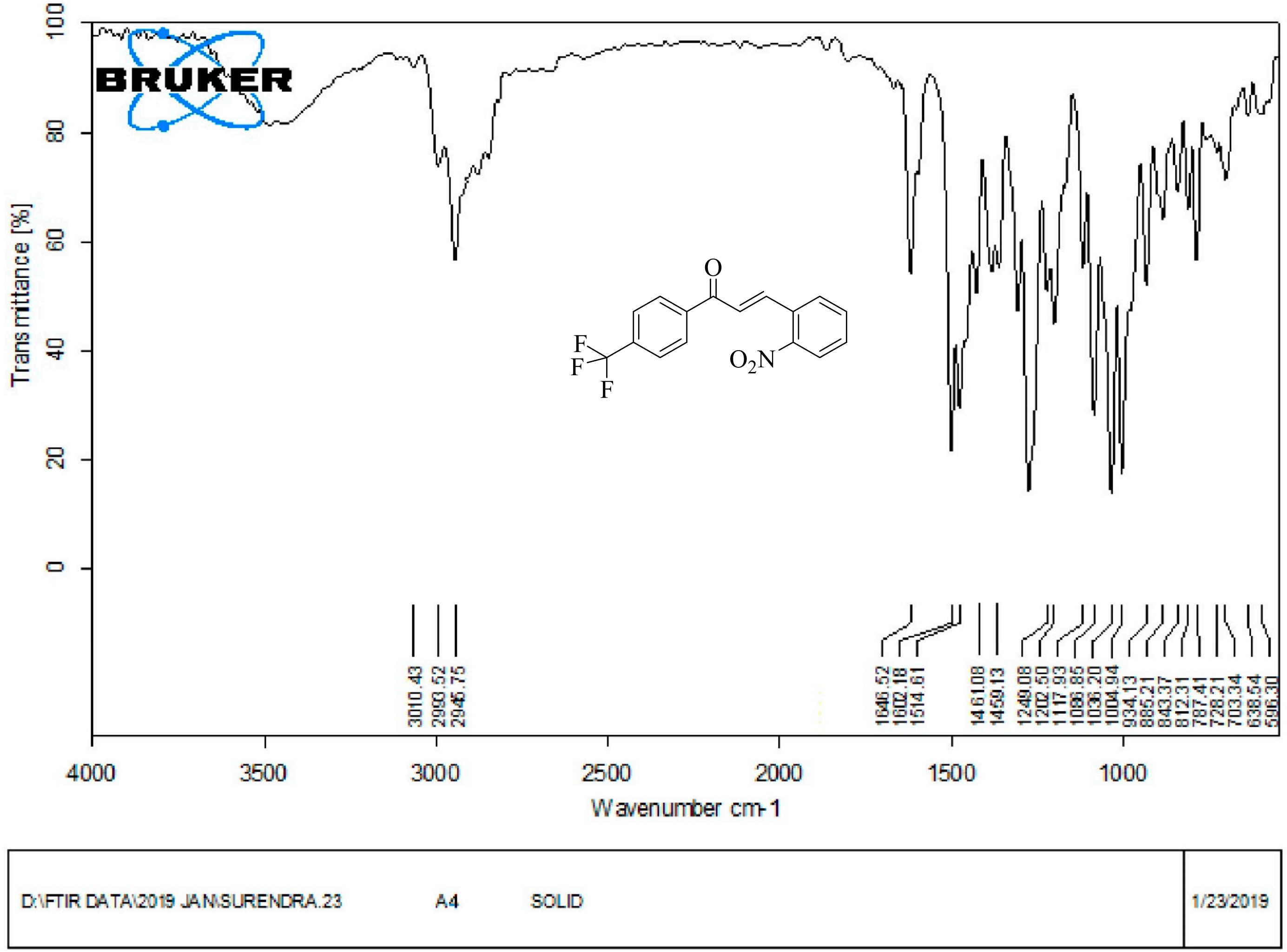

FT-IR Spectrum of (*E*)-3-(2''-nitrophenyl)-1-[4'-(trifluoromethyl)phenyl]prop-2-en-1-one (A4)

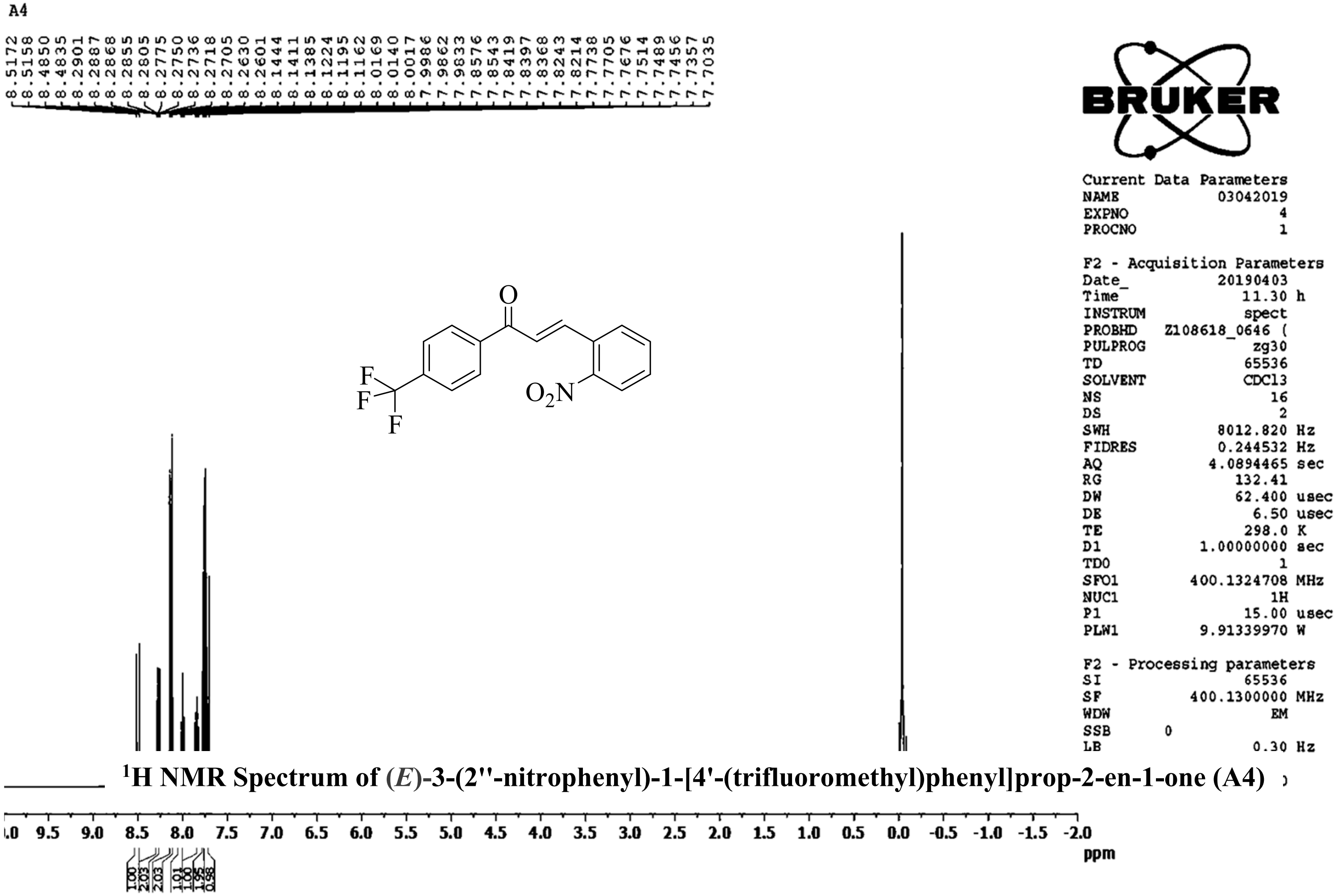

^{1}H NMR Spectrum of (*E*)-3-(2''-nitrophenyl)-1-[4'-(trifluoromethyl)phenyl]prop-2-en-1-one (A4)

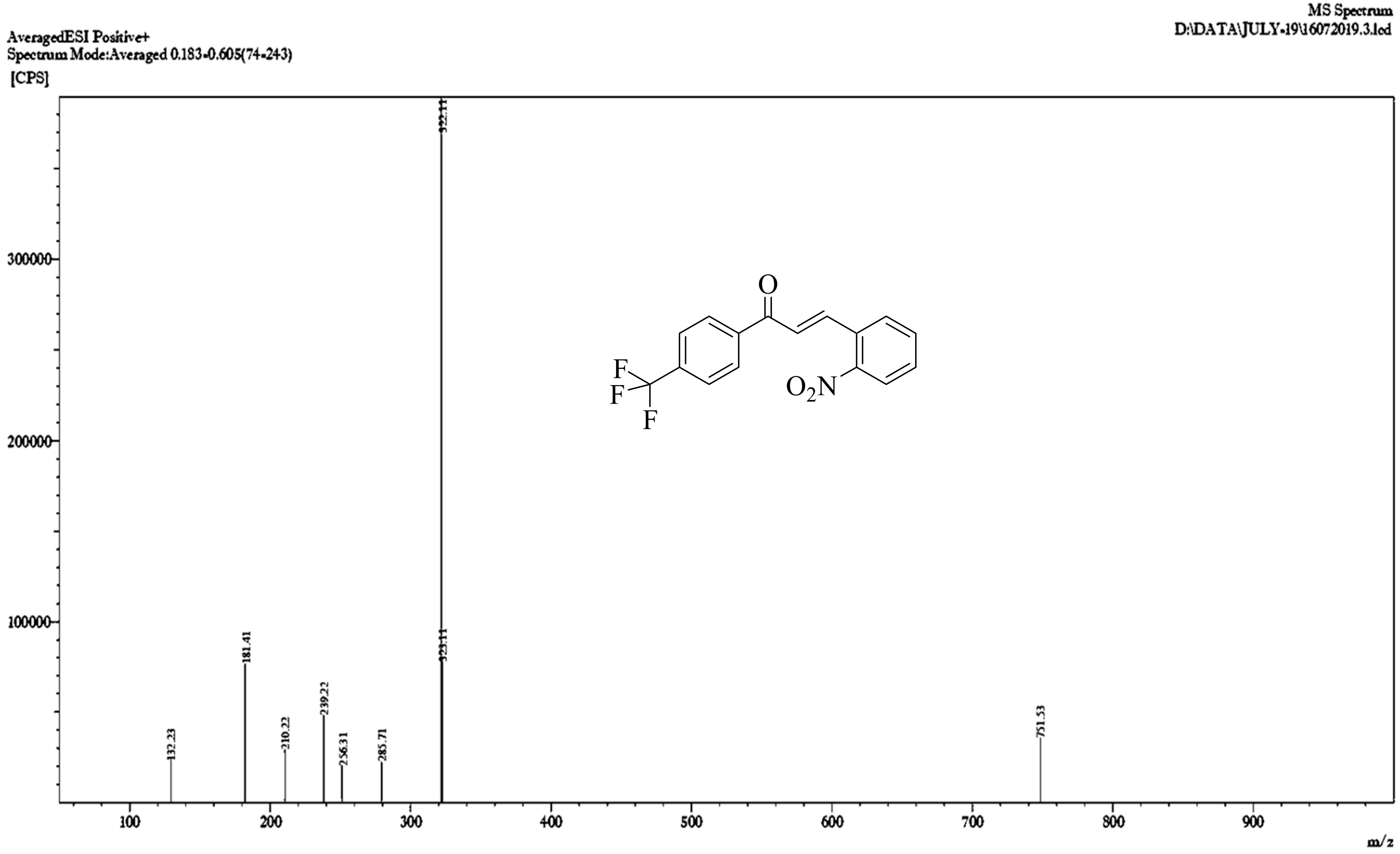

Mass Spectrum of (*E*)-3-(2''-nitrophenyl)-1-[4'-(trifluoromethyl)phenyl]prop-2-en-1-one (A4)

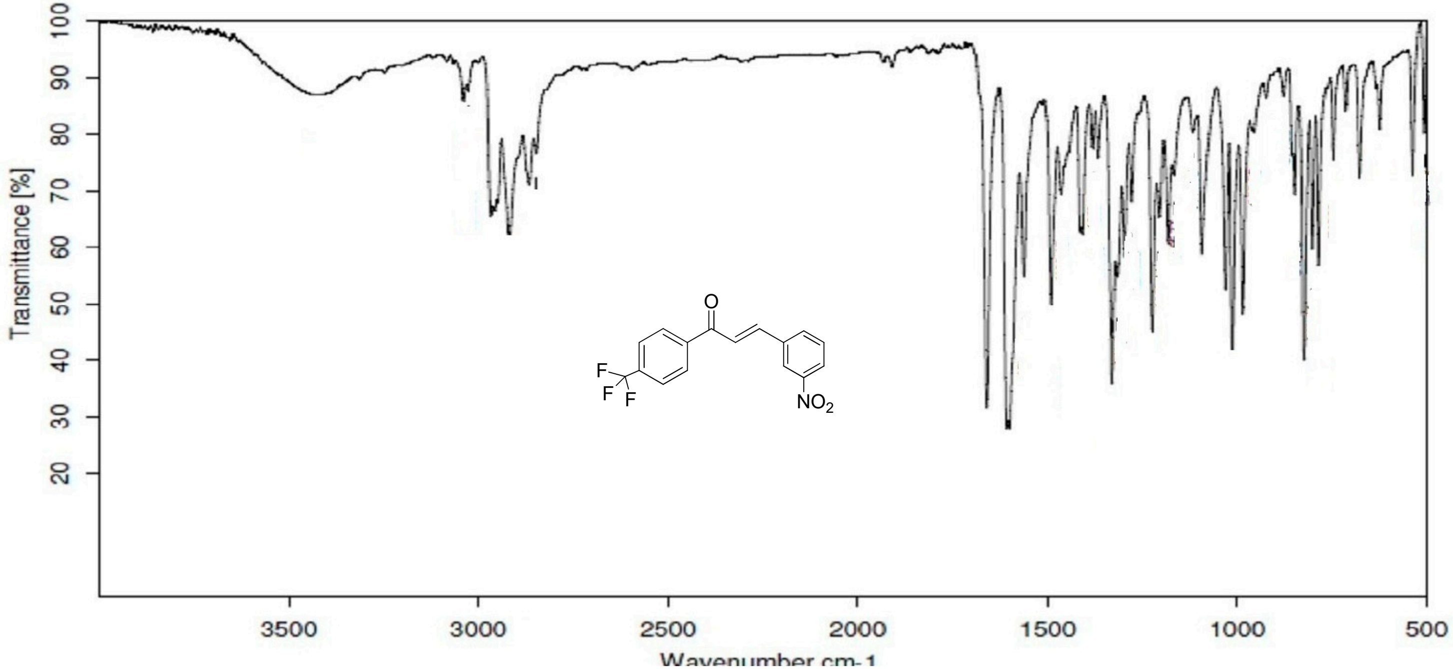

FT-IR Spectrum of (*E*)-3-(3''-nitrophenyl)-1-[4'-(trifluoromethyl)phenyl]prop-2-en-1-one (A5)

A5

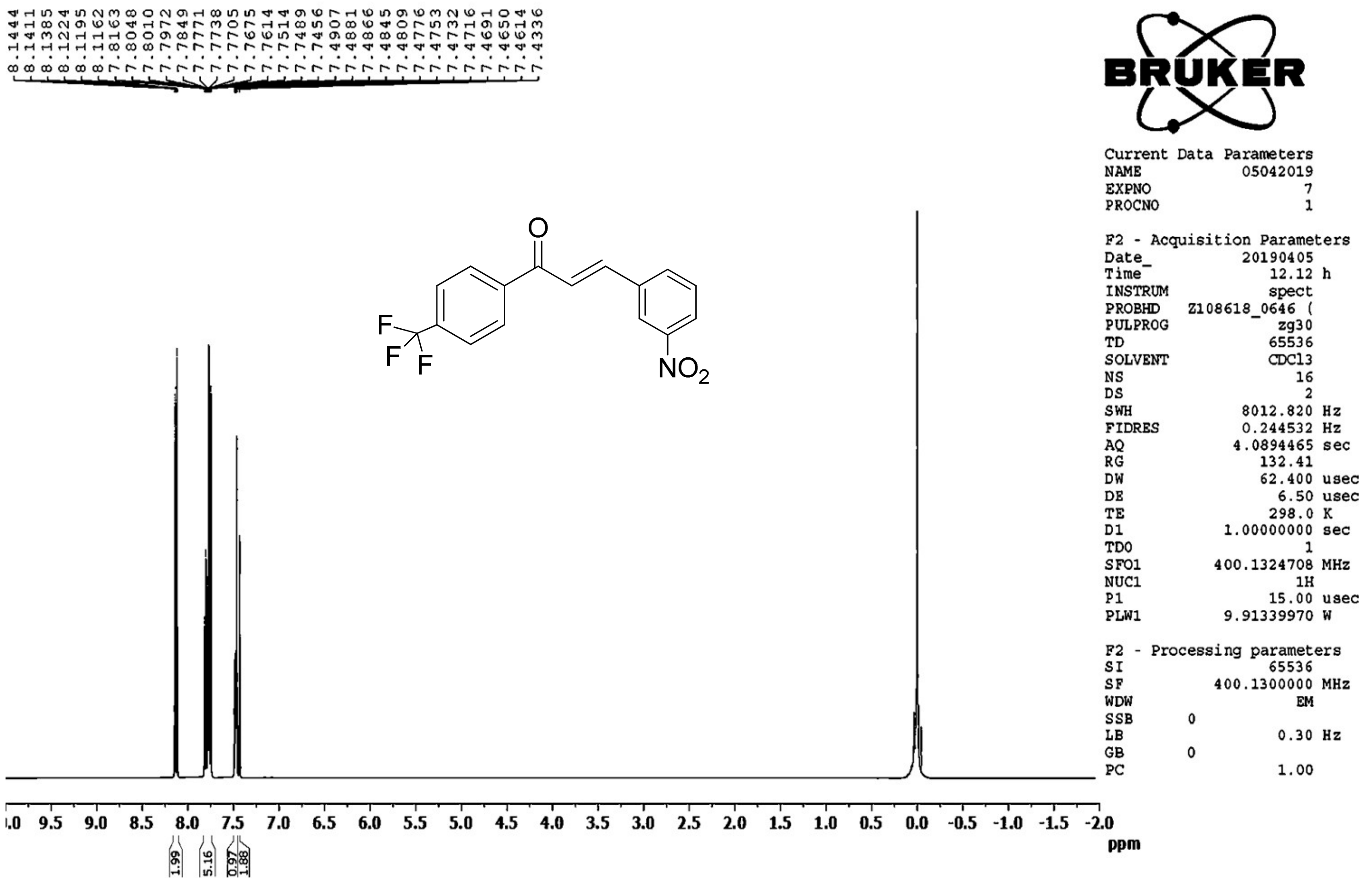

1H NMR Spectrum of (*E*)-3-(3''-nitrophenyl)-1-[4'-(trifluoromethyl)phenyl]prop-2-en-1-one (A5)

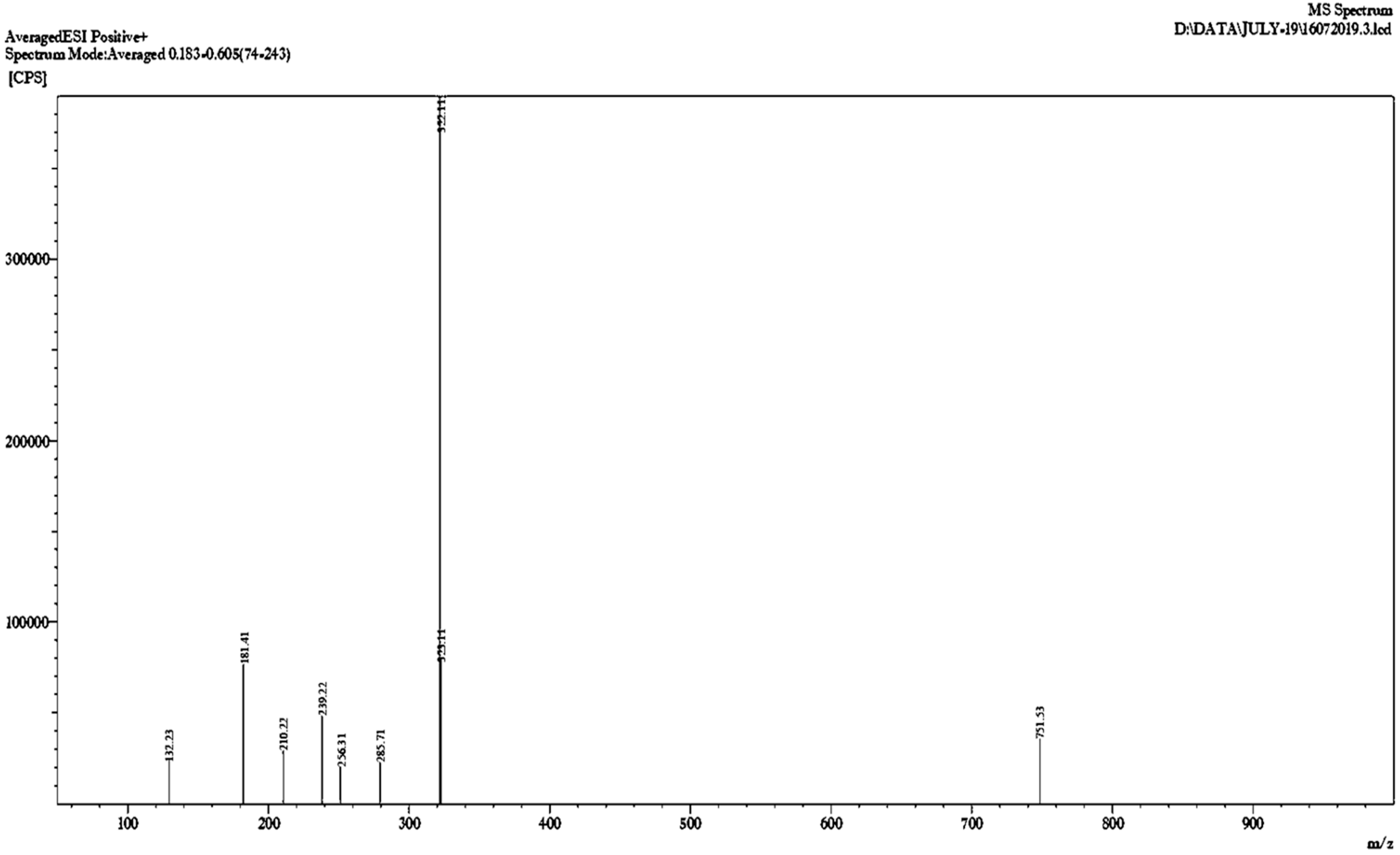

□

Mass Spectrum of (*E*)-3-(3''-nitrophenyl)-1-[4'-(trifluoromethyl)phenyl]prop-2-en-1-one (A5)

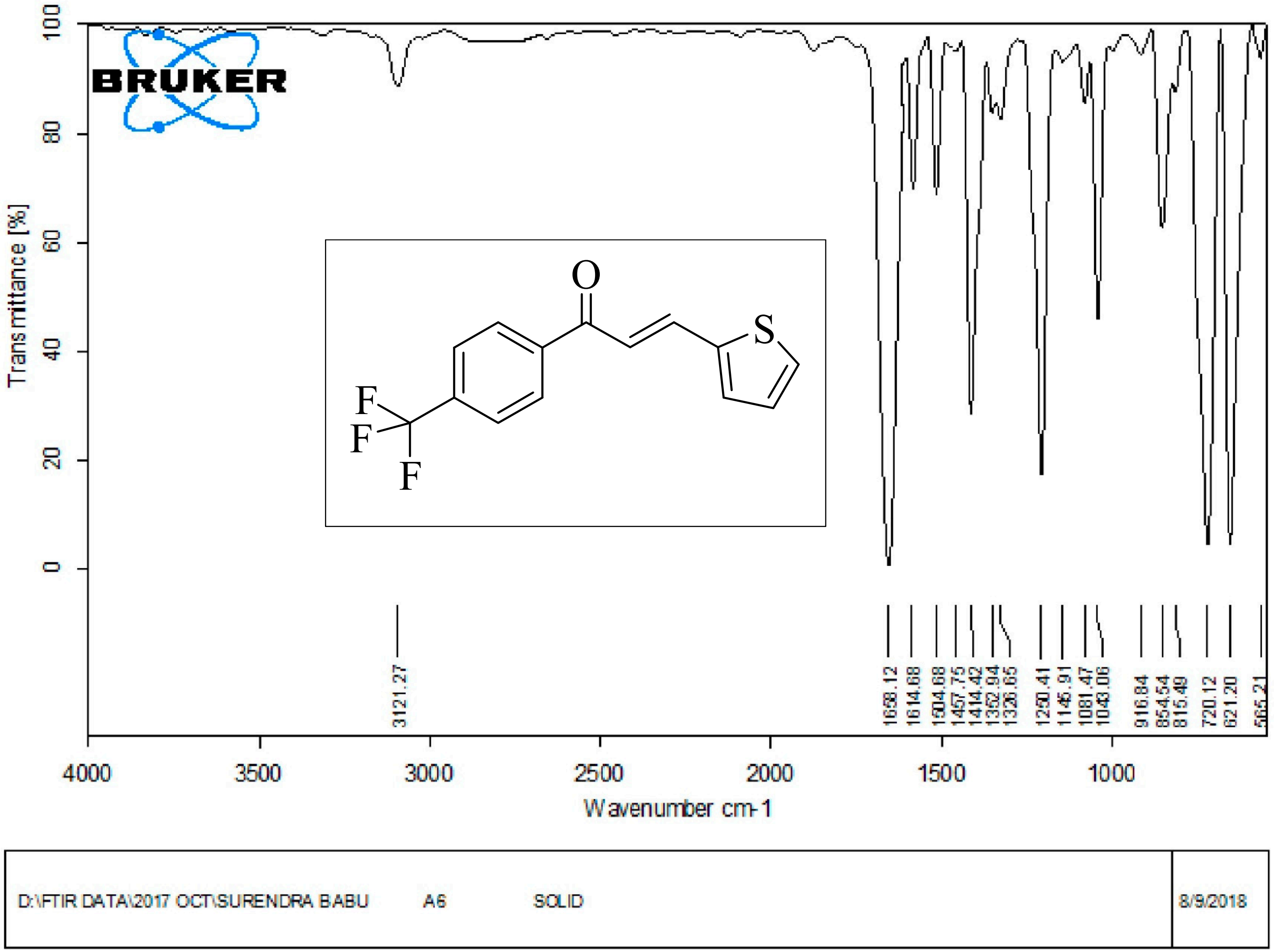

FT-IR Spectrum of *(E)* -3-(thiophen-2''-yl)-1-[4'-(trifluoromethyl)phenyl]prop-2-en-1-one (A6)

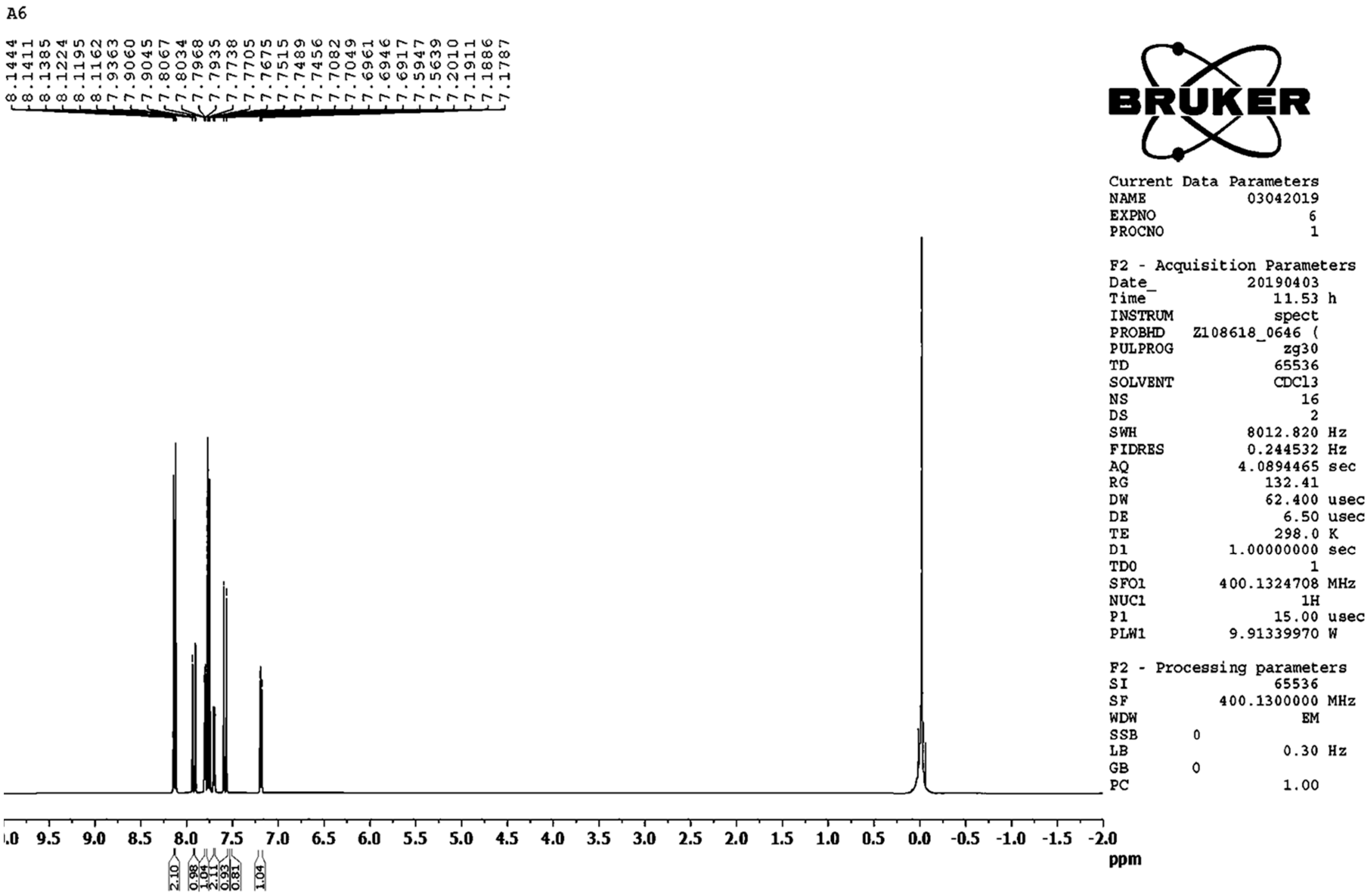

1H NMR Spectrum of *(E)* -3-(thiophen-2''-yl)-1-[4'-(trifluoromethyl)phenyl]prop-2-en-1-one (A6)

Sample Name : KSB-A6-282
Data File : 16072019.5.lcd
Date Acquired : 7/16/2019 10:52:00 AM
Batch File : 16072019.lcb

MS Spectrum
D:\DATA\JULY-19\16072019.5.lcd

AveragedESI Positive+
Spectrum Mode:Averaged 0.183-0.605(74-243)

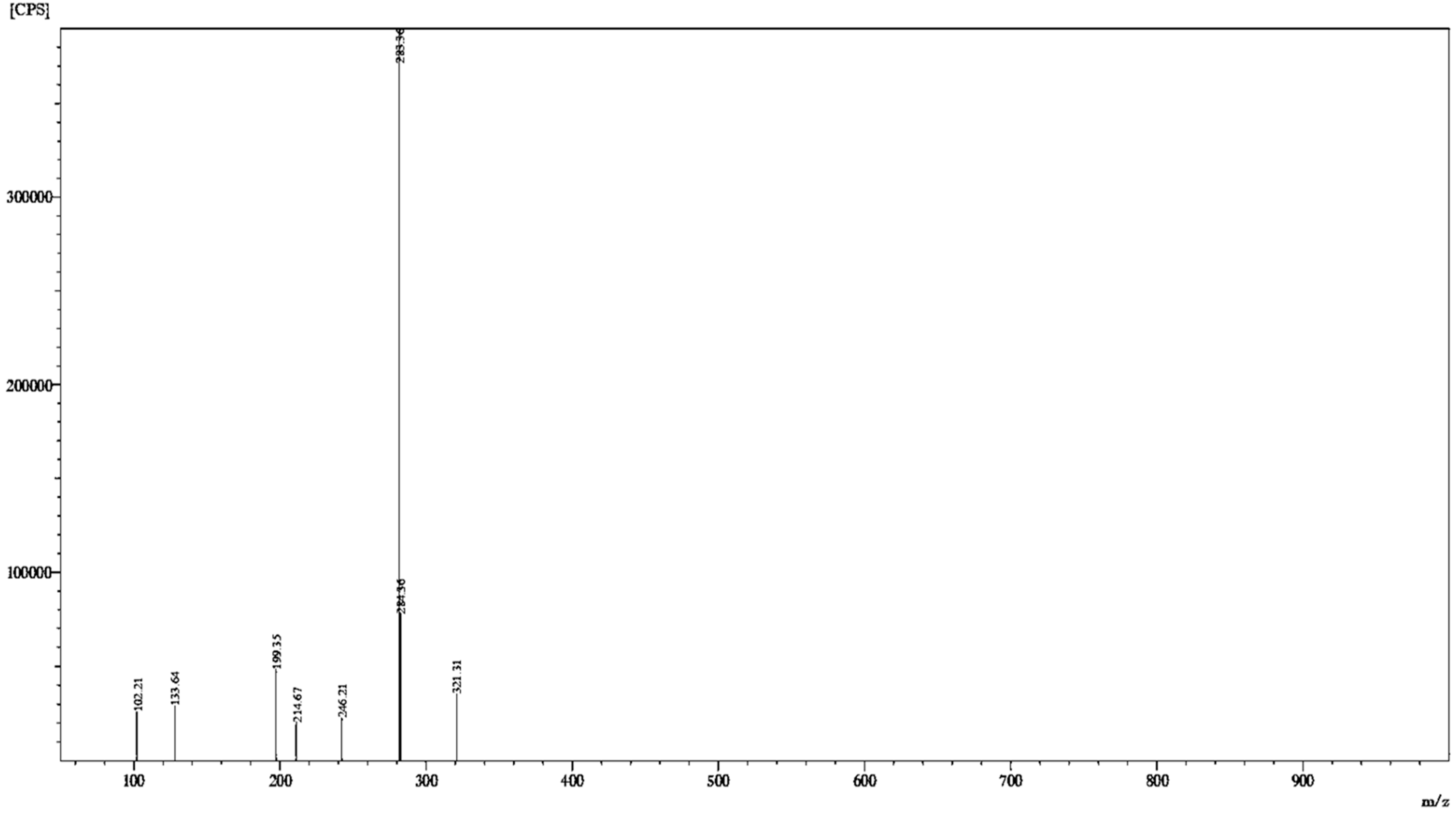

Mass Spectrum of *(E)* -3-(thiophen-2''-yl)-1-[4'-(trifluoromethyl)phenyl]prop-2-en-1-one (A6)

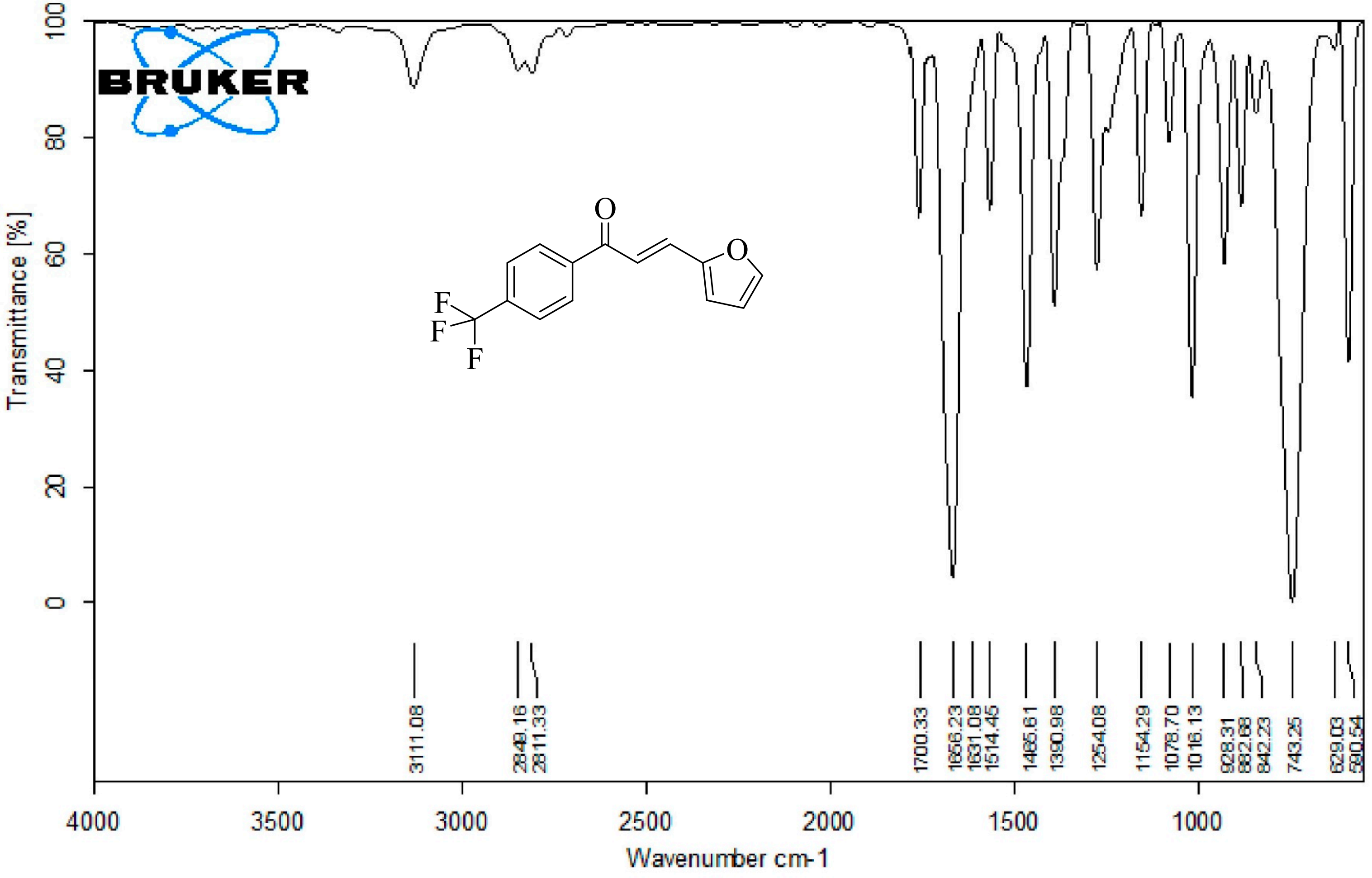

D:\FTIR DATA\2017 OCT\SURENDRA BABU.2 A7 LIQUID	8/9/2018

FT-IR Spectrum of *(E)*-3-(furan-2''-yl)-1-[4'-(trifluoromethyl)phenyl]prop-2-en-1-one (A7)

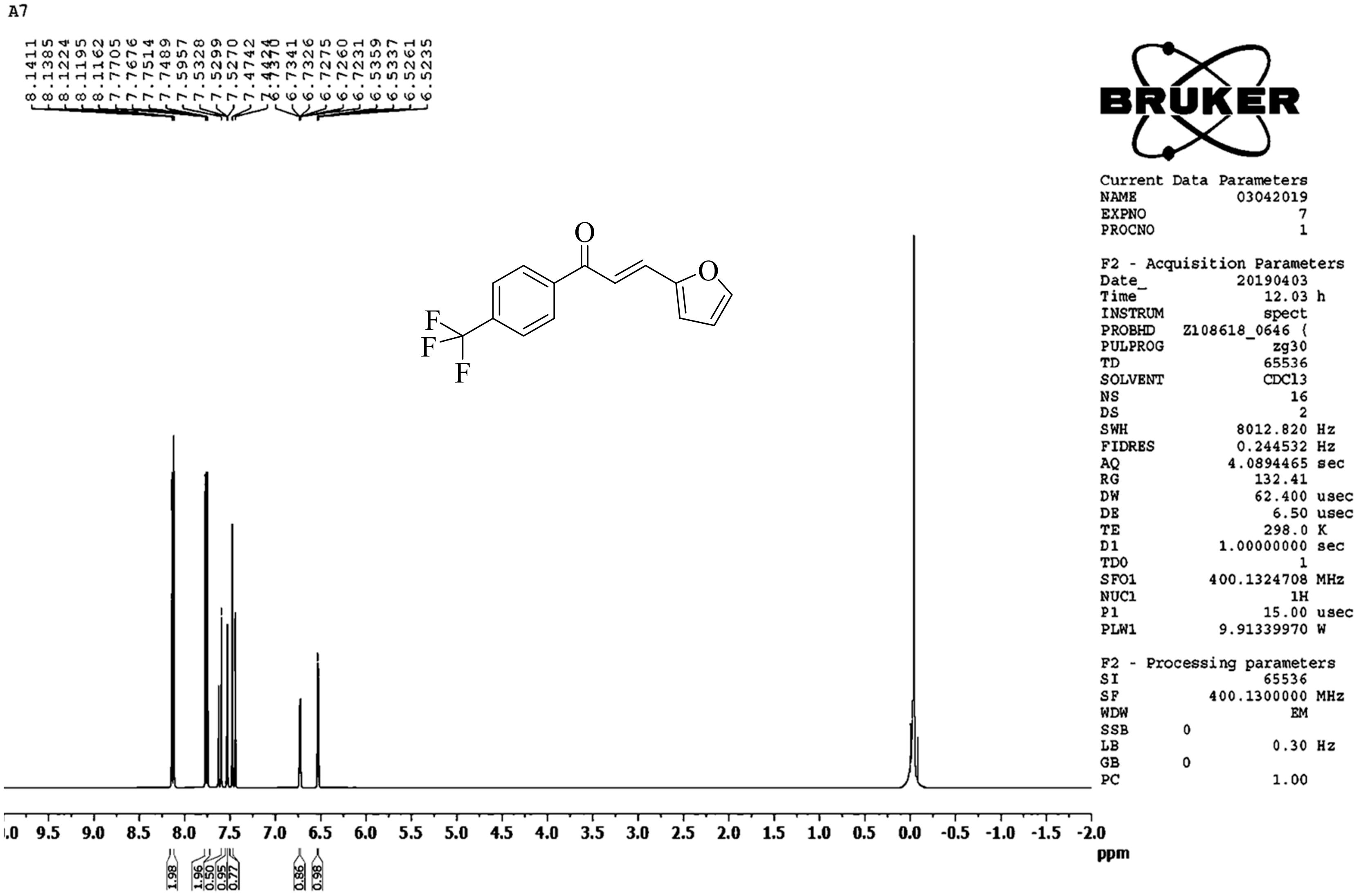

1H NMR Spectrum of *(E)*-3-(furan-2''-yl)-1-[4'-(trifluoromethyl)phenyl]prop-2-en-1-one (A7)

AveragedESI Positive+
Spectrum Mode:Averaged 0.183-0.605(74-243)

MS Spectrum
D:\DATA\JULY-19\16072019.7.lcd

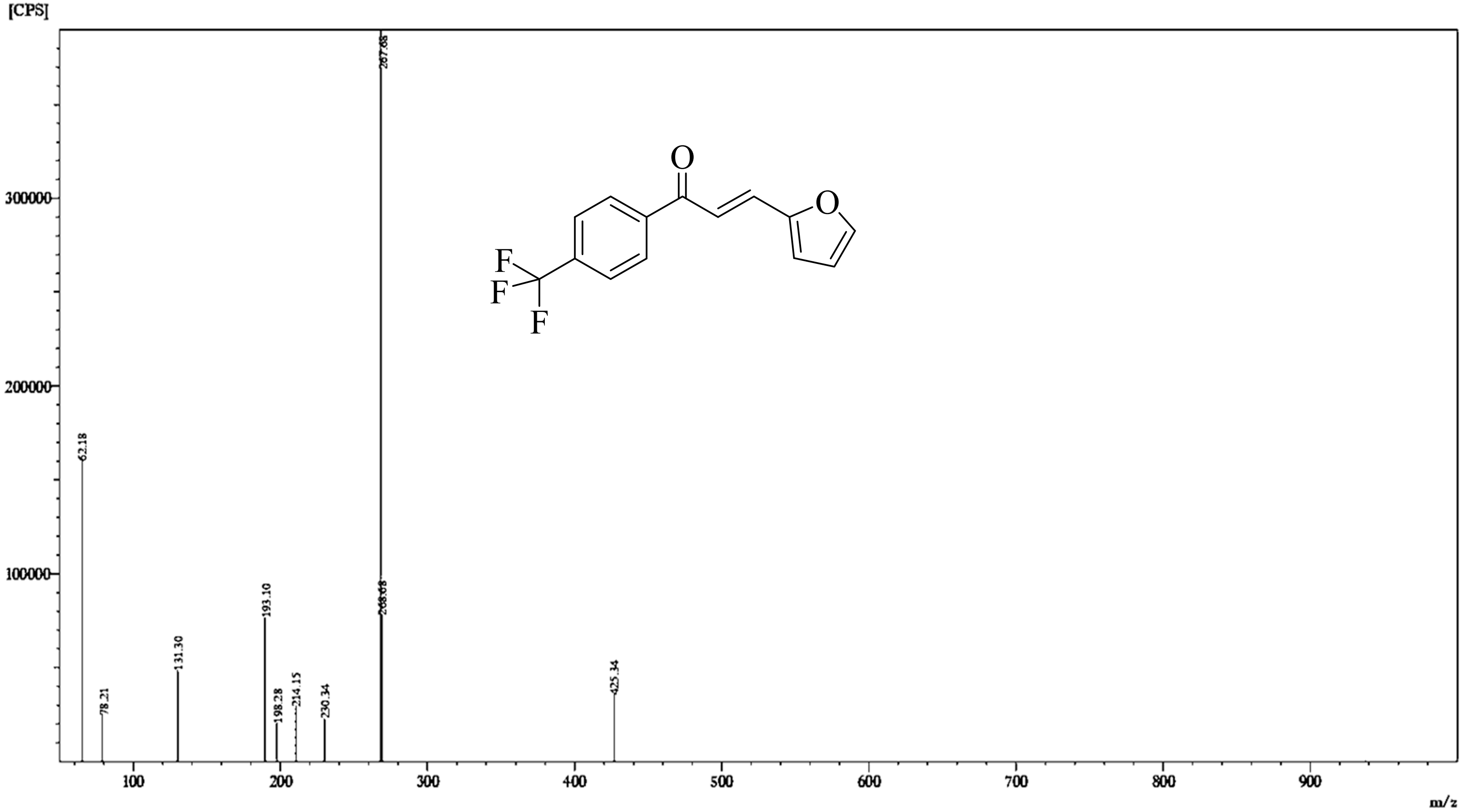

MASS Spectrum of *(E)*-3-(furan-2''-yl)-1-[4'-(trifluoromethyl)phenyl]prop-2-en-1-one (A7)

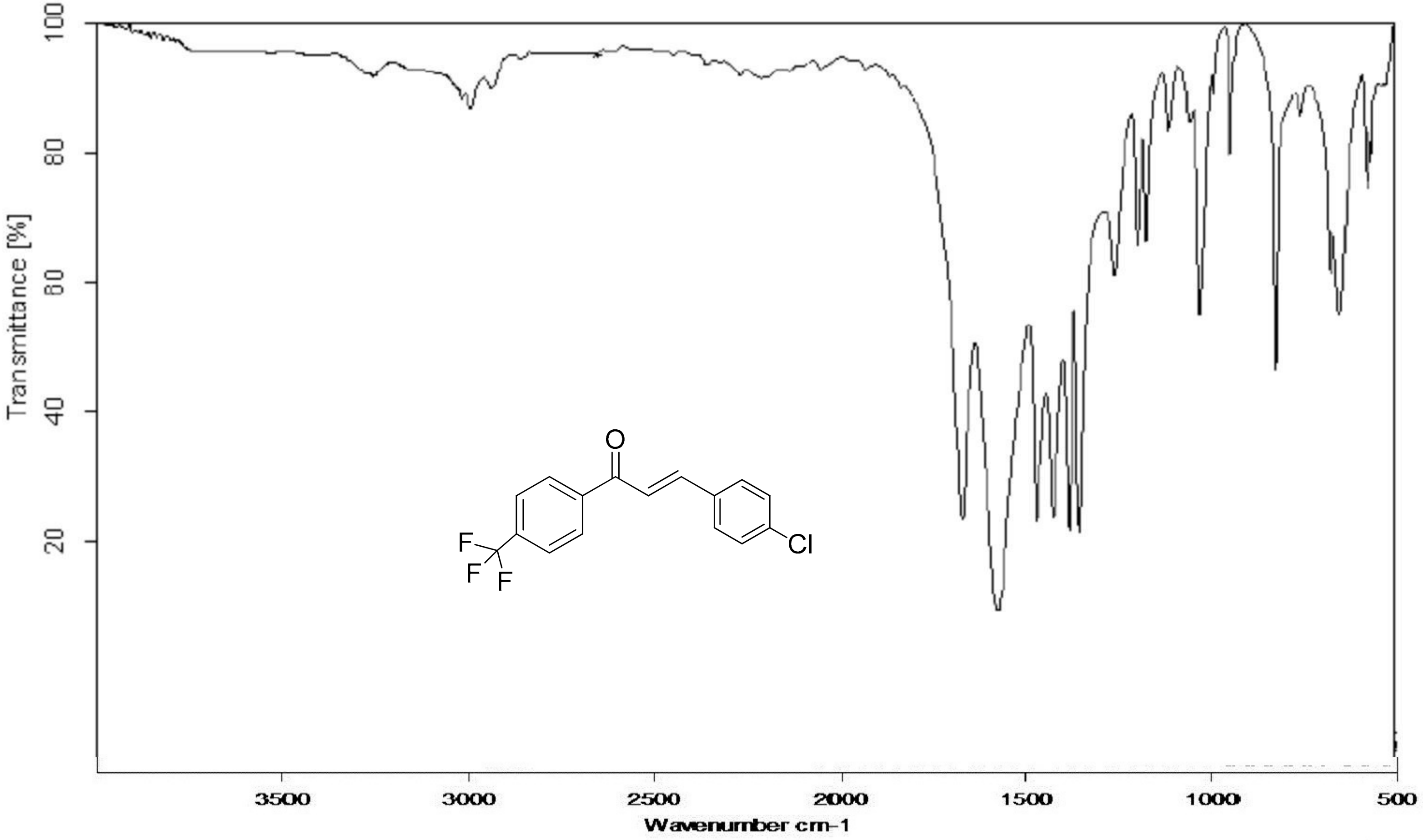

FT-IR Spectrum of *(E)*-3-(4''-chlorophenyl)-1-[4'-(trifluoromethyl)phenyl]prop-2-en-1-one (A8)

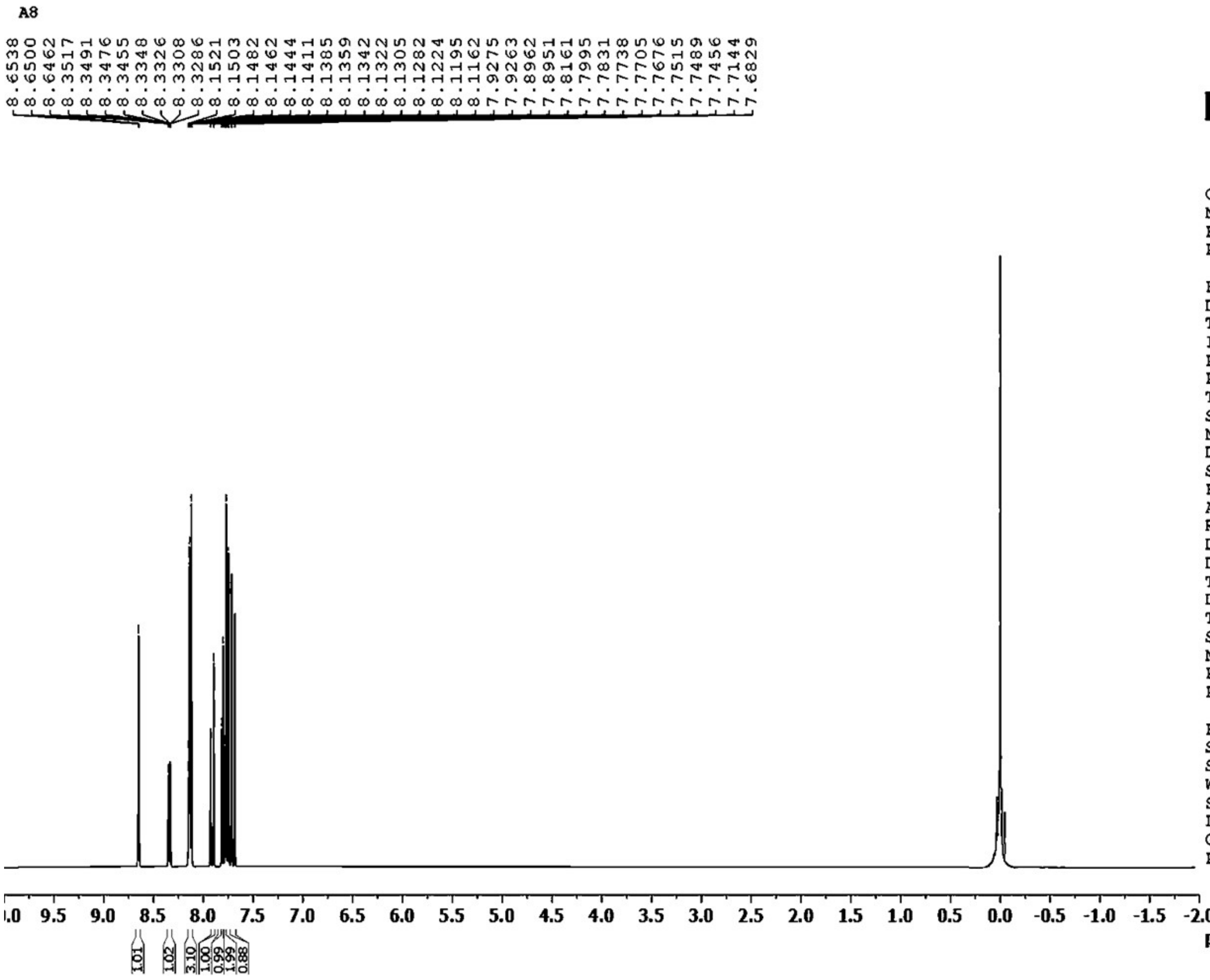

Current Data Parameters
NAME 05042019
EXPNO 1
PROCNO 1

F2 - Acquisition Parameters
Date_ 20190405
Time 12.03 h
INSTRUM spect
PROBHD Z108618_0646 (
PULPROG zg30
TD 65536
SOLVENT CDCl3
NS 16
DS 2
SWH 8012.820 Hz
FIDRES 0.244532 Hz
AQ 4.0894465 sec
RG 132.41
DW 62.400 usec
DE 6.50 usec
TE 298.0 K
D1 1.00000000 sec
TD0 1
SFO1 400.1324708 MHz
NUC1 1H
P1 15.00 usec
PLW1 9.91339970 W

F2 - Processing parameters
SI 65536
SF 400.1300000 MHz
WDW EM
SSB 0
LB 0.30 Hz
GB 0
PC 1.00

1H NMR Spectrum of *(E)*-3-(4''-chlorophenyl)-1-[4'-(trifluoromethyl)phenyl]prop-2-en-1-one (A8)

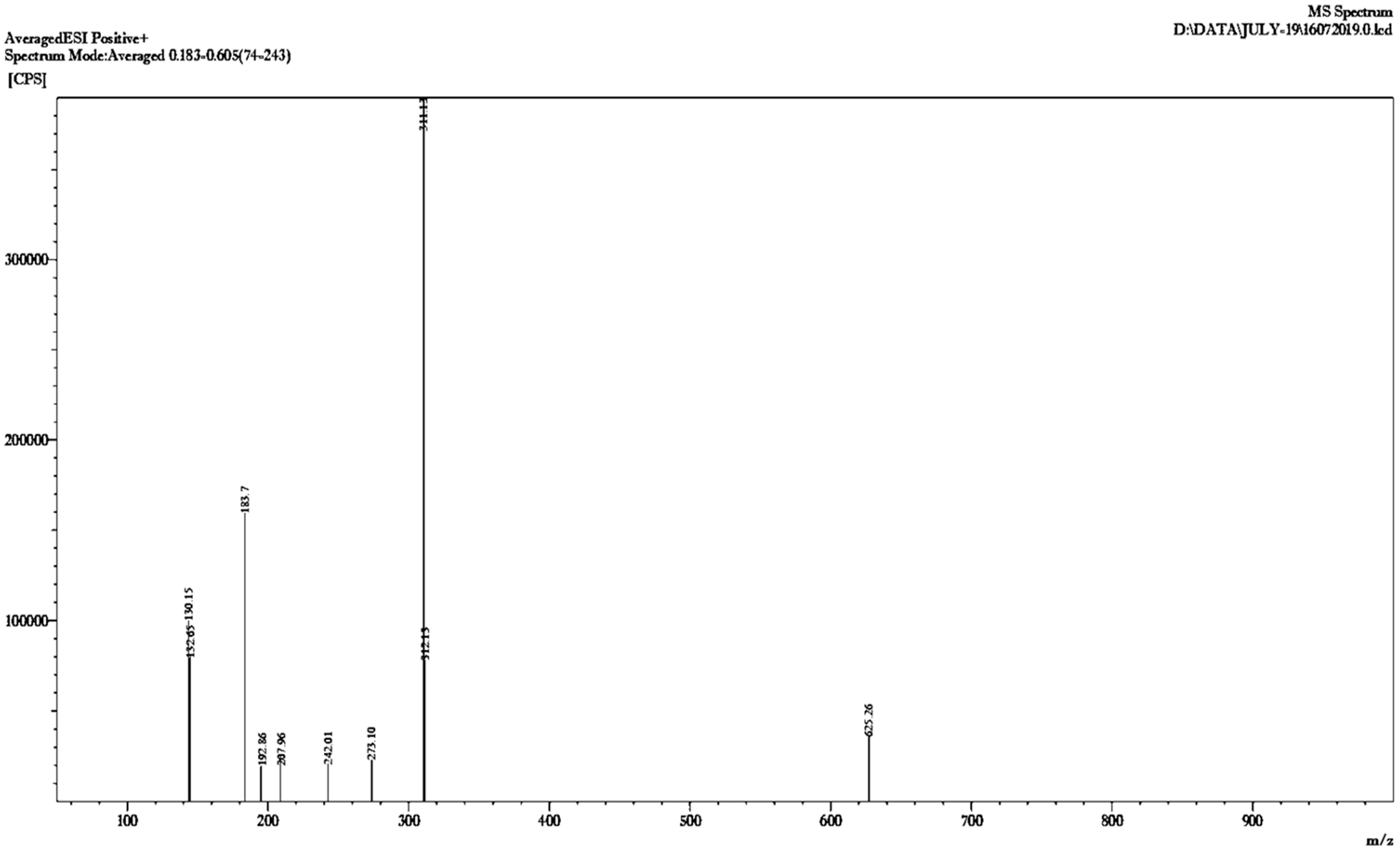

Mass Spectrum of *(E)*-3-(4''-chlorophenyl)-1-[4'-(trifluoromethyl)phenyl]prop-2-en-1-one (A8)

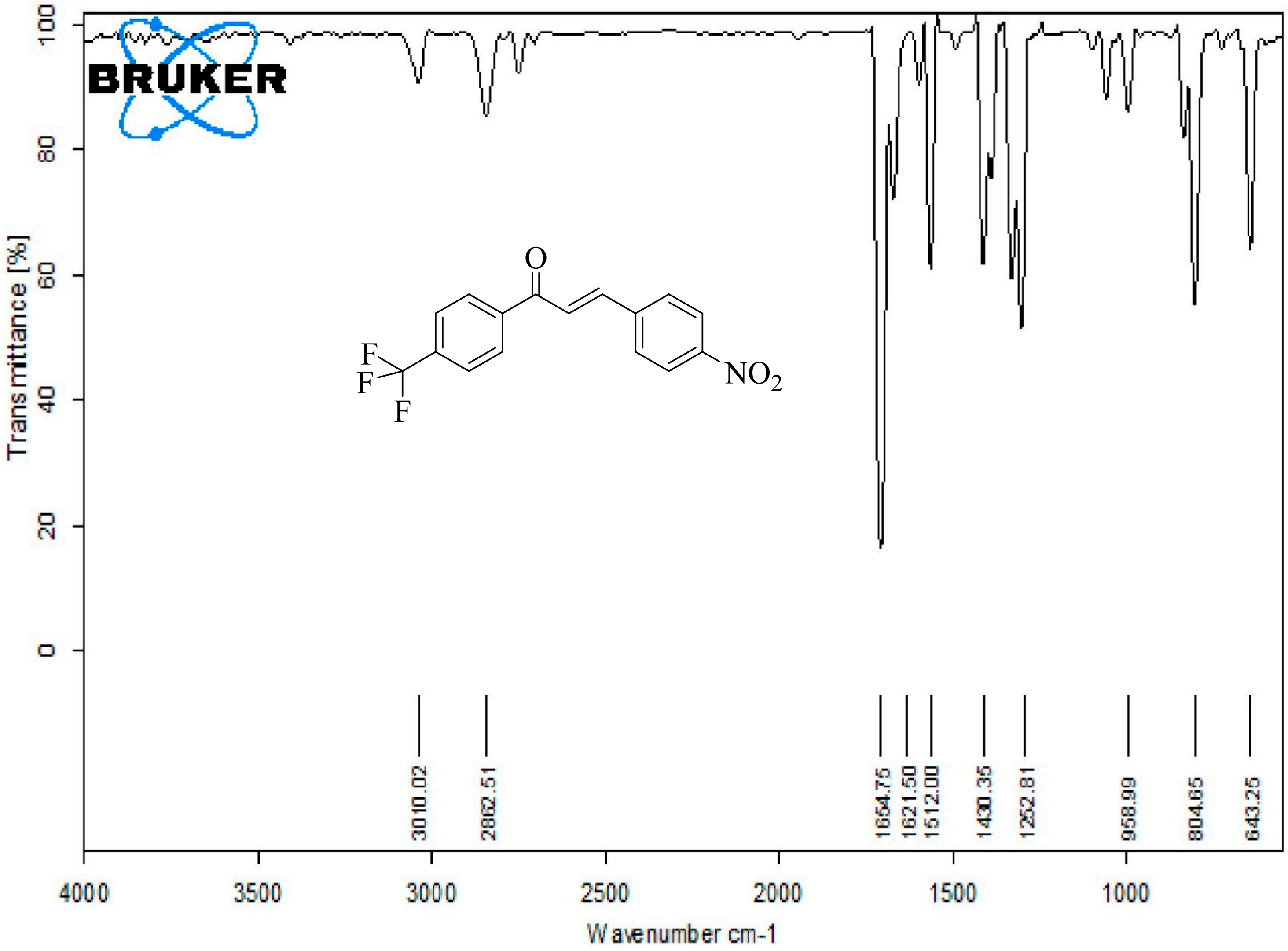

FI-IR Spectrum of 1-(4-(trifluoromethyl)phenyl)-3-(4-nitrophenyl)prop-2-en-1-one (A9)

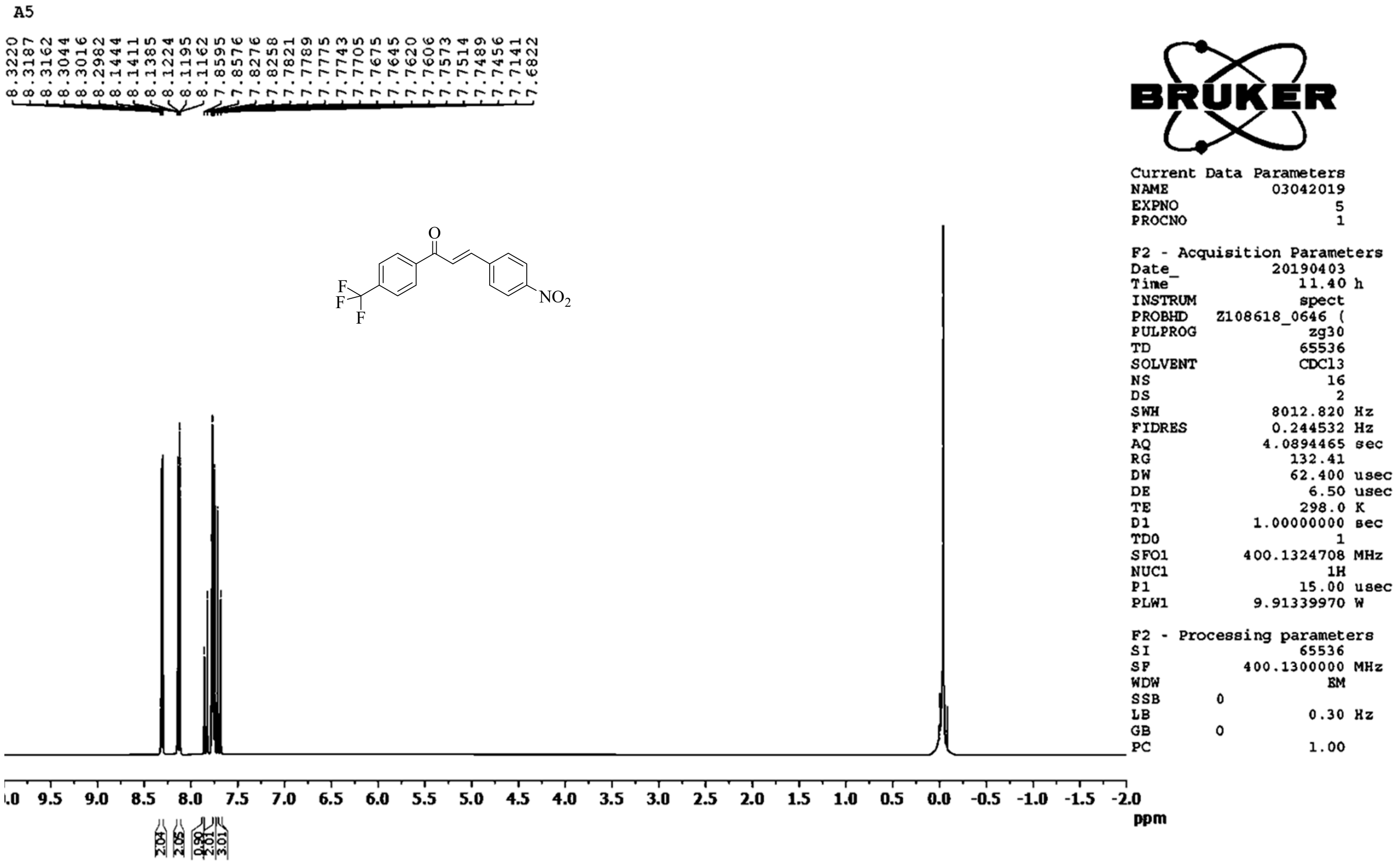

1H NMR Spectrum of 1-(4-(trifluoromethyl)phenyl)-3-(4-nitrophenyl)prop-2-en-1-one (A9)

Sample Name : KSB-A5-345
Data File : 16072019.4.lcd
Date Acquired : 7/16/2019 10:30:43 AM
Batch File : 16072019.lcb

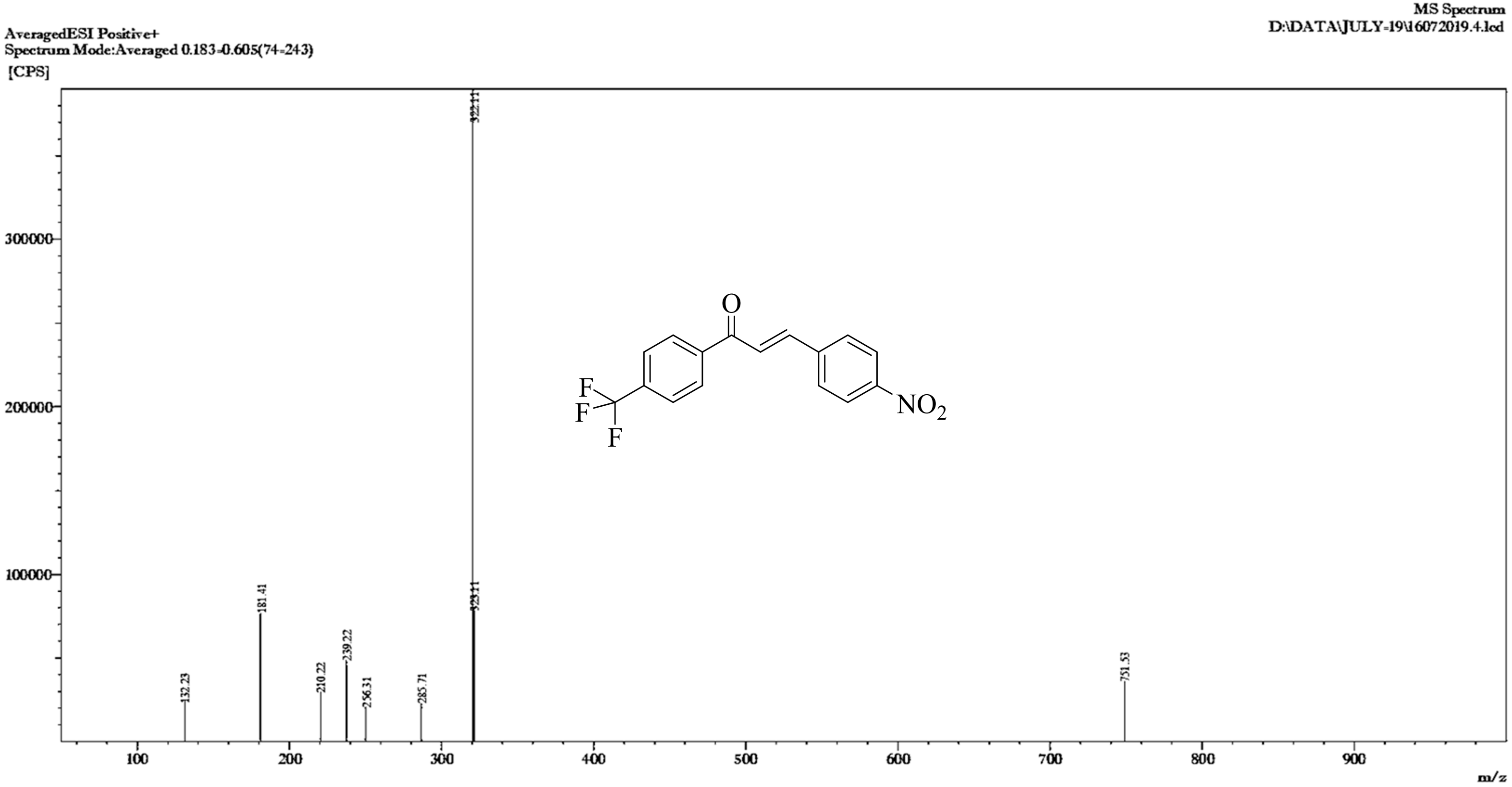

Mass Spectrum of 1-(4-(trifluoromethyl)phenyl)-3-(4-nitrophenyl)prop-2-en-1-one (A9)

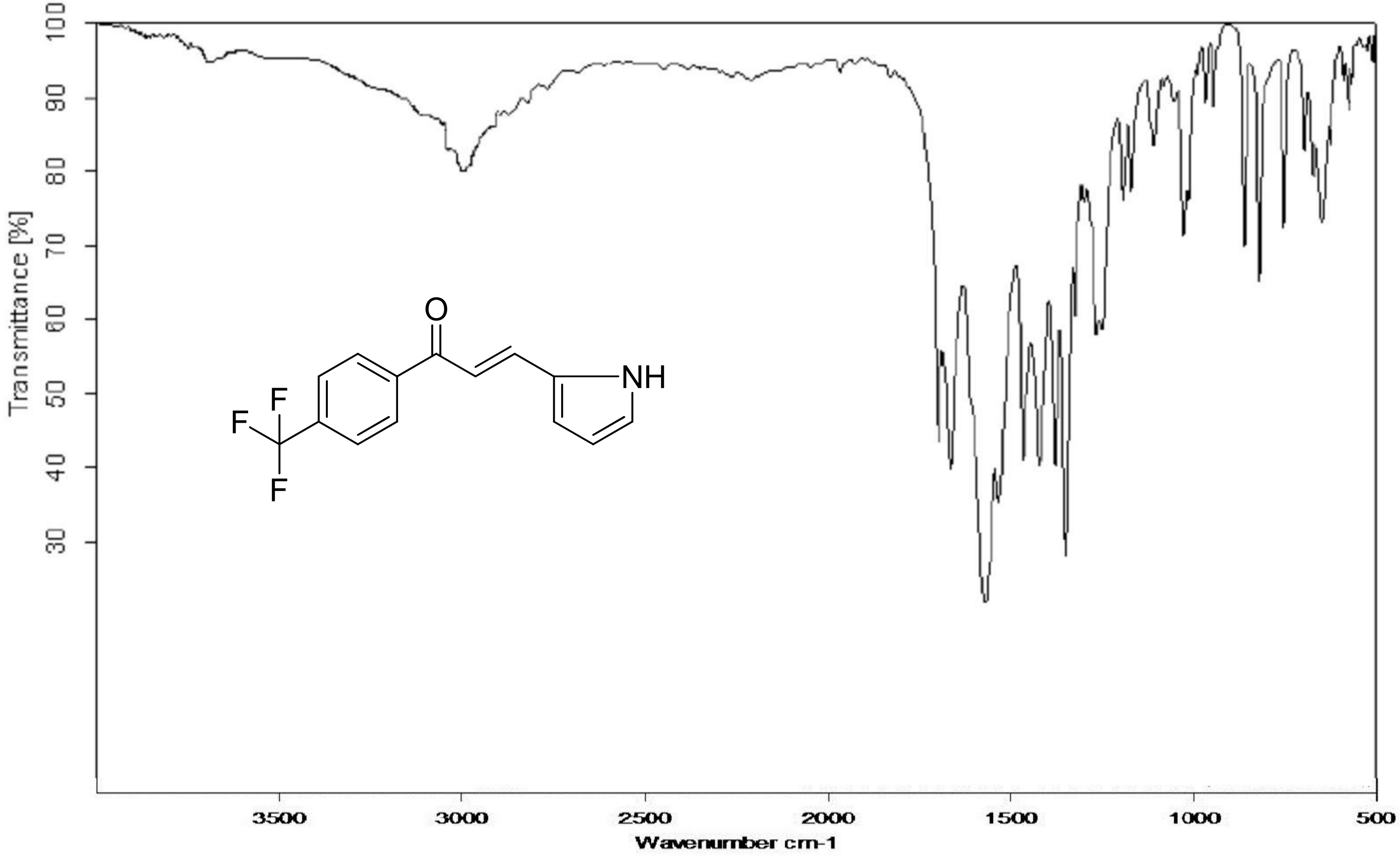

FT-IR Spectrum of (E)-3-(1H-pyrrol-2-yl)-1-(4-(trifluorometh y)phenyl)prop-2-en-1-one (A10)

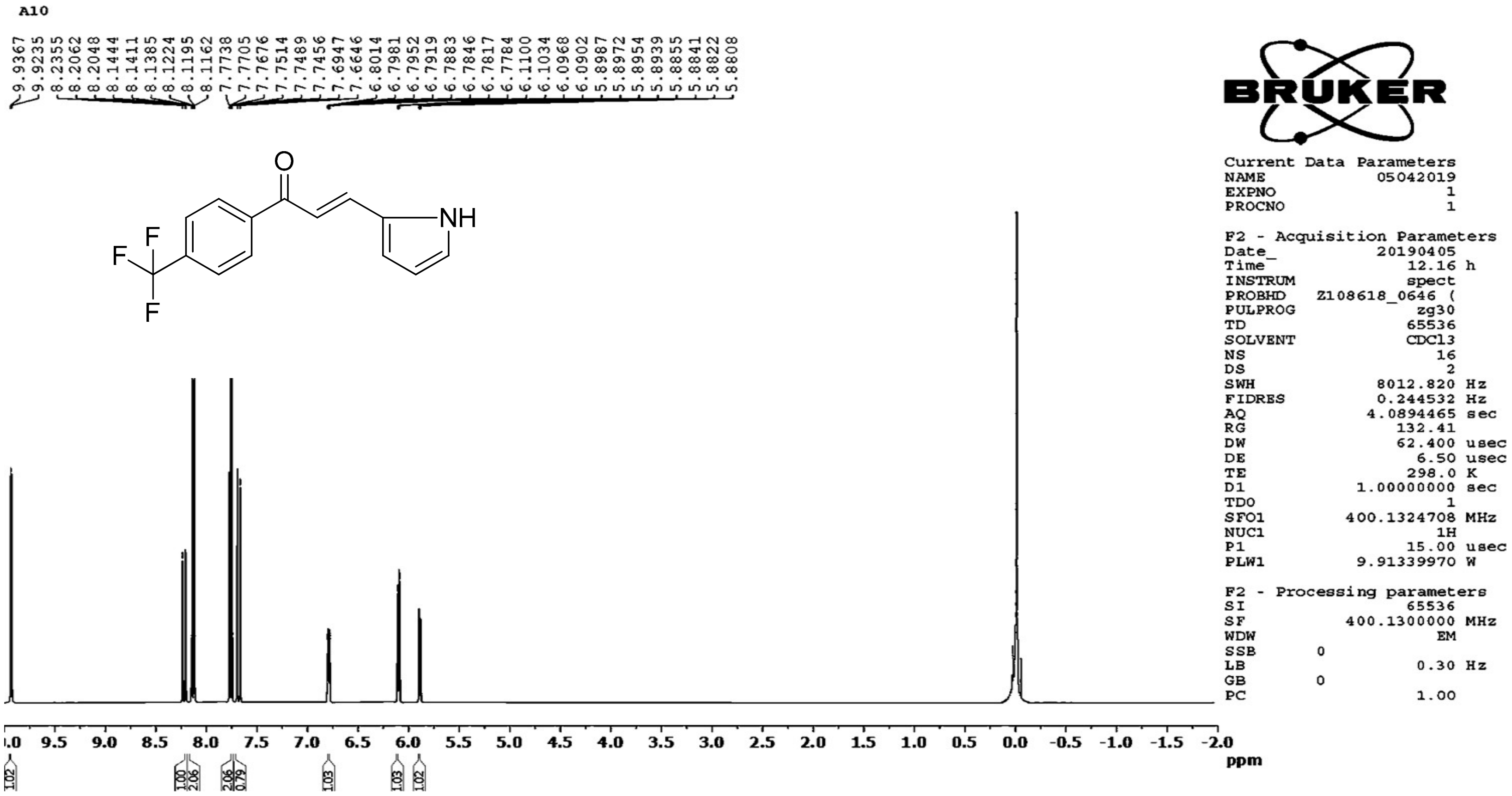

1H NMR Spectrum of (E)-3-(1H-pyrrol-2-yl)-1-(4-(trifluorometyhy)phenyl)prop-2-en-1-one (A10)

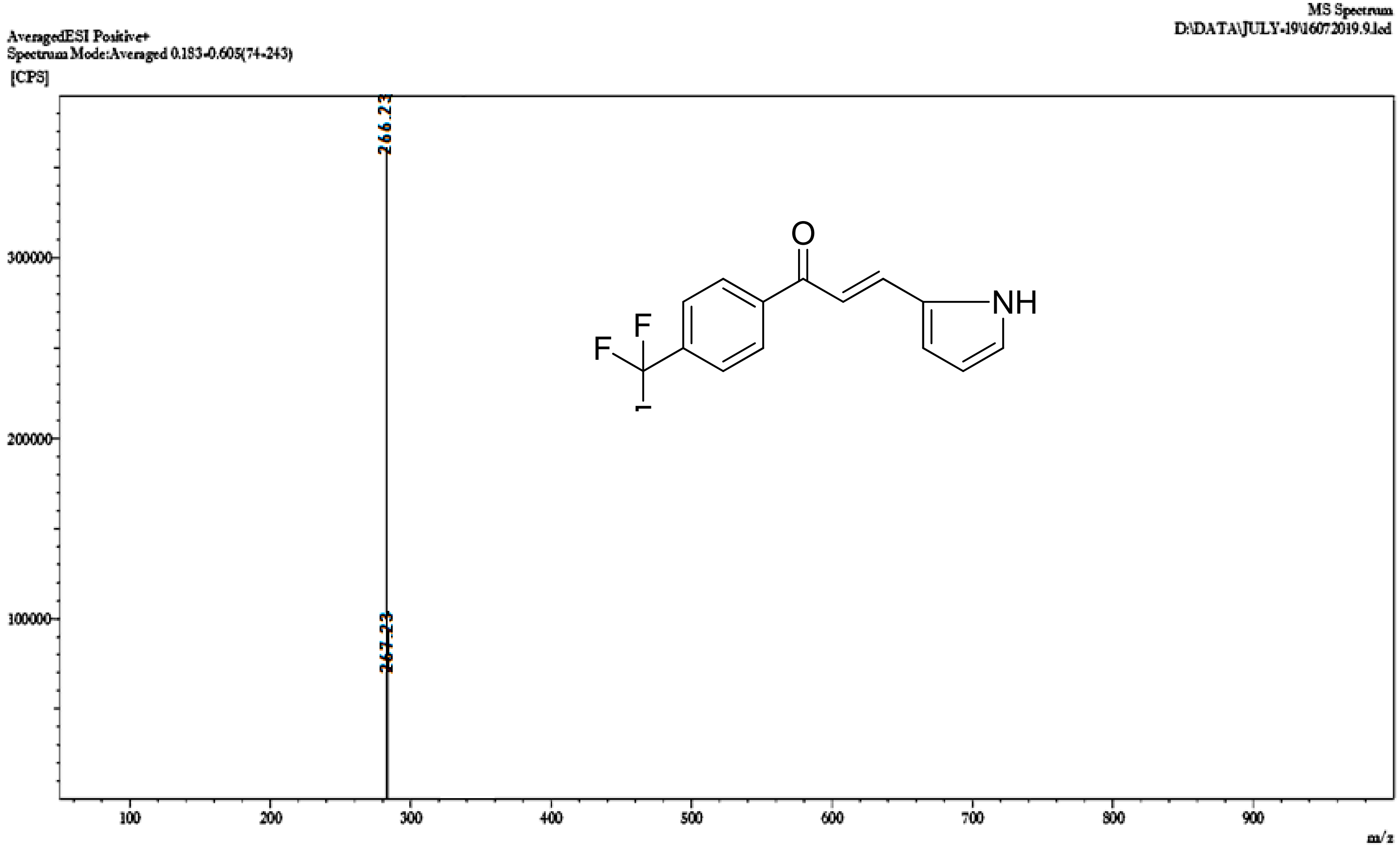

Mass Spectrum of (E)-3-(1H-pyrrol-2-yl)-1-(4-(trifluoromethy)phenyl)prop-2-en-1-one (A10)

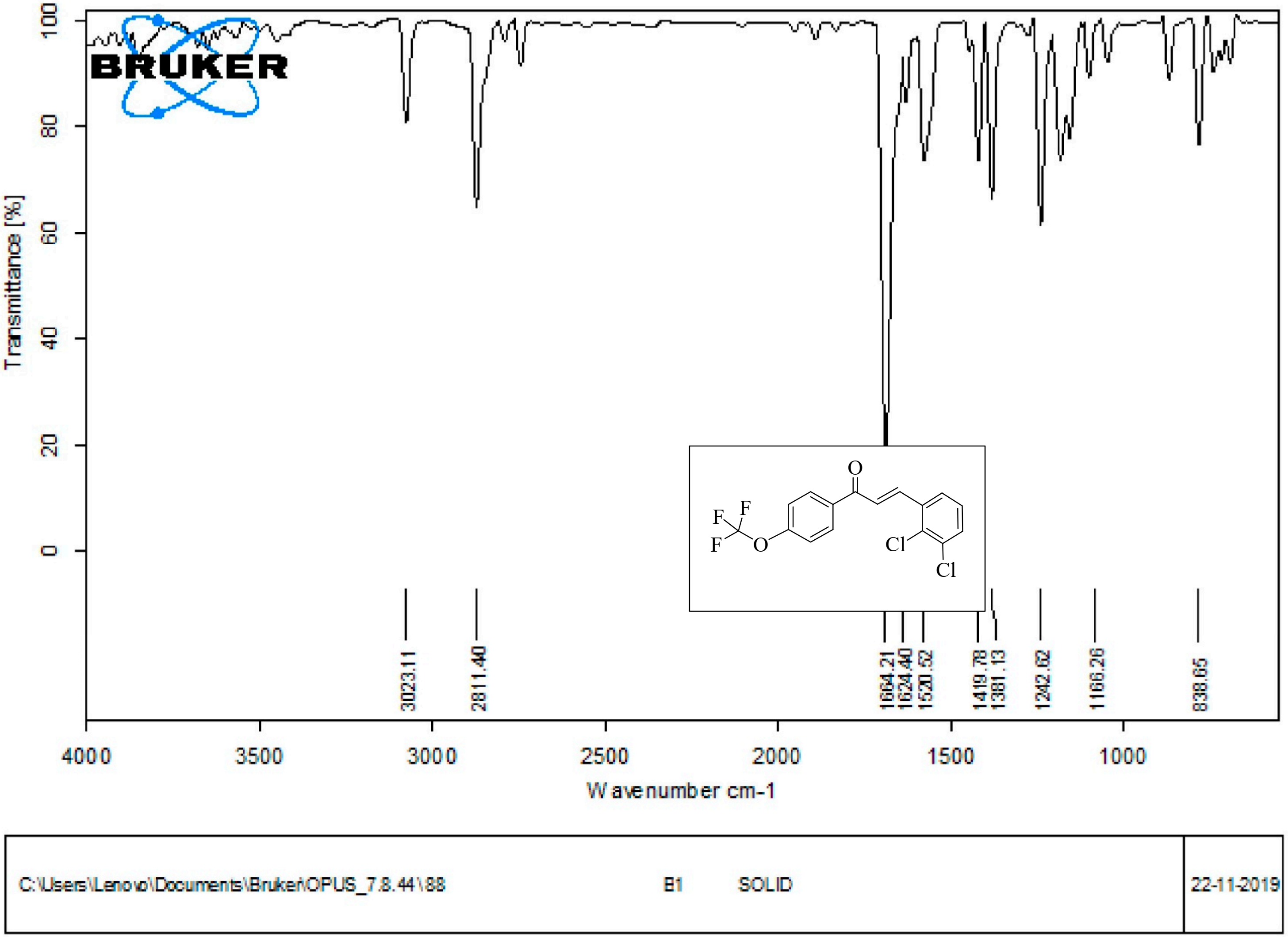

FT-IR Spectrum of *(E)*-3-(2'',3''-dichlorophenyl)-1-[4'-(trifluoromethoxy)phenyl]prop-2-en-1-one (B1)

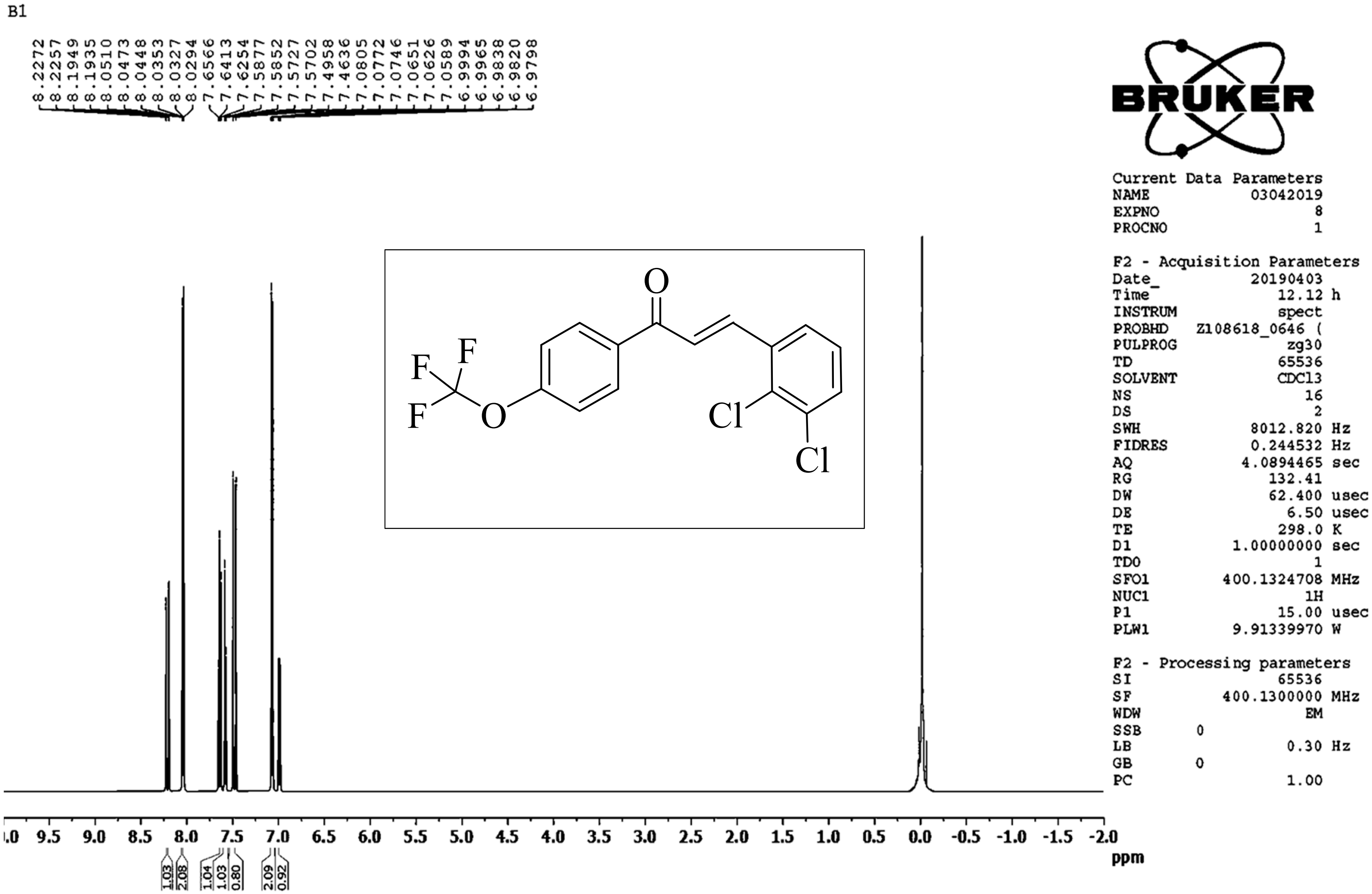

^{1}H NMR Spectrum of 3-(2'',3''-dichlorophenyl)-1-[4'-(trifluoromethoxy)phenyl]prop-2-en-1-one (B1)

Sample Name : **KSB-B1-361**
Data File : **16072019.8.lcd**
Date Acquired : **7/16/2019 11:42:16 AM**
Batch File : **16072019.lcb**

MS Spectrum
D:\DATA\JULY-19\16072019.8.lcd

AveragedESI Positive+
Spectrum Mode:Averaged 0.183-0.605(74-243)

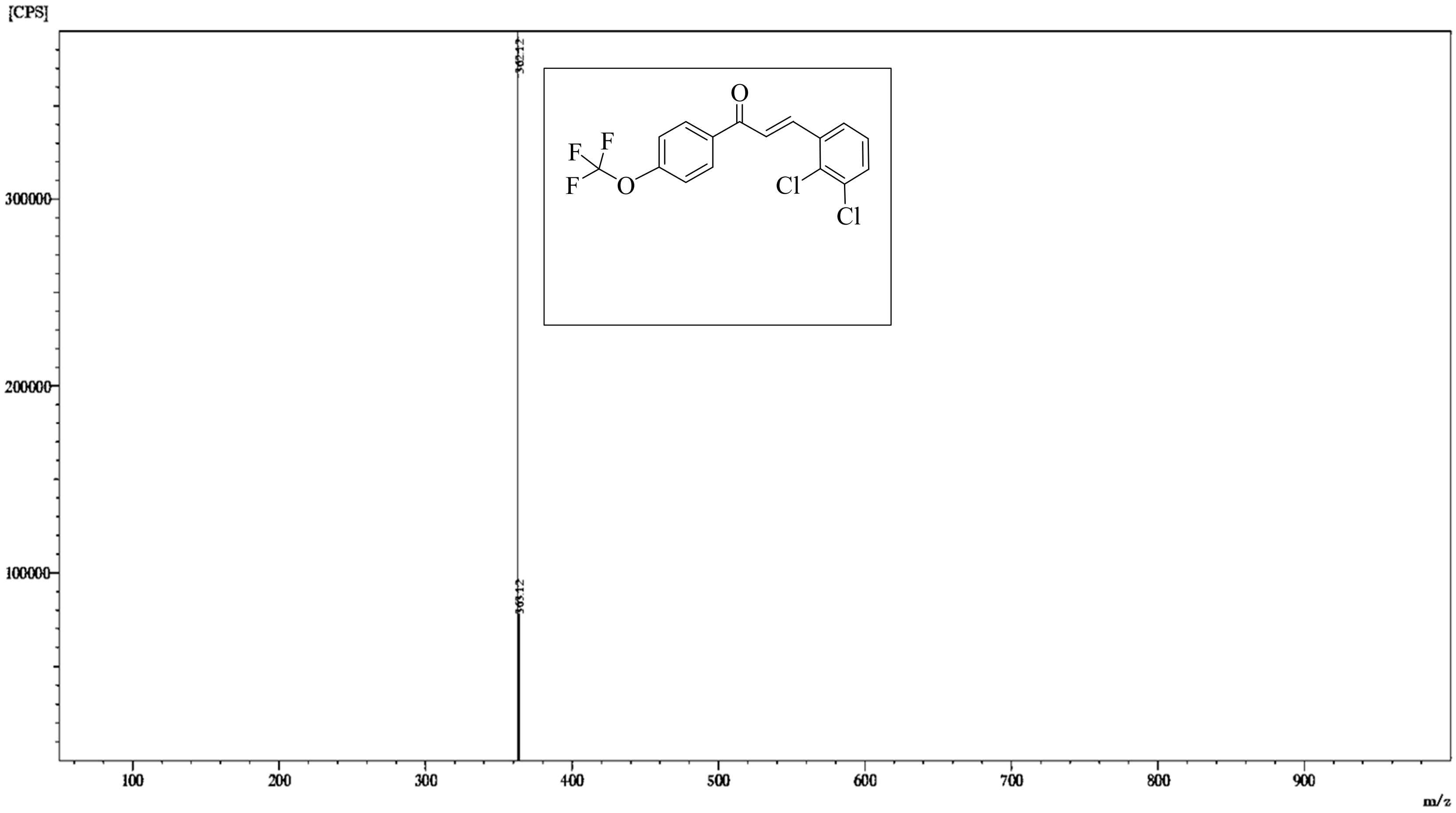

MASS Spectrum of 3-(2'',3''-dichlorophenyl)-1-[4'-(trifluoromethoxy)phenyl]prop-2-en-1-one (B1)

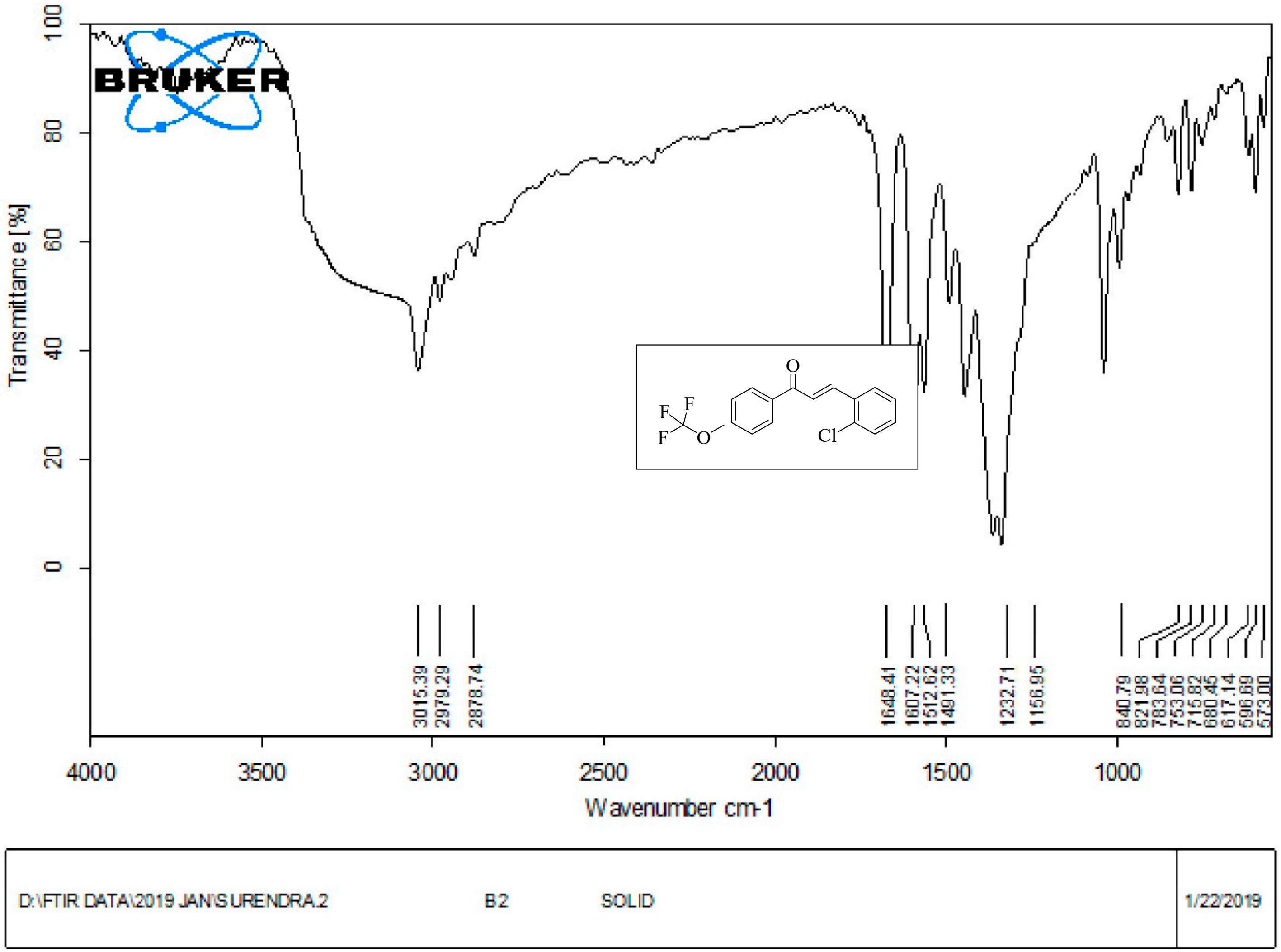

FT-IR Spectrum of 3-(2''-chlorophenyl)-1-[4'-(trifluoromethoxy)phenyl]prop-2-en-1-one (B2)

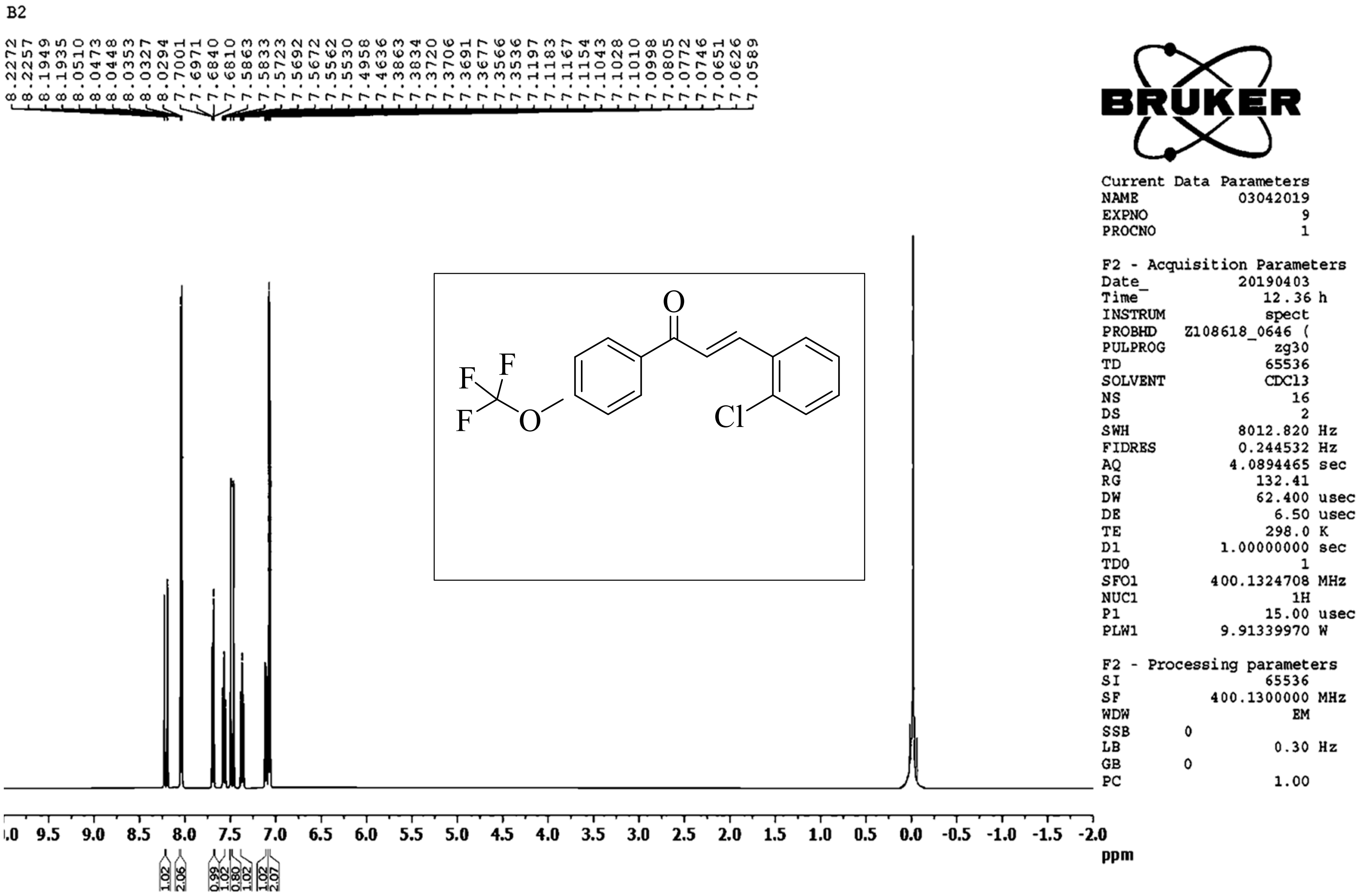

^{1}H NMR Spectrum of 3-(2''-chlorophenyl)-1-[4'-(trifluoromethoxy)phenyl]prop-2-en-1-one (B2)

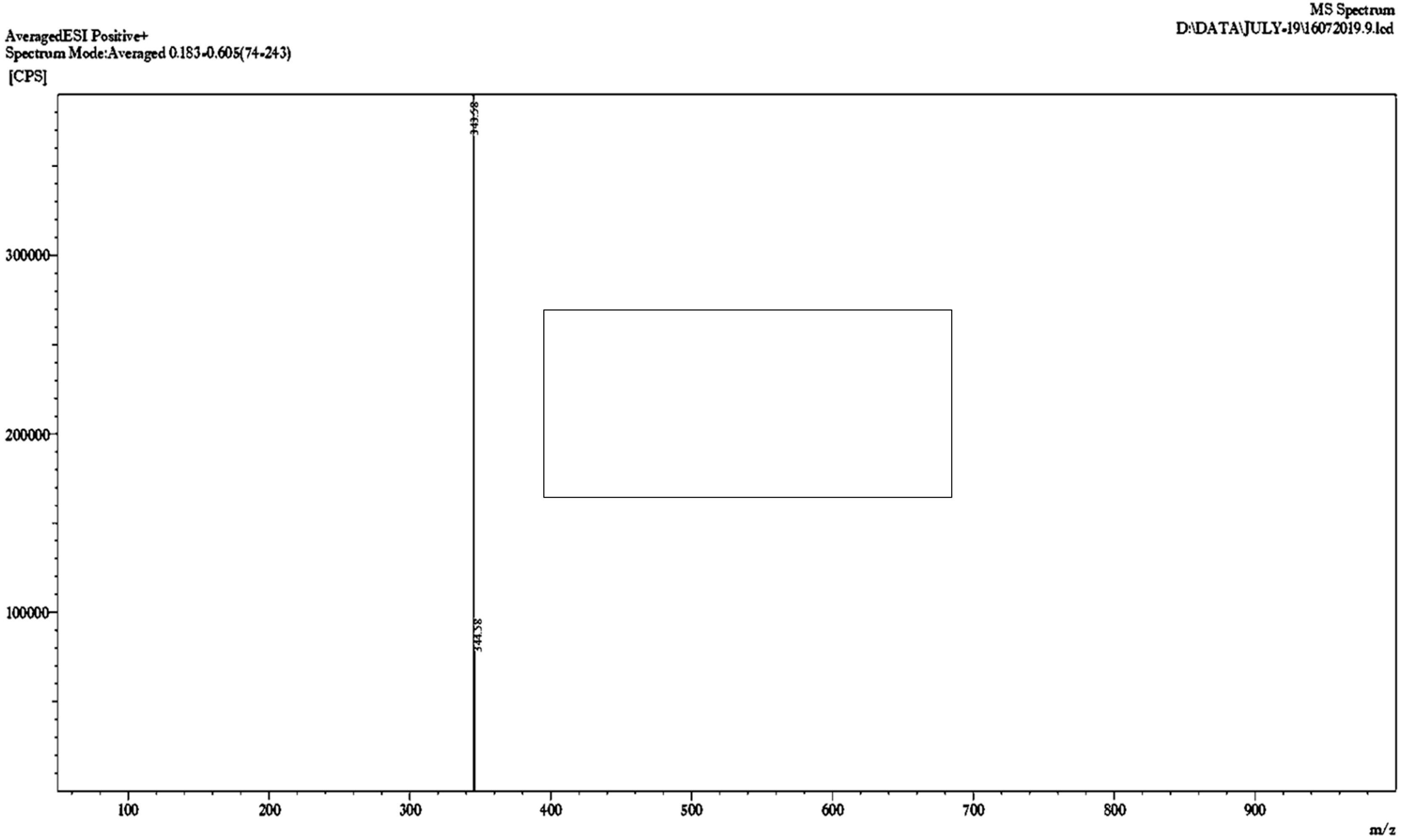

MASS Spectrum of 3-(2''-chlorophenyl)-1-[4'-(trifluoromethoxy)phenyl]prop-2-en-1-one (B2)

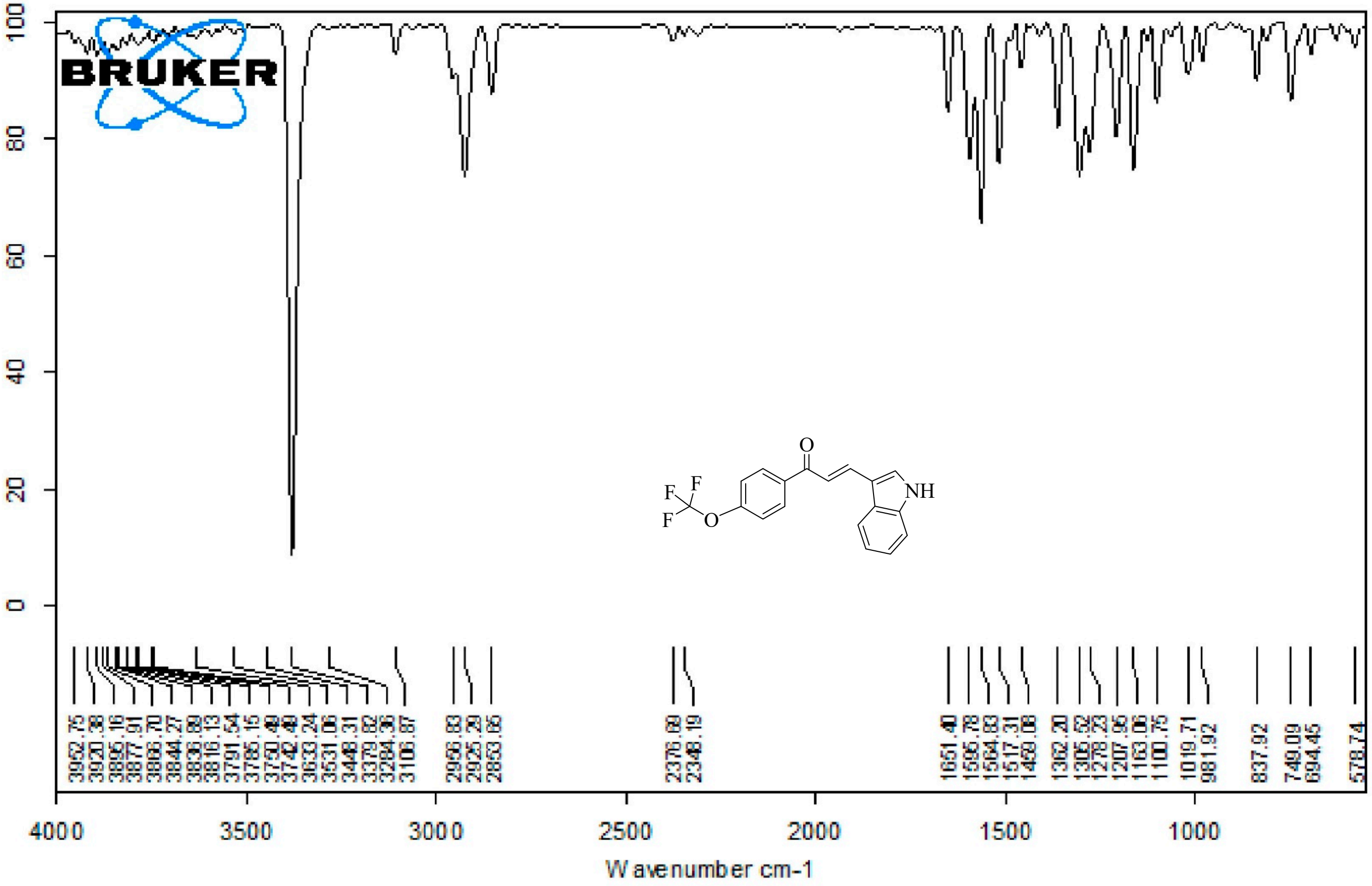

C:\Users\Lenovo\Documents\Bruker\OPUS_7.8.44\DATA\MEAS\ANURADHA.52	B3	SOLID		19-09-2019

FT- IR Spectrum of (E)-3-(1''H-indol-3''-yl)-1-[4'-(trifluoromethoxy)phenyl]prop-2-en-1-one (B3)

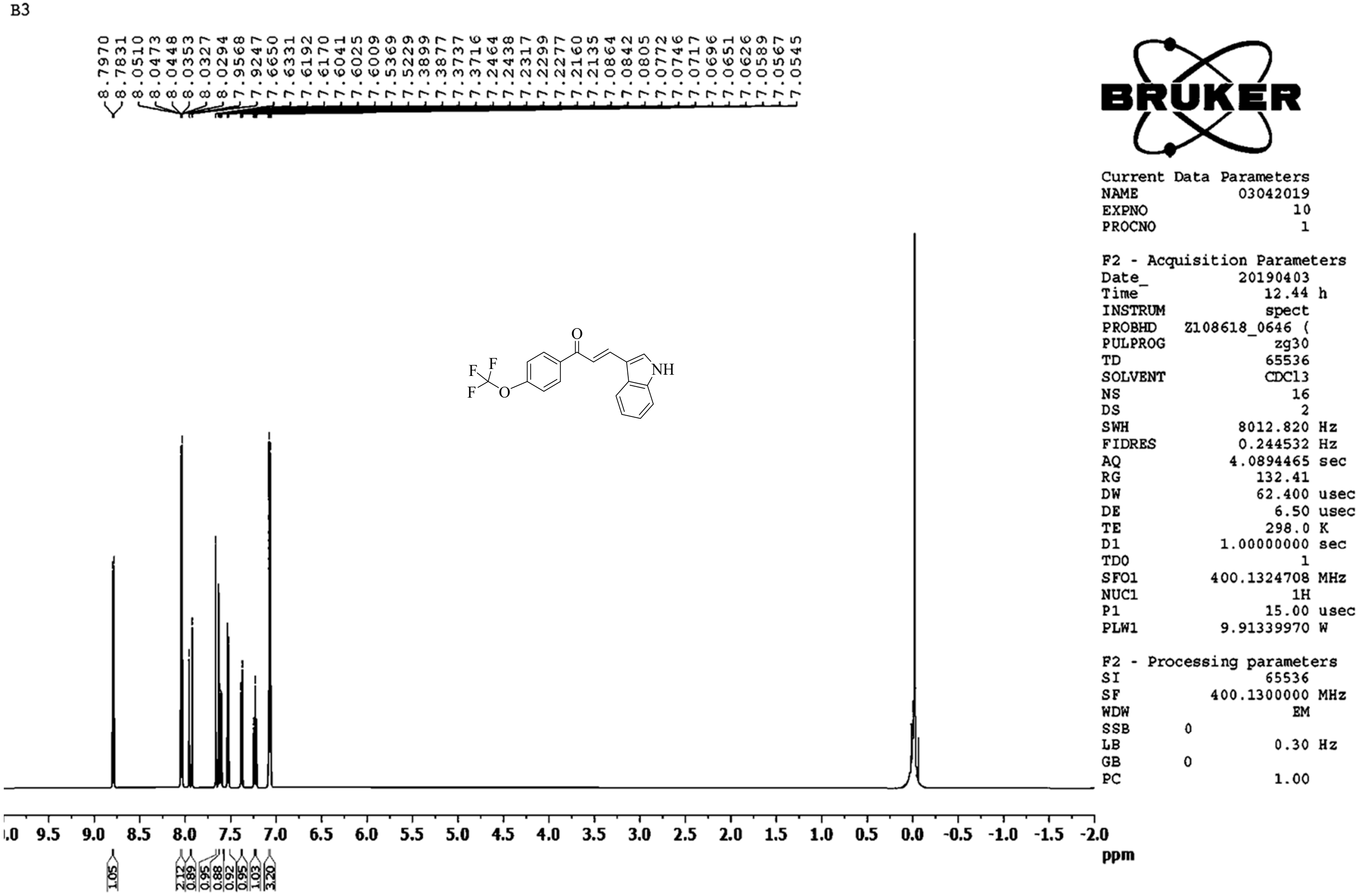

^{1}H NMR Spectrum of (E)-3-(1''H-indol-3''-yl)-1-[4'-(trifluoromethoxy)phenyl]prop-2-en-1-one (B3)

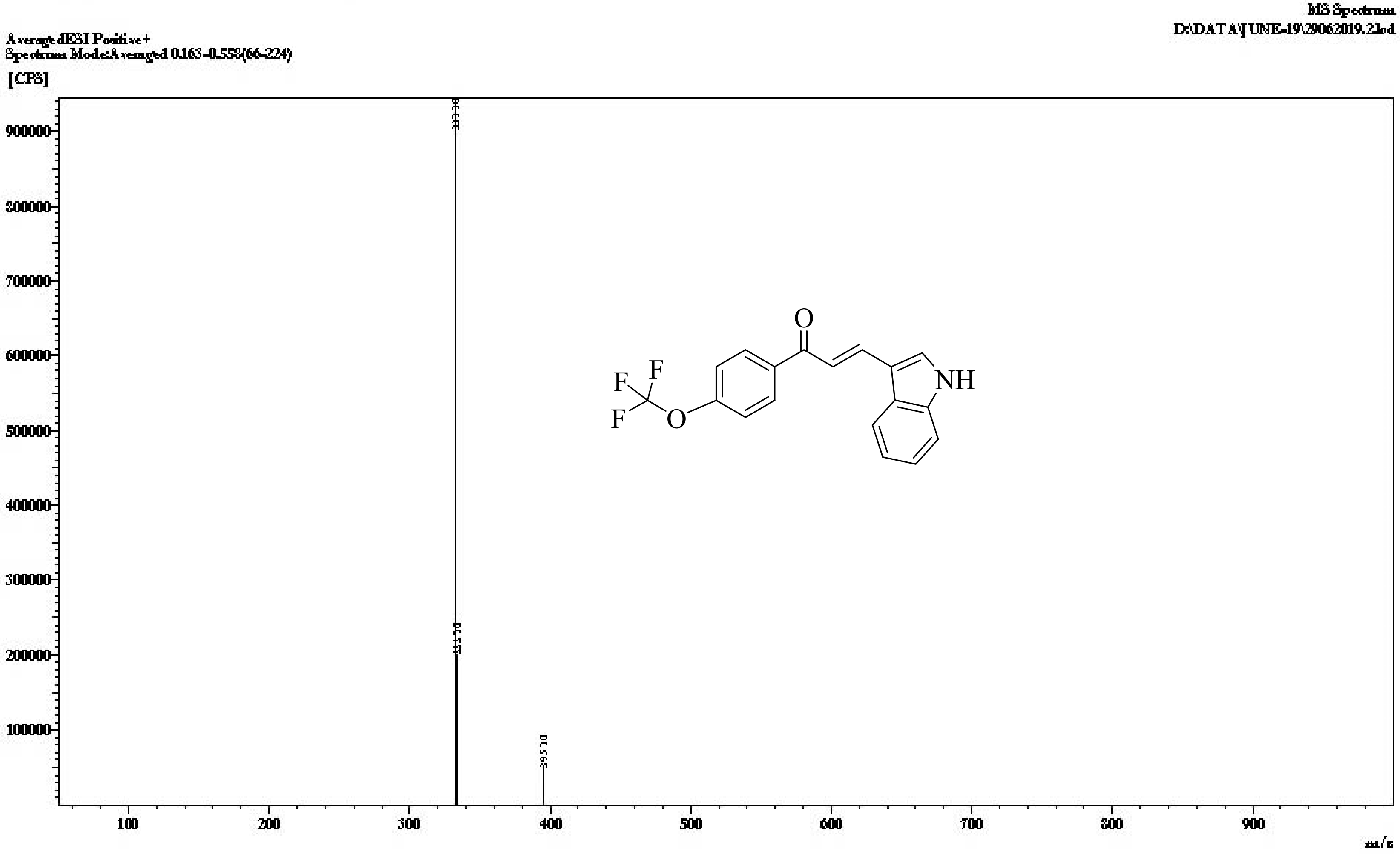

MASS Spectrum of (E)-3-(1''H-indol-3''-yl)-1-[4'-(trifluoromethoxy)phenyl]prop-2-en-1-one (B3)

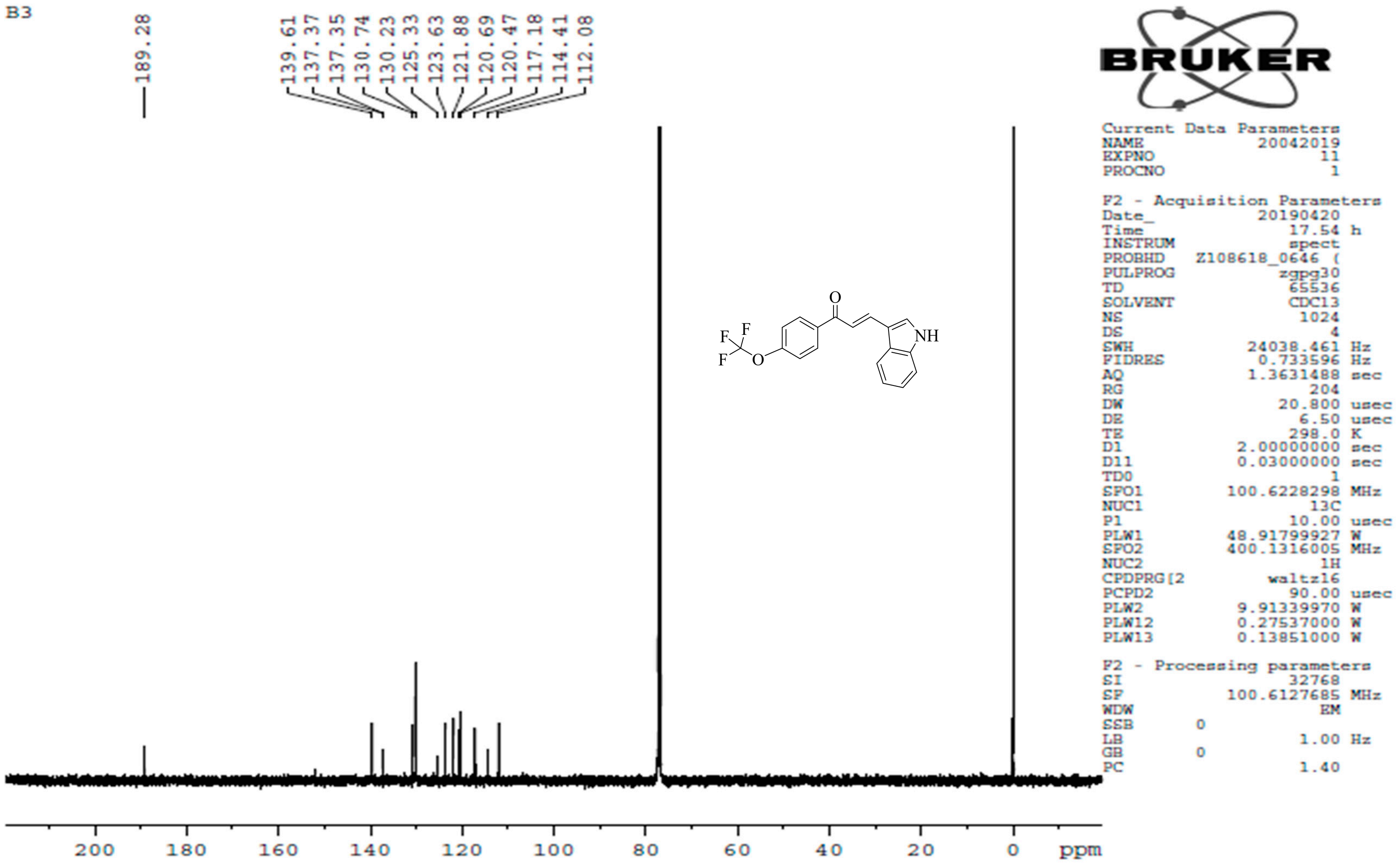

^{13}C NMR Spectrum of (E)-3-(1''H-indol-3''-yl)-1-[4'-(trifluoromethoxy)phenyl]prop-2-en-1-one (B3)

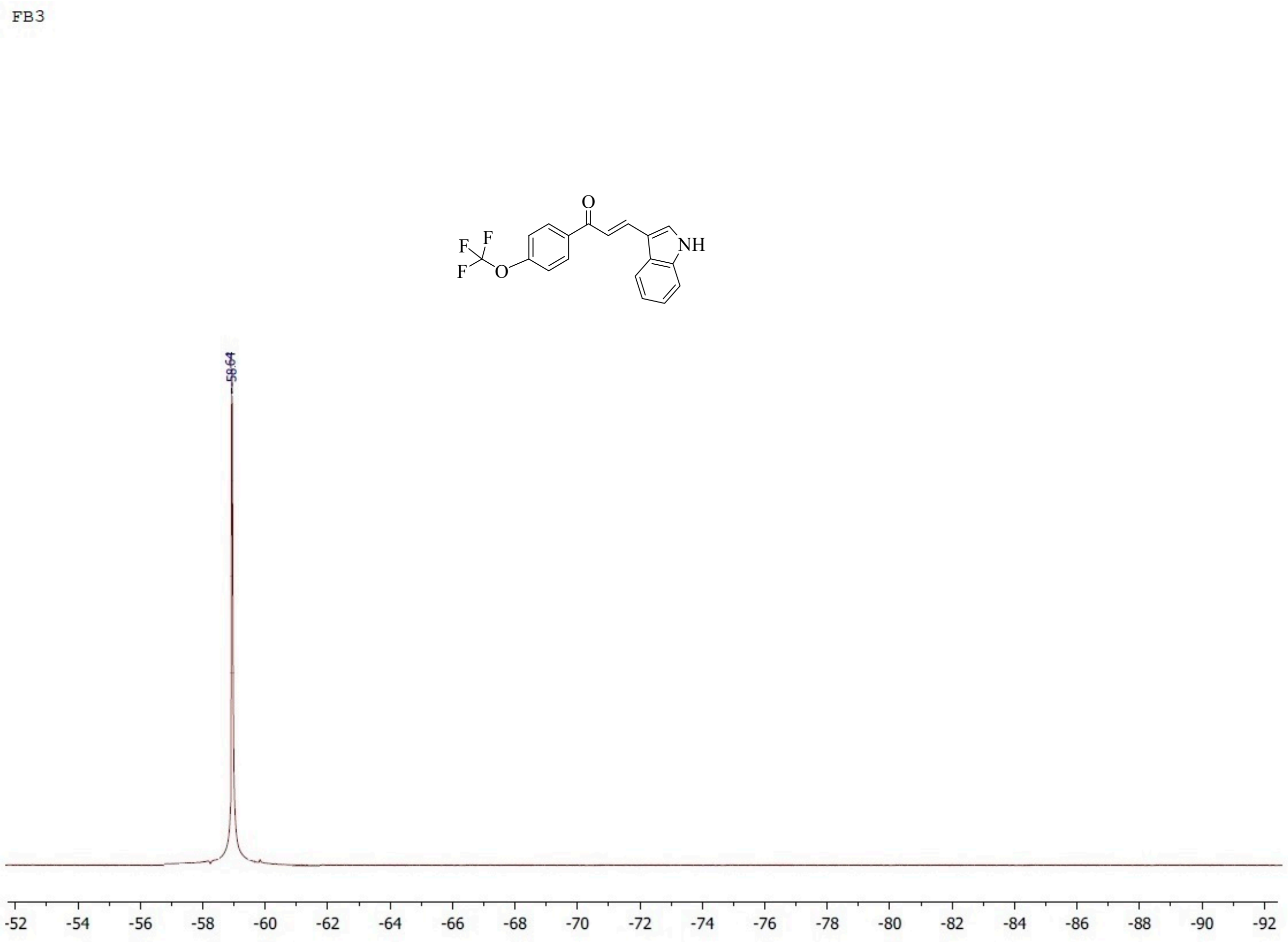

Current Data Parameters
NAME 24042019
EXPNO 1
PROCNO 1

F2 - Acquisition Parameters
Date_ 20190424
Time_ 12.32 h
INSTRUM spect
PROBHD Z108618_0646 (
PULPROG zg30
TD 65536
SOLVENT CDCl3
NS 16
DS 2
SWH 8012.820 Hz
FIDRES 0.244532 Hz
AQ 4.0894465 sec
RG 132.41
DW 62.400 usec
DE 6.50 usec
TE 298.0 K
D1 1.00000000 sec
TD0 1
SFO1 376.1524708 MHz
NUC1 1H
P1 15.00 usec
PLW1 9.91339970 W

F2 - Processing parameters
SI 65536
SF 376.1500000 MHz
WDW EM
SSB 0
LB 0.30 Hz
GB 0
PC 1.00

^{10}F Spectrum of (E)-3-(1''H-indol-3''-yl)-1-[4'-(trifluoromethoxy)phenyl]prop-2-en-1-one (B3)

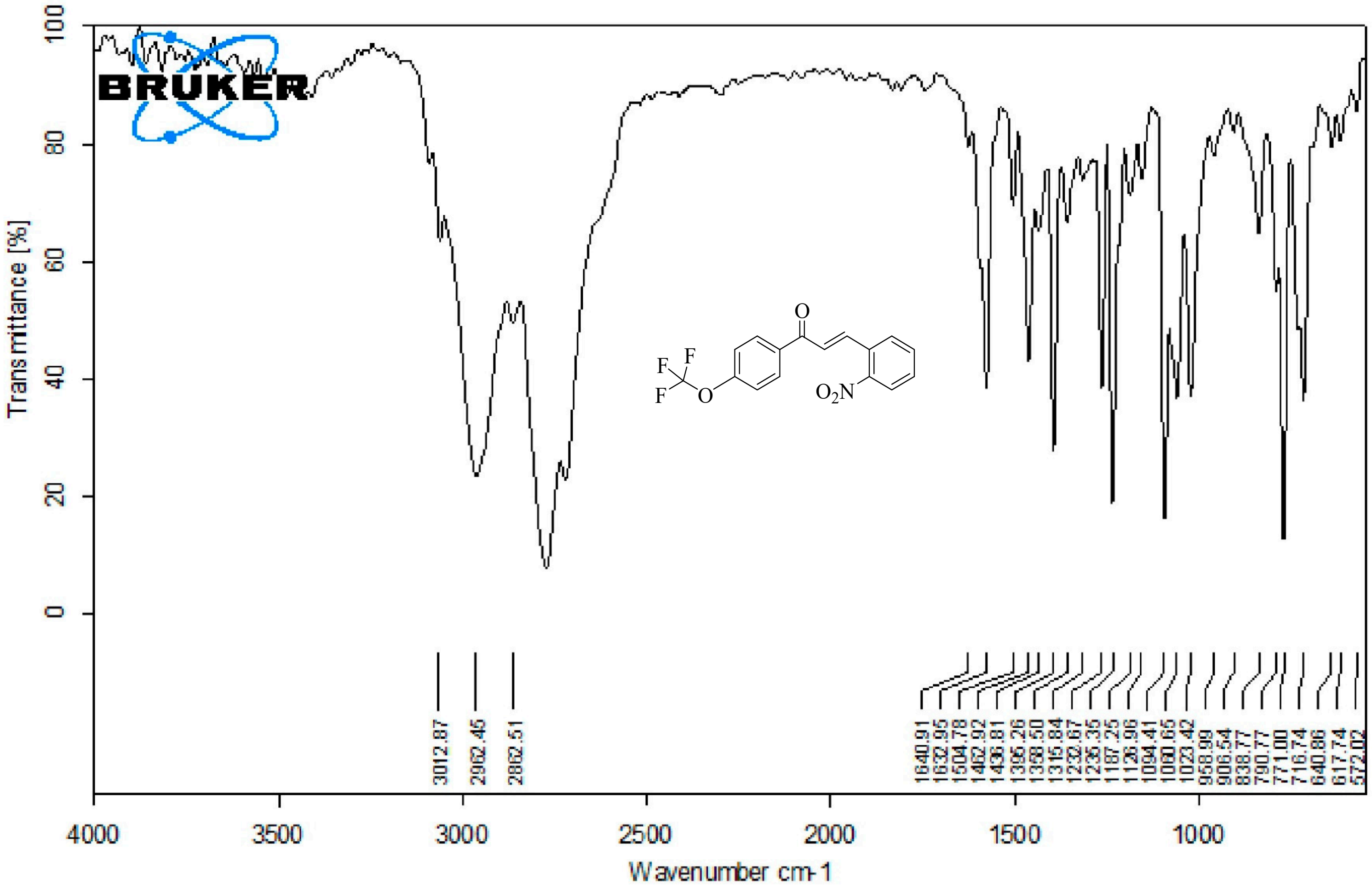

D:\FTIR DATA\2019 JAN\SURENDRA.29 B4	SOLID		1/23/2019

FT-IR Spectrum of (E)- 1-(4-(trifluoromethoxy)phenyl)-3-(2-nitrophenyl)prop-2-en-1-one (B4)

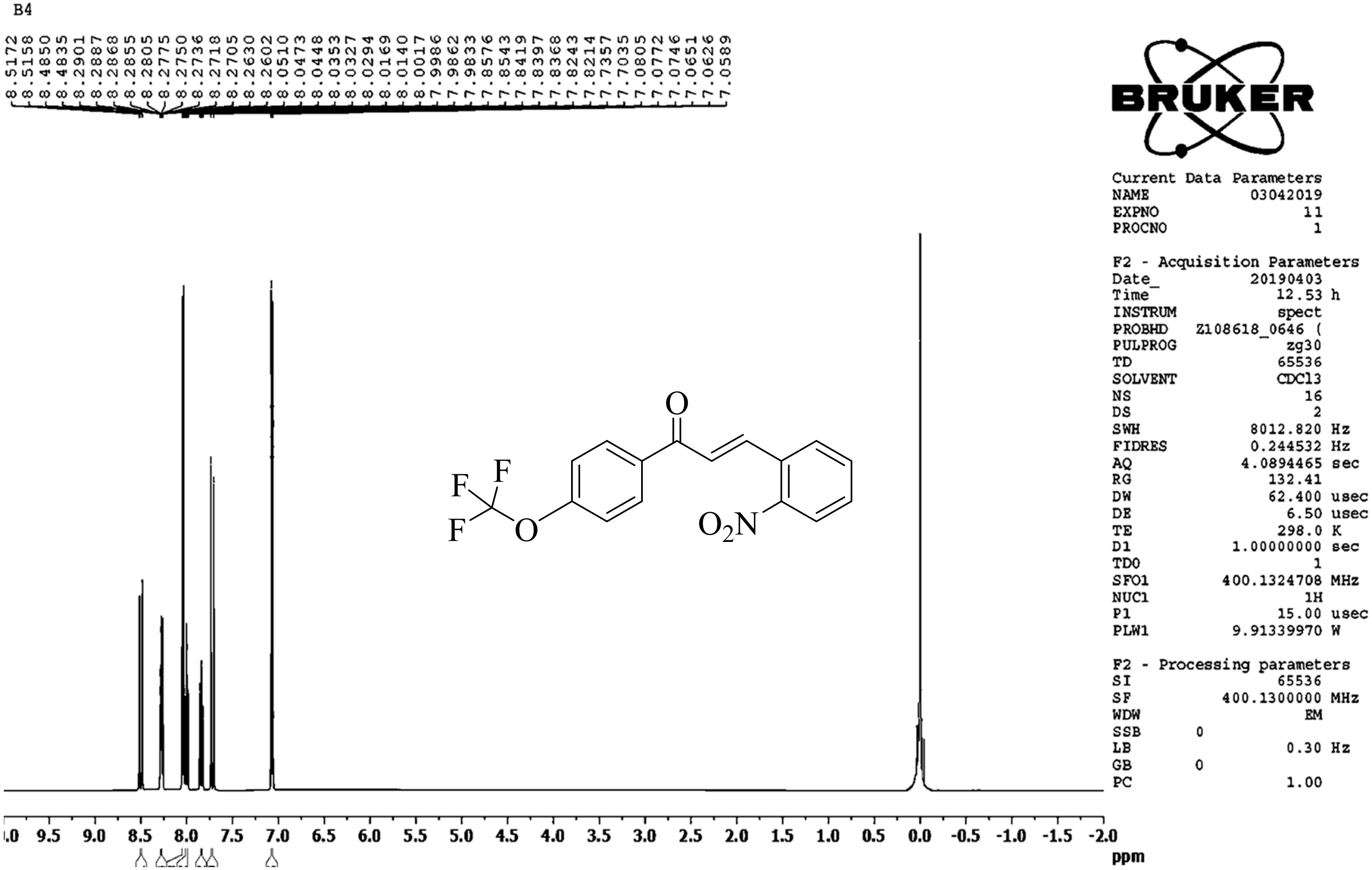

^{1}H NMR Spectrum of (E)-1-(4-(trifluoromethoxy)phenyl)-3-(2-nitrophenyl)prop-2-en-1-one (B4)

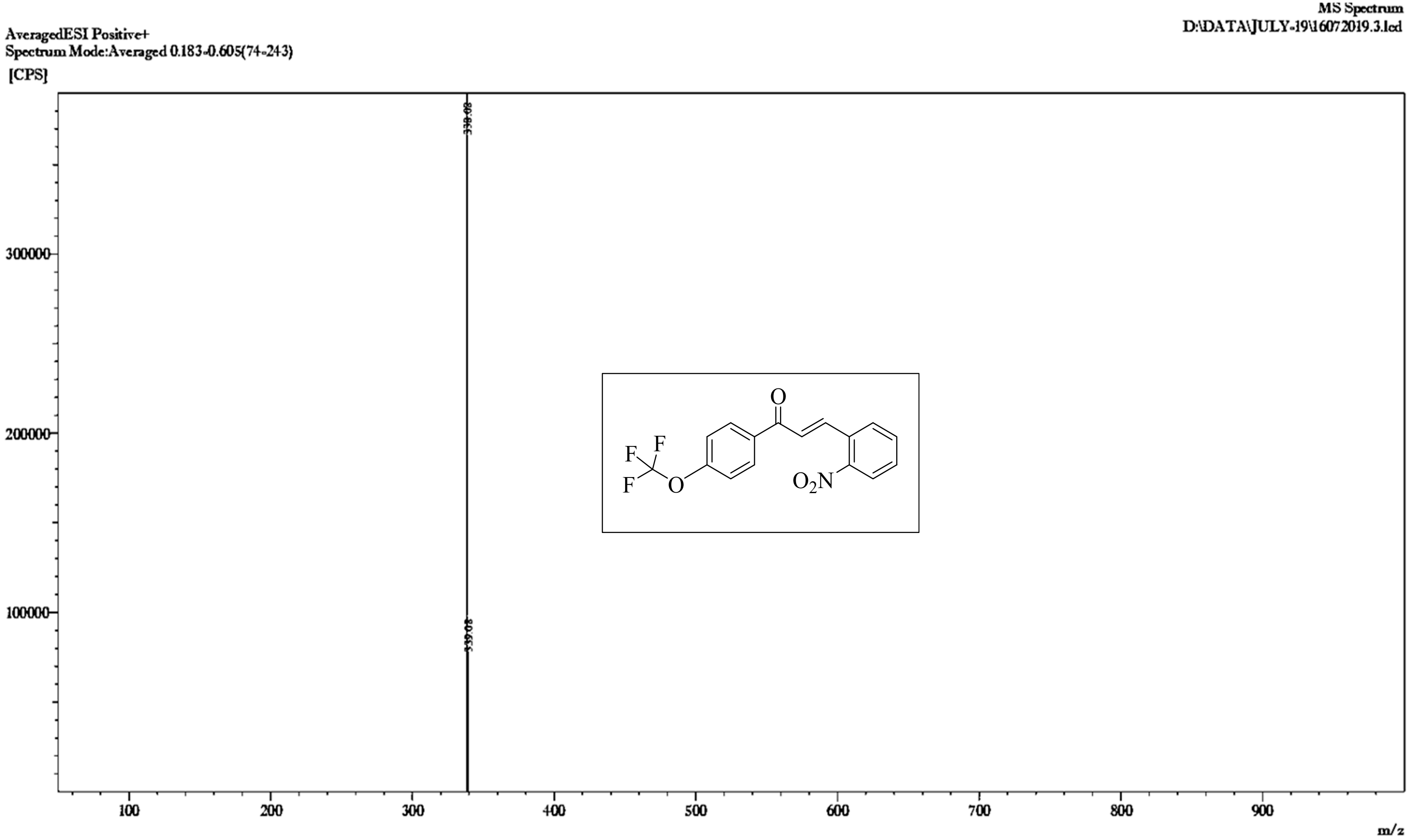

MASS Spectrum of (E)-1-(4-(trifluoromethoxy)phenyl)-3-(2-nitrophenyl)prop-2-en-1-one (B4)

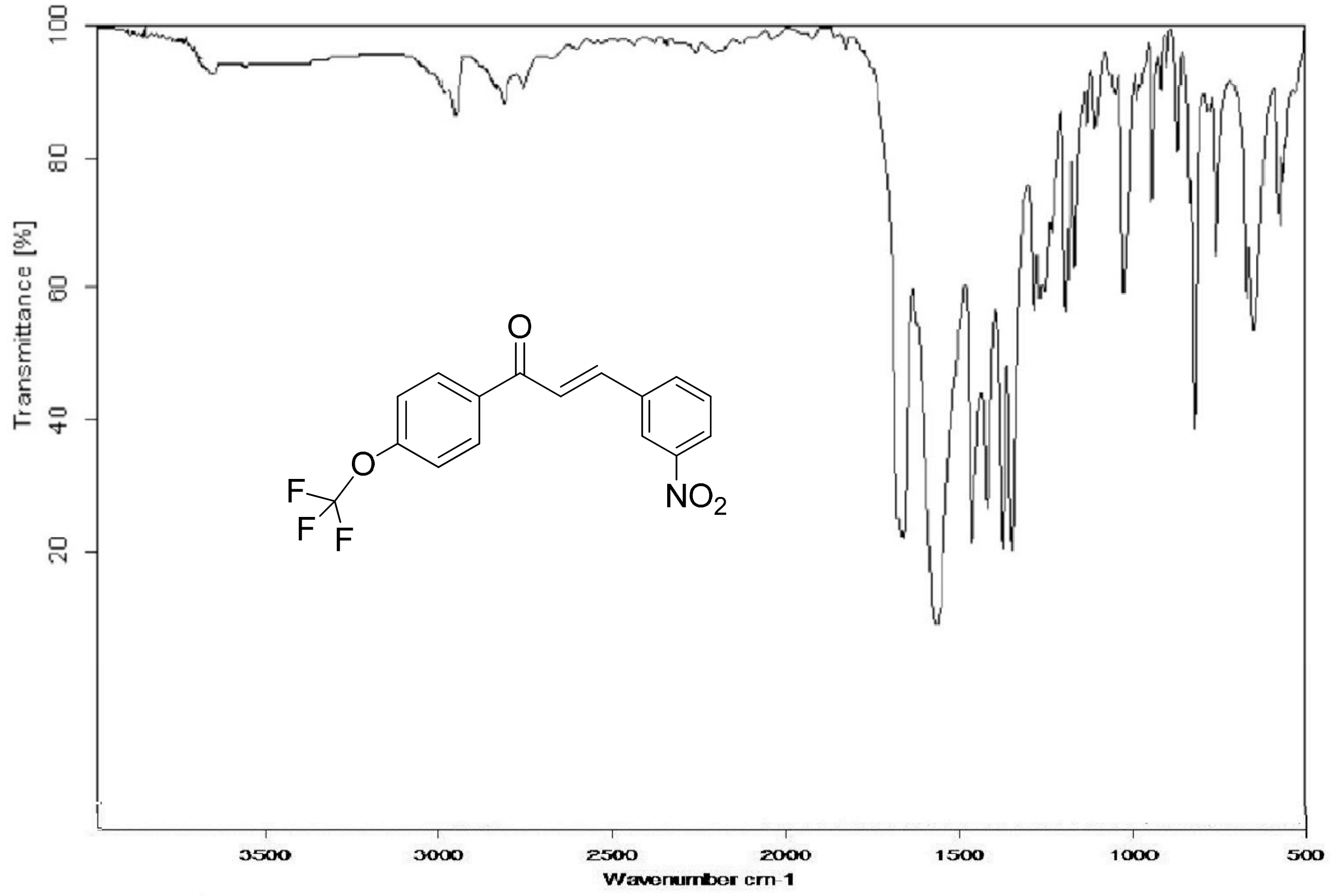

1H NMR **Spectrum of *(E)*-1-(4-(trifluoromethoxy)phenyl)-3-(3-nitrophenyl)prop-2-en-1-one (B5)**

B5

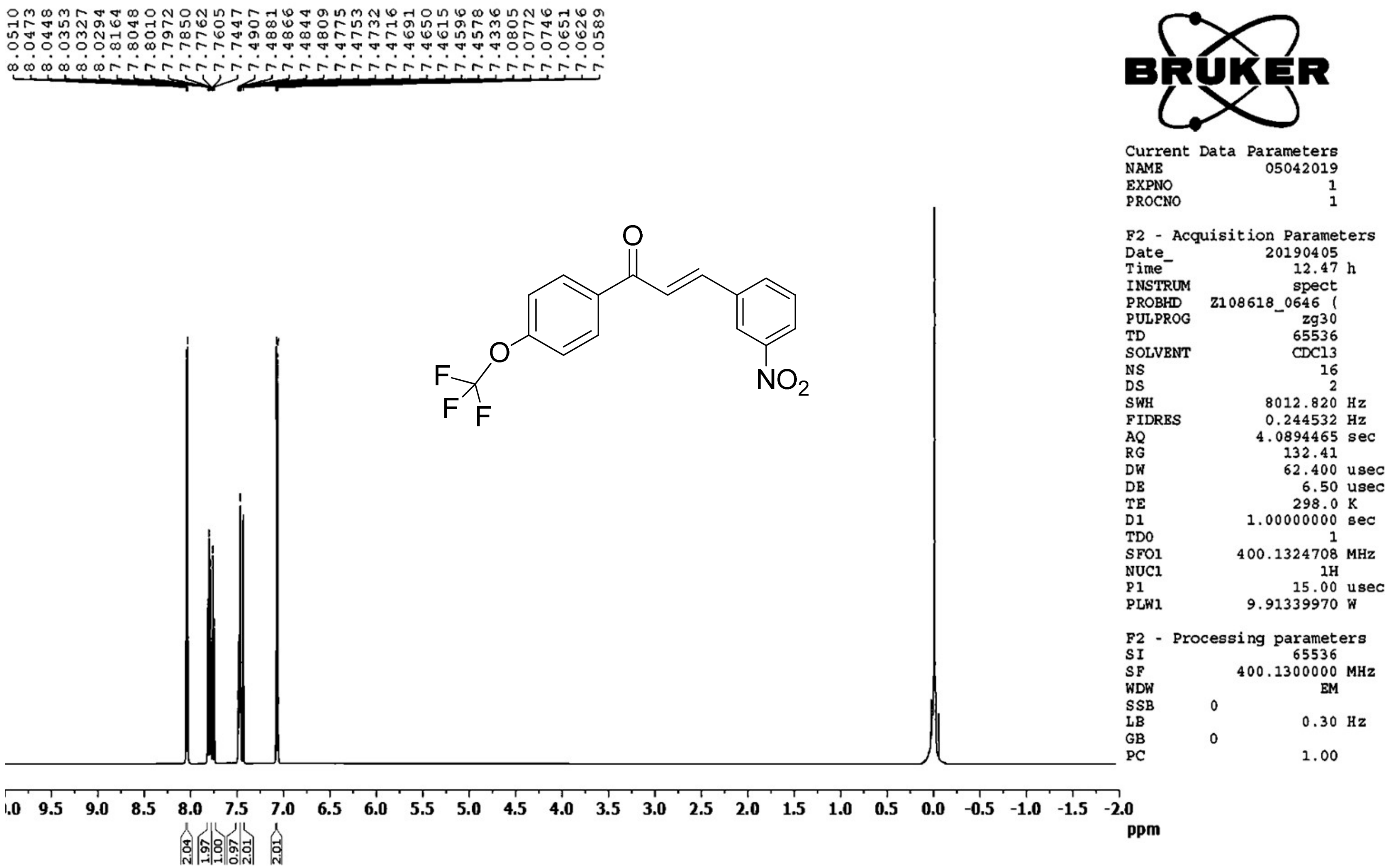

1H NMR **Spectrum of (E)- 1-(4-(trifluoromethoxy)phenyl)-3-(3-nitrophenyl)prop-2-en-1-one (B5)**

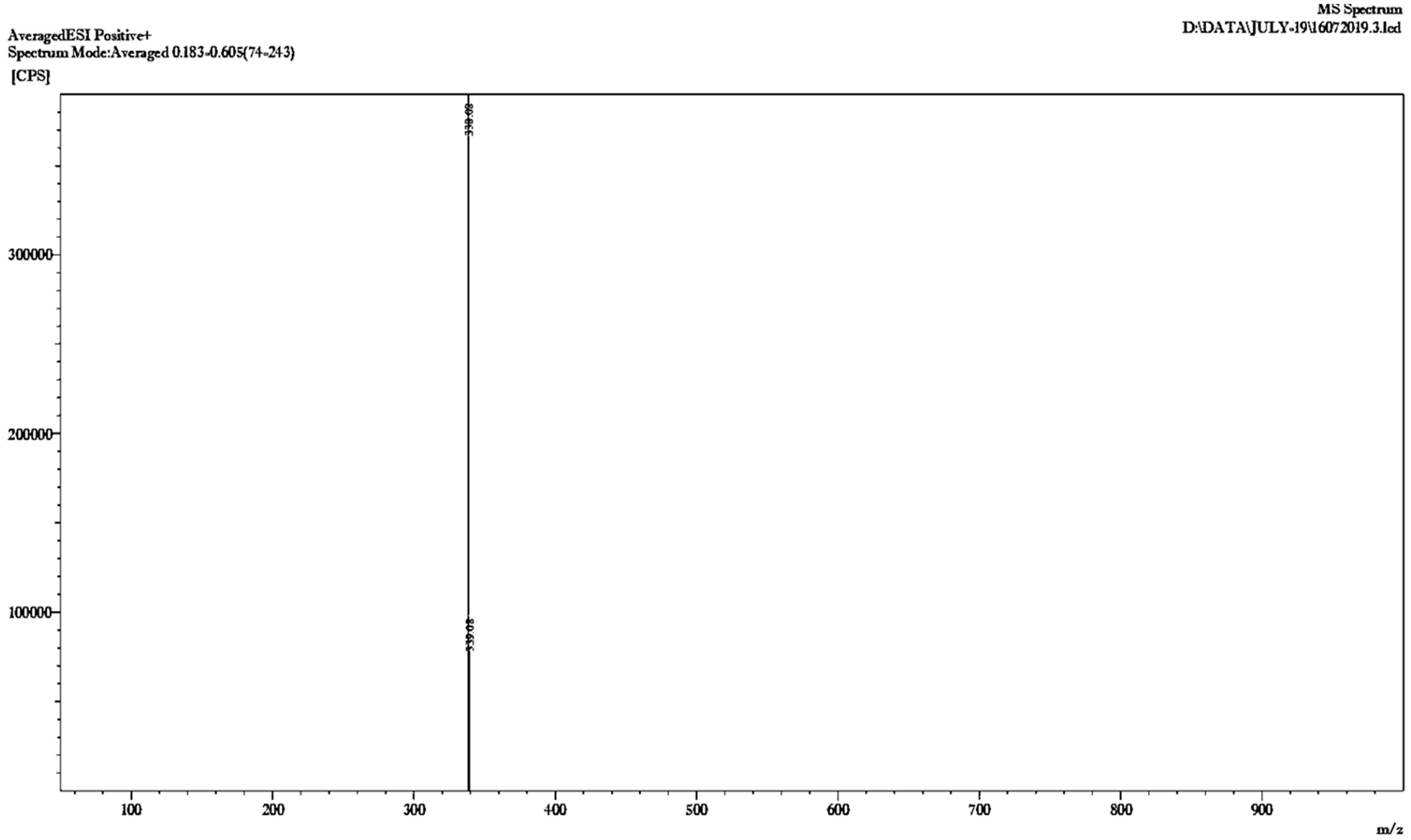

MASS Spectrum of (E)- 1-(4-(trifluoromethoxy)phenyl)-3-(3-nitrophenyl)prop-2-en-1-one (B5)

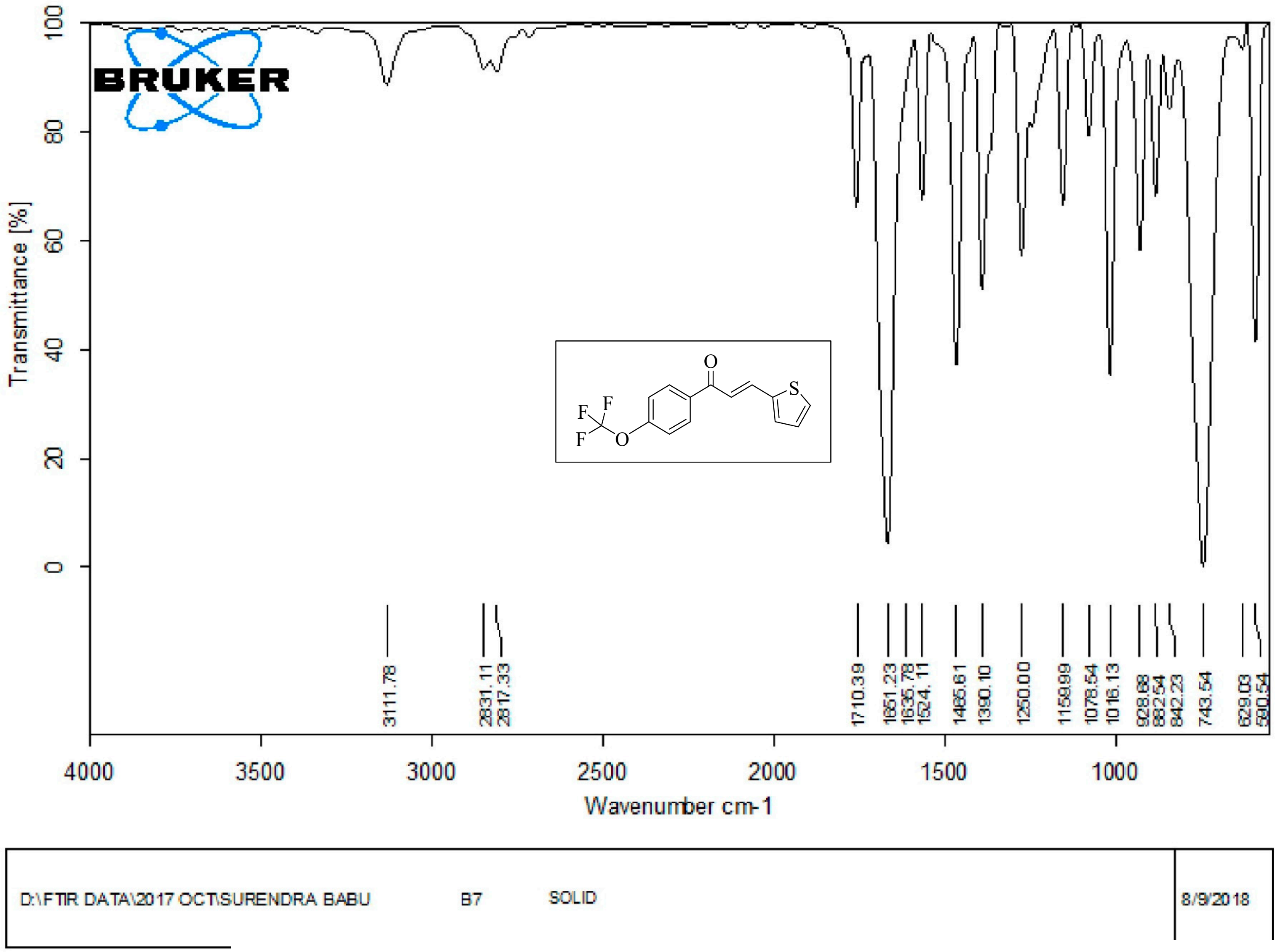

FT-IR Spectrum of (E)- 3-(thiophen-2''-yl)-1-[4'-(trifluoromethoxy)phenyl]prop-2-en-1-one (B6)

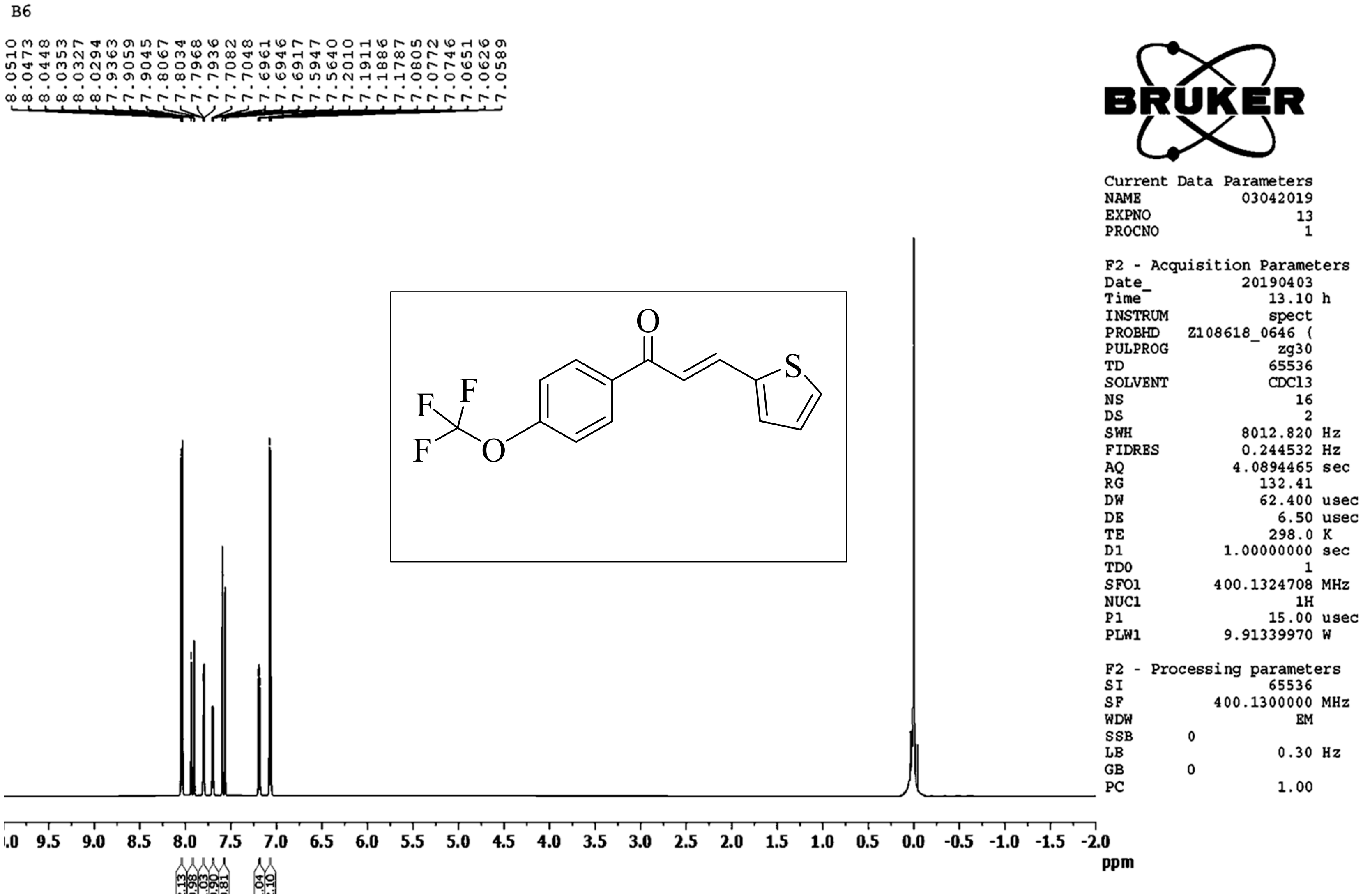

^{1}H NMR Spectrum of (E)- 3-(thiophen-2''-yl)-1-[4'-(trifluoromethoxy)phenyl]prop-2-en-1-one (B6)

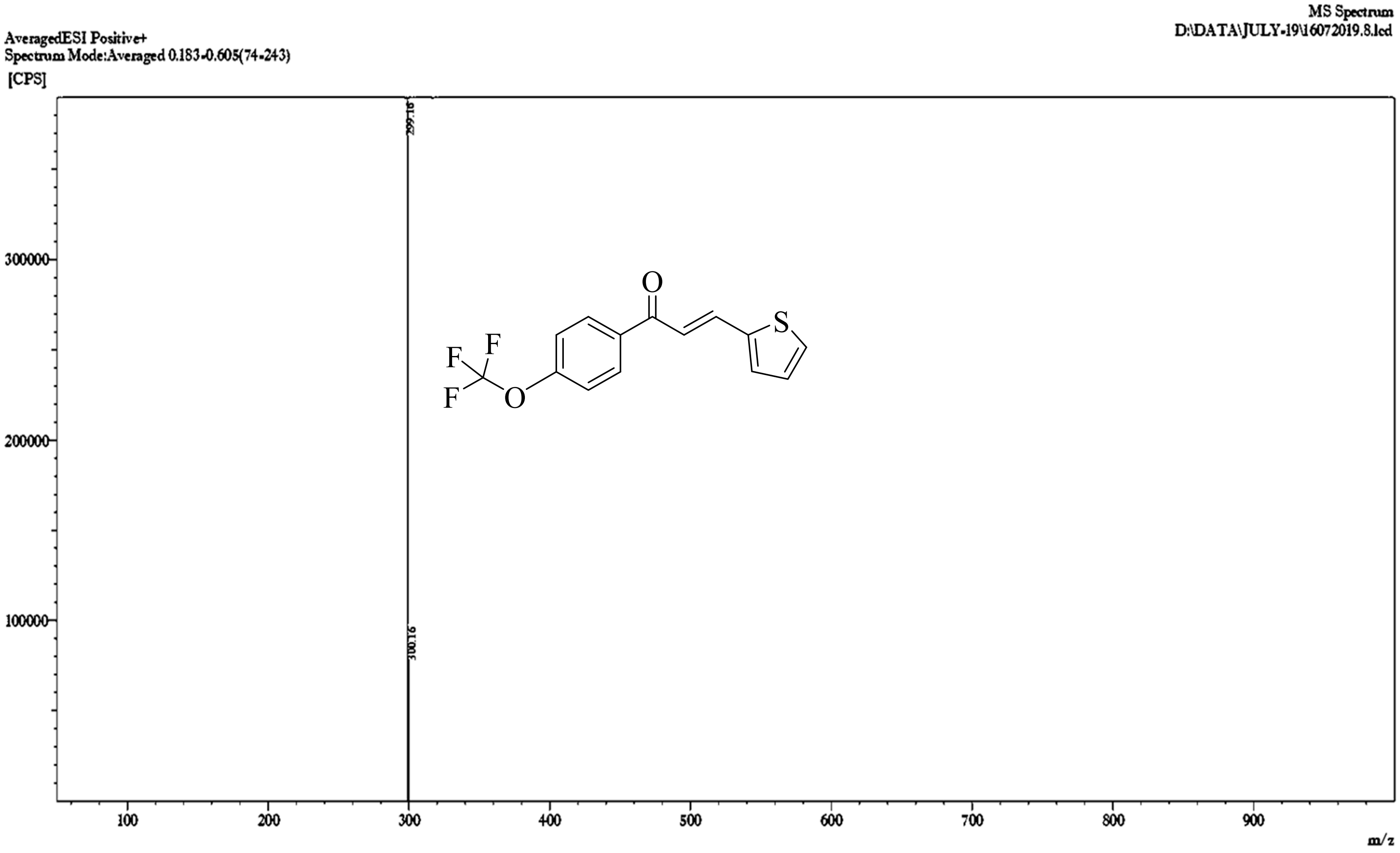

MASS Spectrum of (E)- 3-(thiophen-2''-yl)-1-[4'-(trifluoromethoxy)phenyl]prop-2-en-1-one (B6)

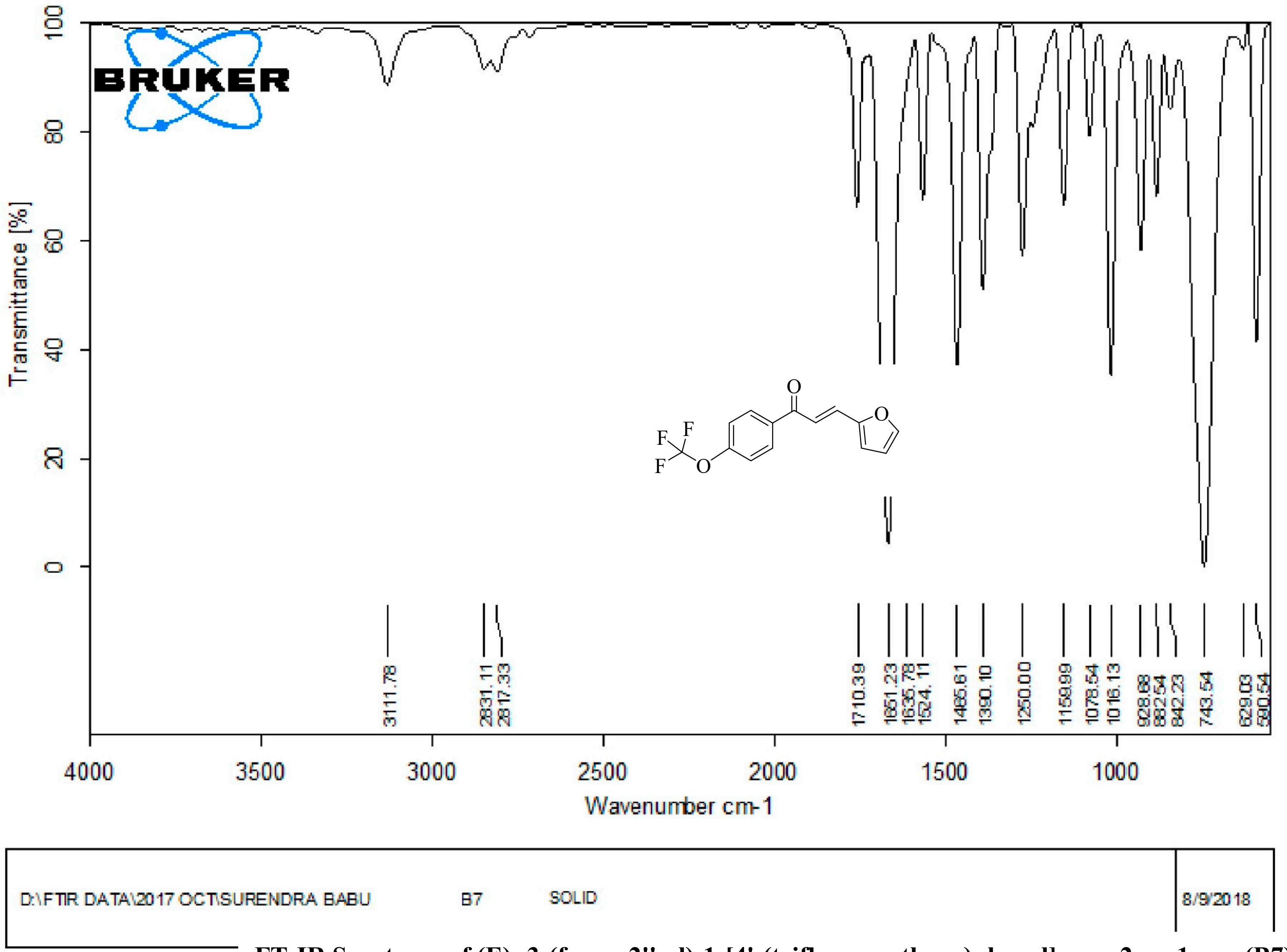

FT-IR Spectrum of (E)- 3-(furan-2''-yl)-1-[4'-(trifluoromethoxy)phenyl]prop-2-en-1-one (B7)

B7

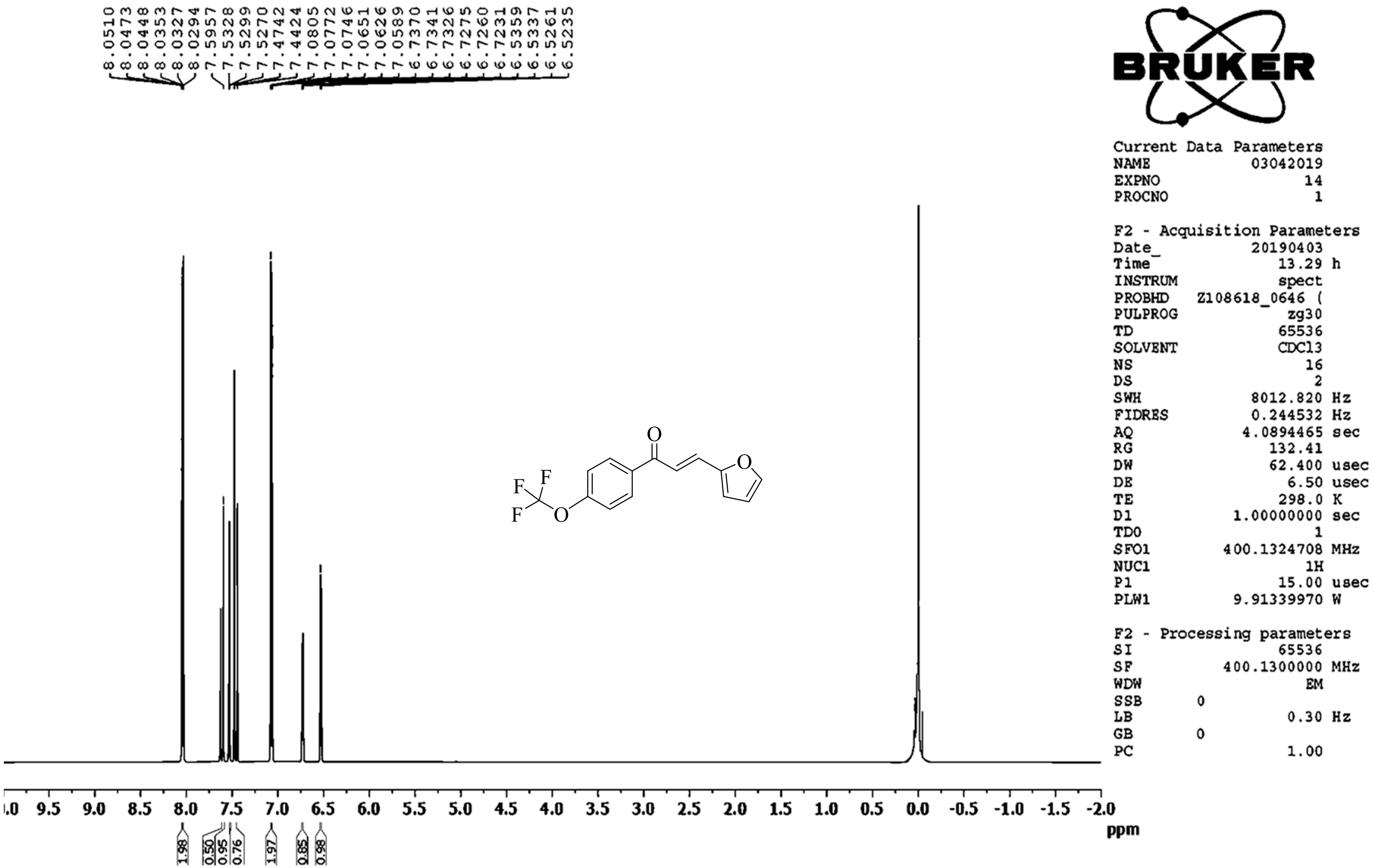

^{1}H NMR Spectrum of (E)- 3-(furan-2''-yl)-1-[4'-(trifluoromethoxy)phenyl]prop-2-en-1-one (B7)

Sample Name : **KSB-B7-282**
Data File : **16072019.9.lcd**
Date Acquired : **7/16/2019 10:11:00 AM**
Batch File : **16072019.lcb**

MS Spectrum
D:\DATA\JULY-19\16072019.9.lcd

AveragedESI Positive+
Spectrum Mode:Averaged 0.183-0.605(74-243)

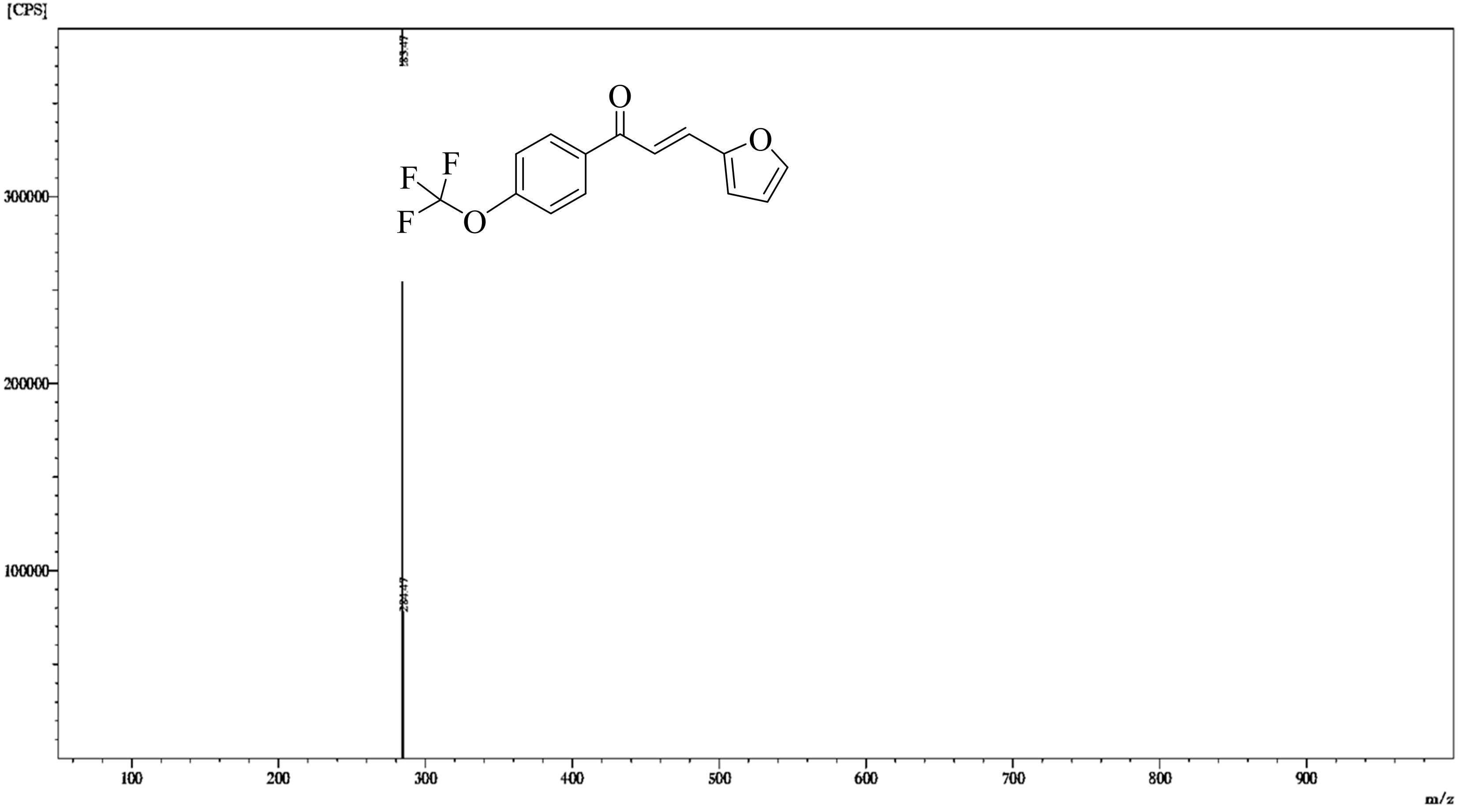

MASS Spectrum of (E)- 3-(furan-2''-yl)-1-[4'-(trifluoromethoxy)phenyl]prop-2-en-1-one (B7)

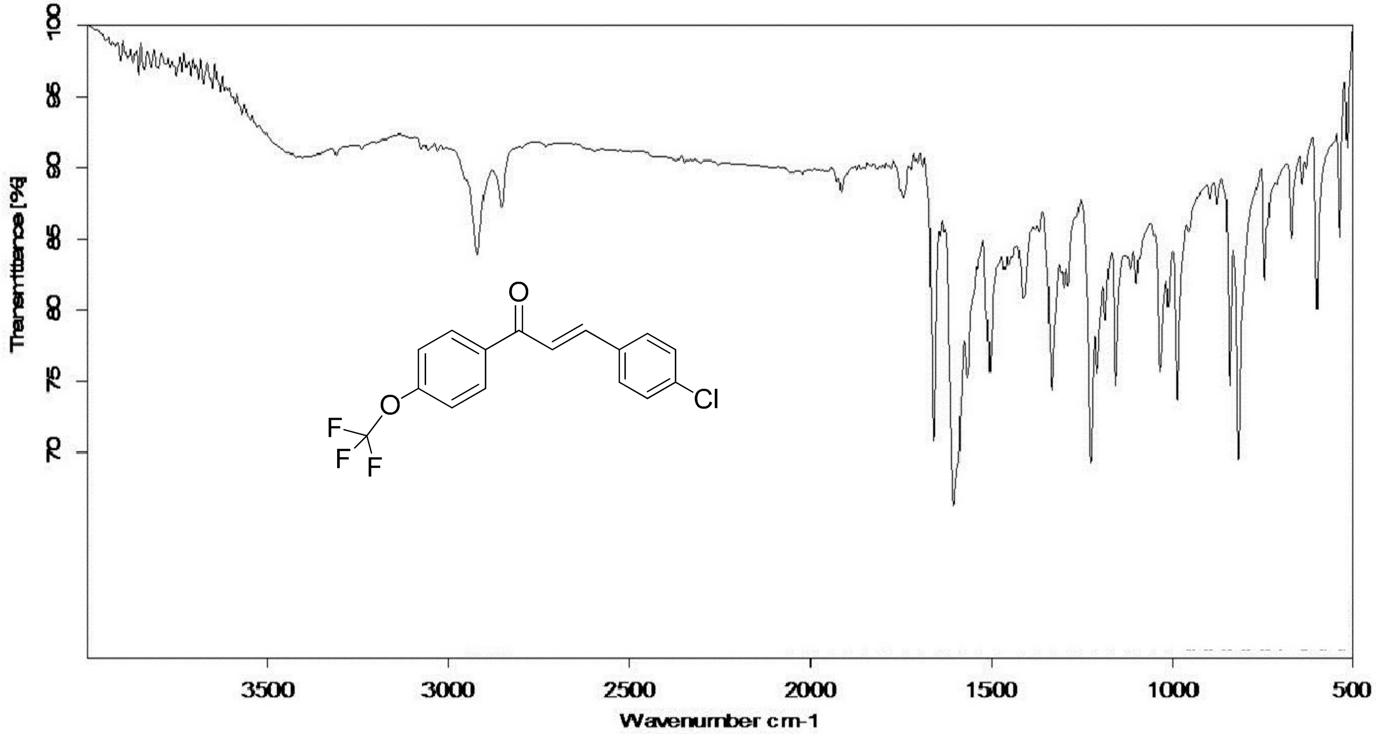

FT-IR Spectrum of (E)-3-(4''-chlorophenyl)-1-[4'-(trifluoromethoxy)phenyl]prop-2-en-1-one (B8)

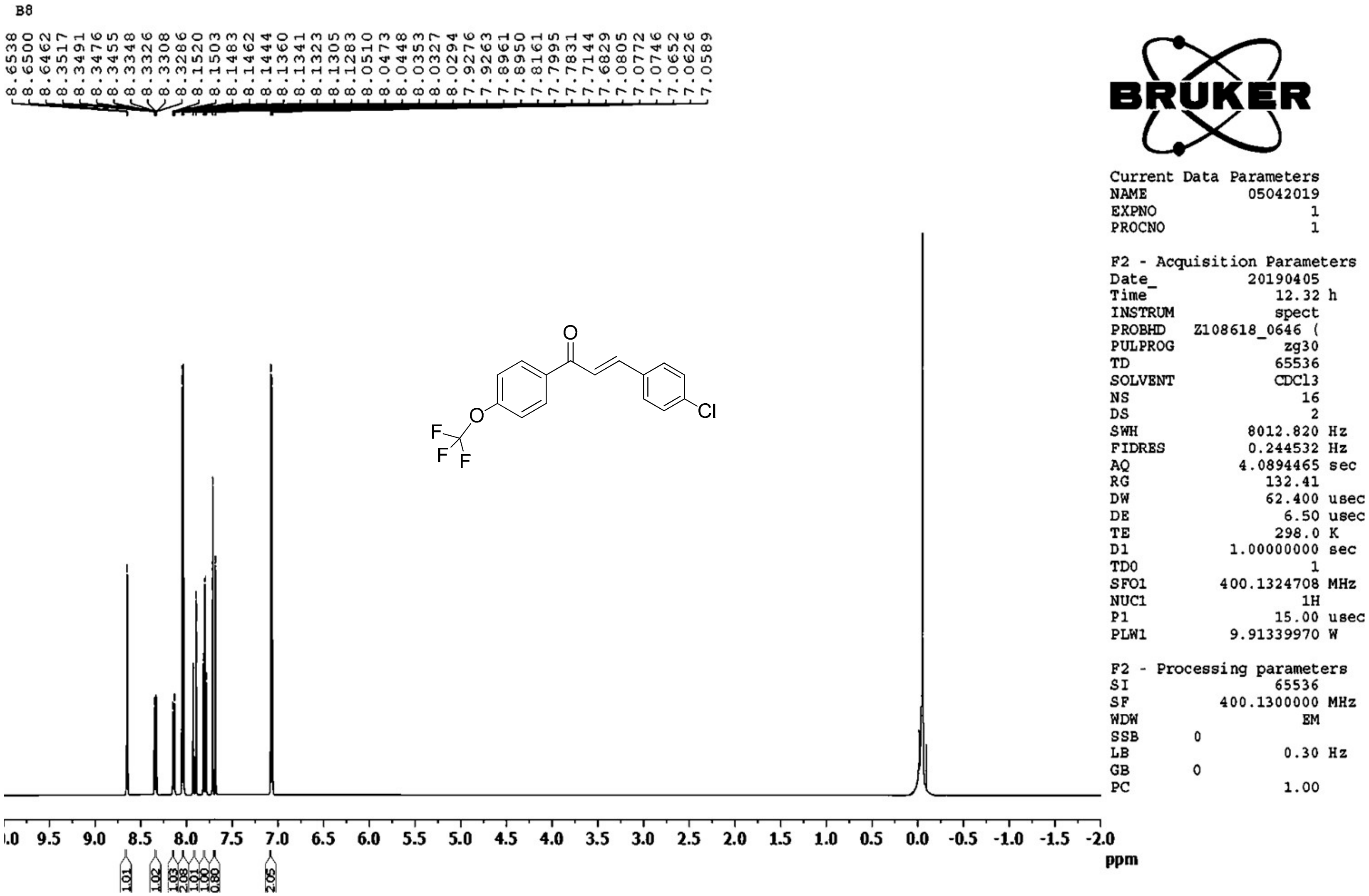

1H NMR Spectrum of (E)-3-(4''-chlorophenyl)-1-[4'-(trifluoromethoxy)phenyl]prop-2-en-1-one (B8)

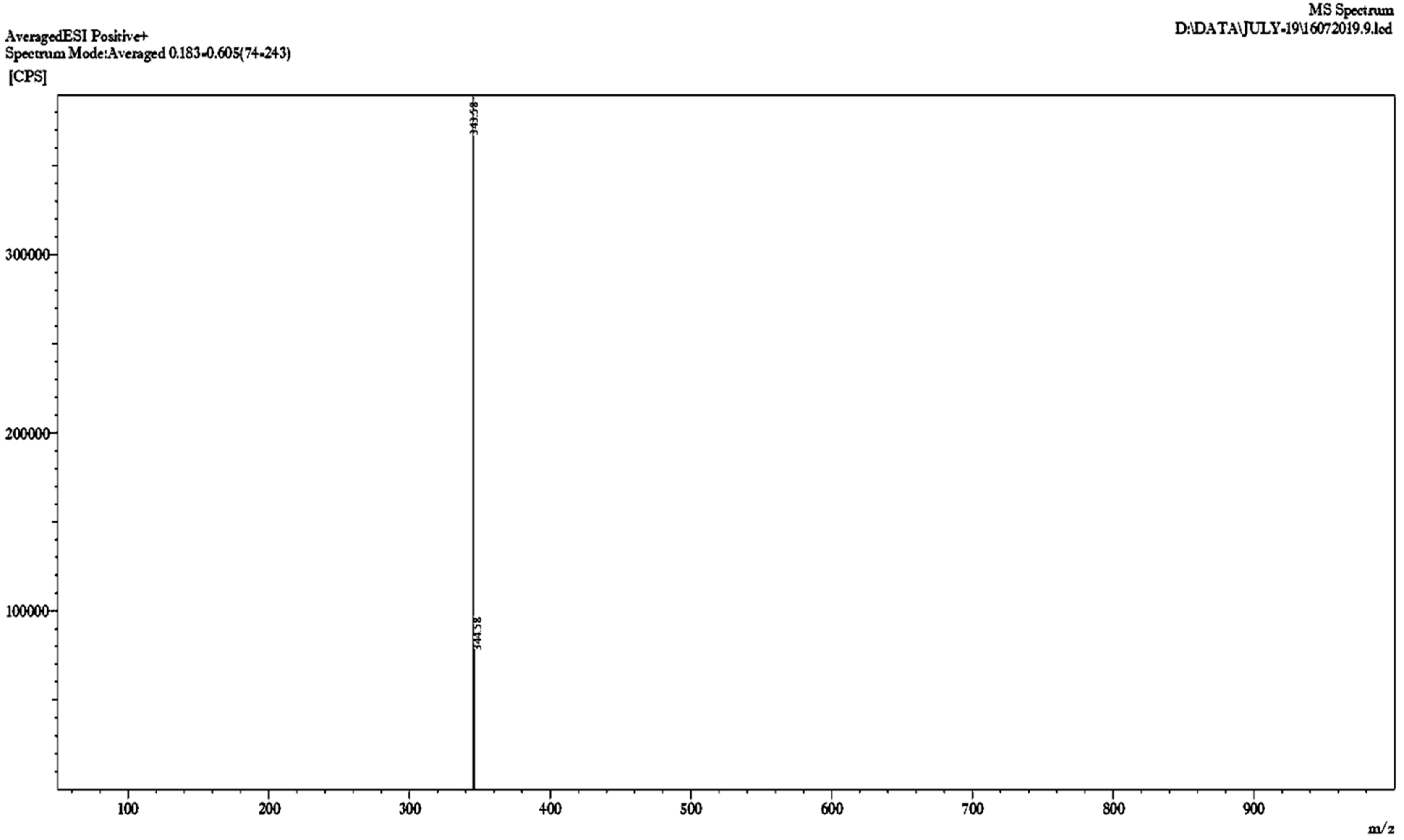

Mass Spectrum of (E)-3-(4''-chlorophenyl)-1-[4'-(trifluoromethoxy)phenyl]prop-2-en-1-one (B8)

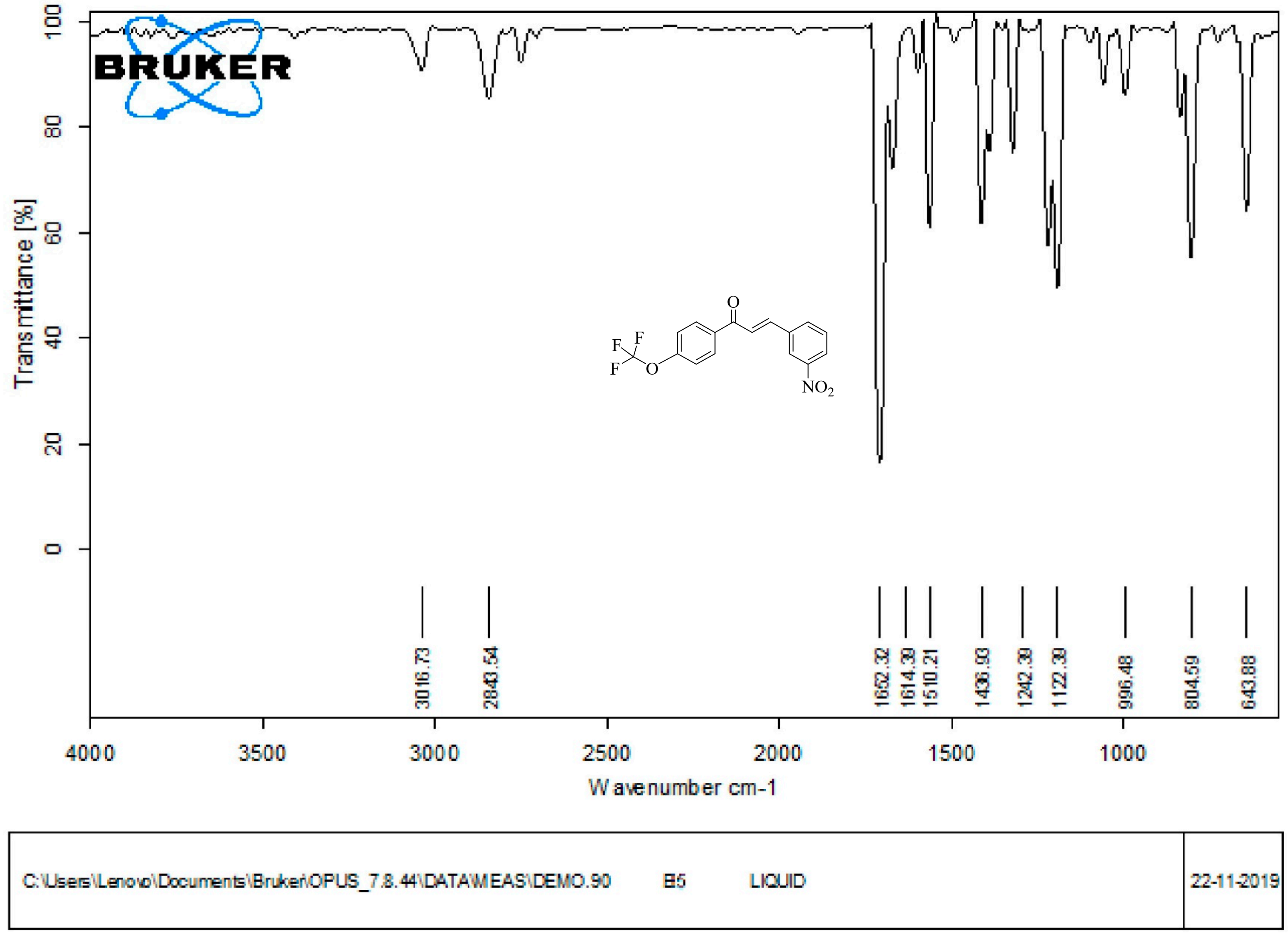

FT-IR Spectrum of (E)-1-(4-(trifluoromethoxy)phenyl)-3-(4-nitrophenyl)prop-2-en-1-one (B9)

B9

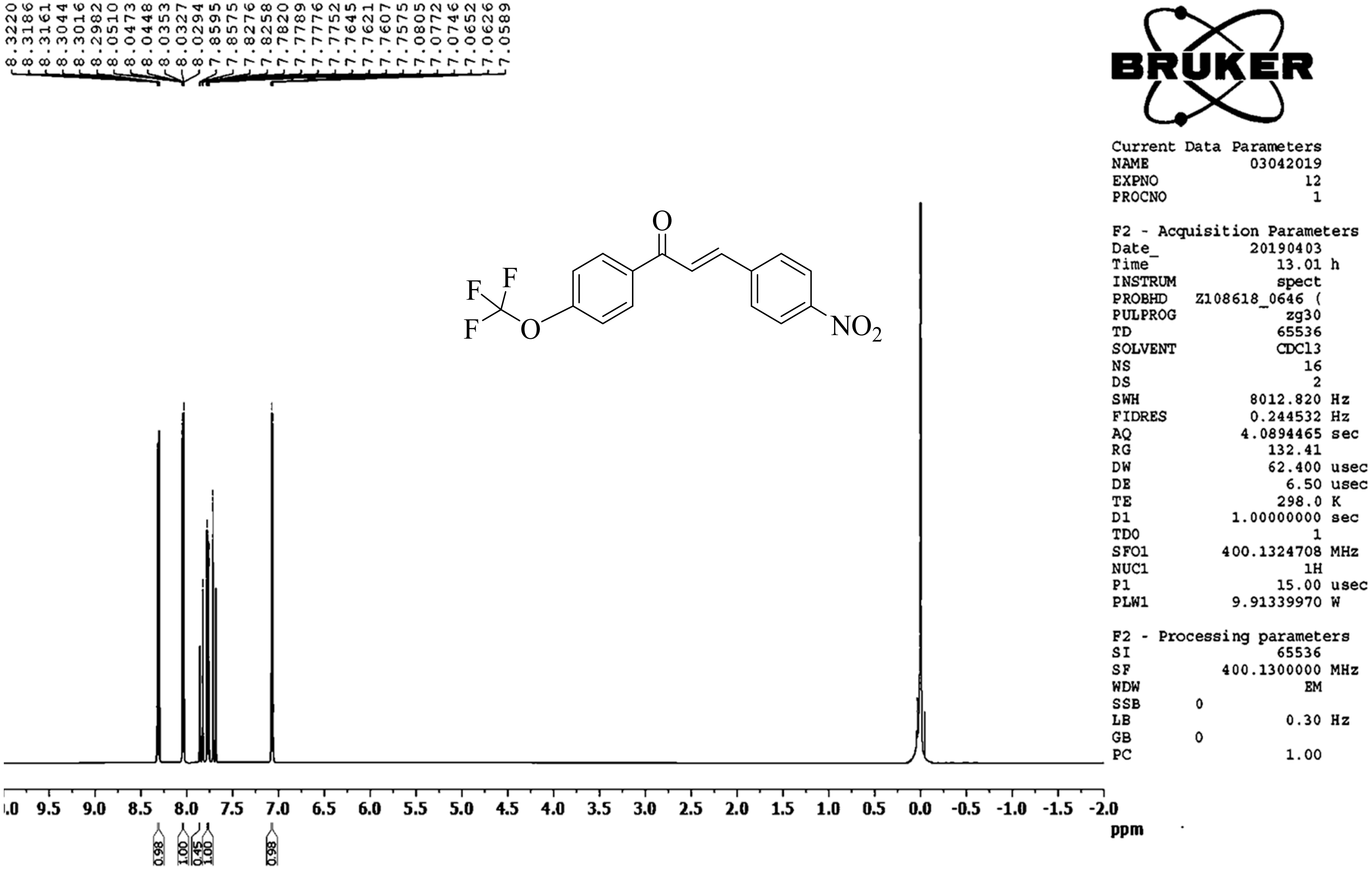

^{1}H NMR Spectrum of (E)- 1-(4-(trifluoromethoxy)phenyl)-3-(4-nitrophenyl)prop-2-en-1-one (B9)

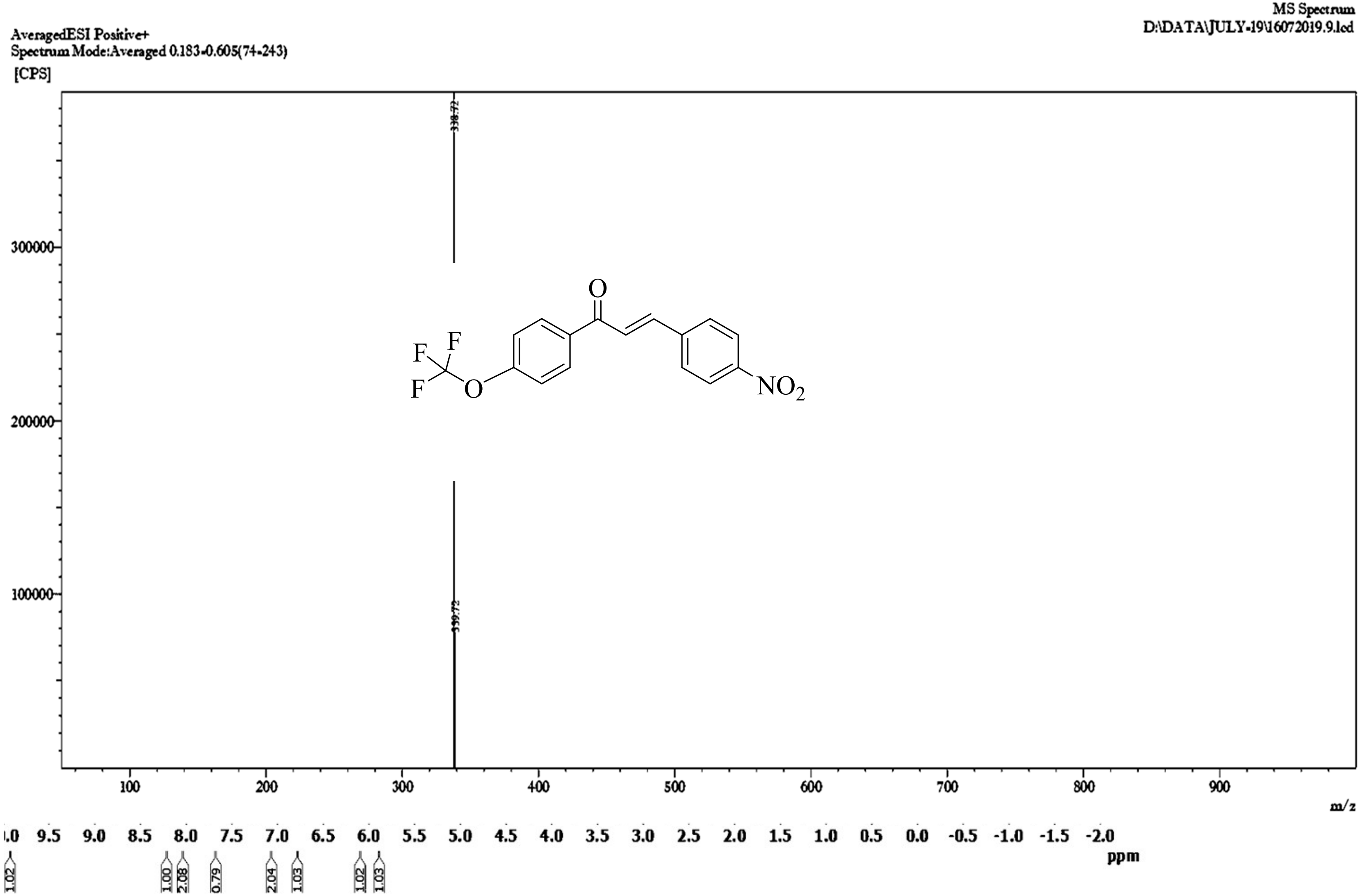

MASS Spectrum of 1-(4-(trifluoromethoxy)phenyl)-3-(4-nitrophenyl)prop-2-en-1-one (B9)

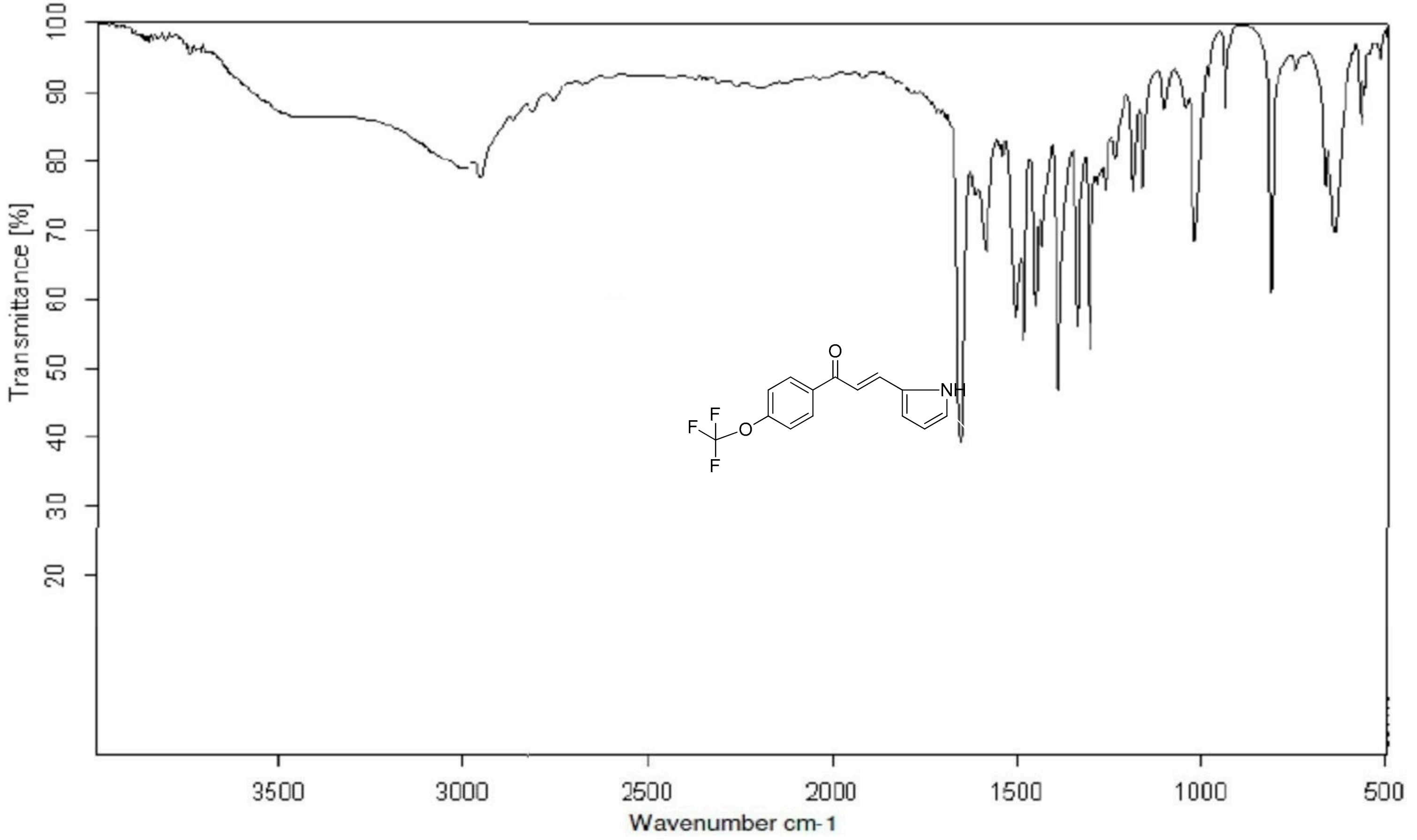

FT-IR spectrum of (E)-3-(1H-pyrrol-2-yl)-1-(4-(trifluoromethoxy)phenyl)prop-2-en-1-one (B10)

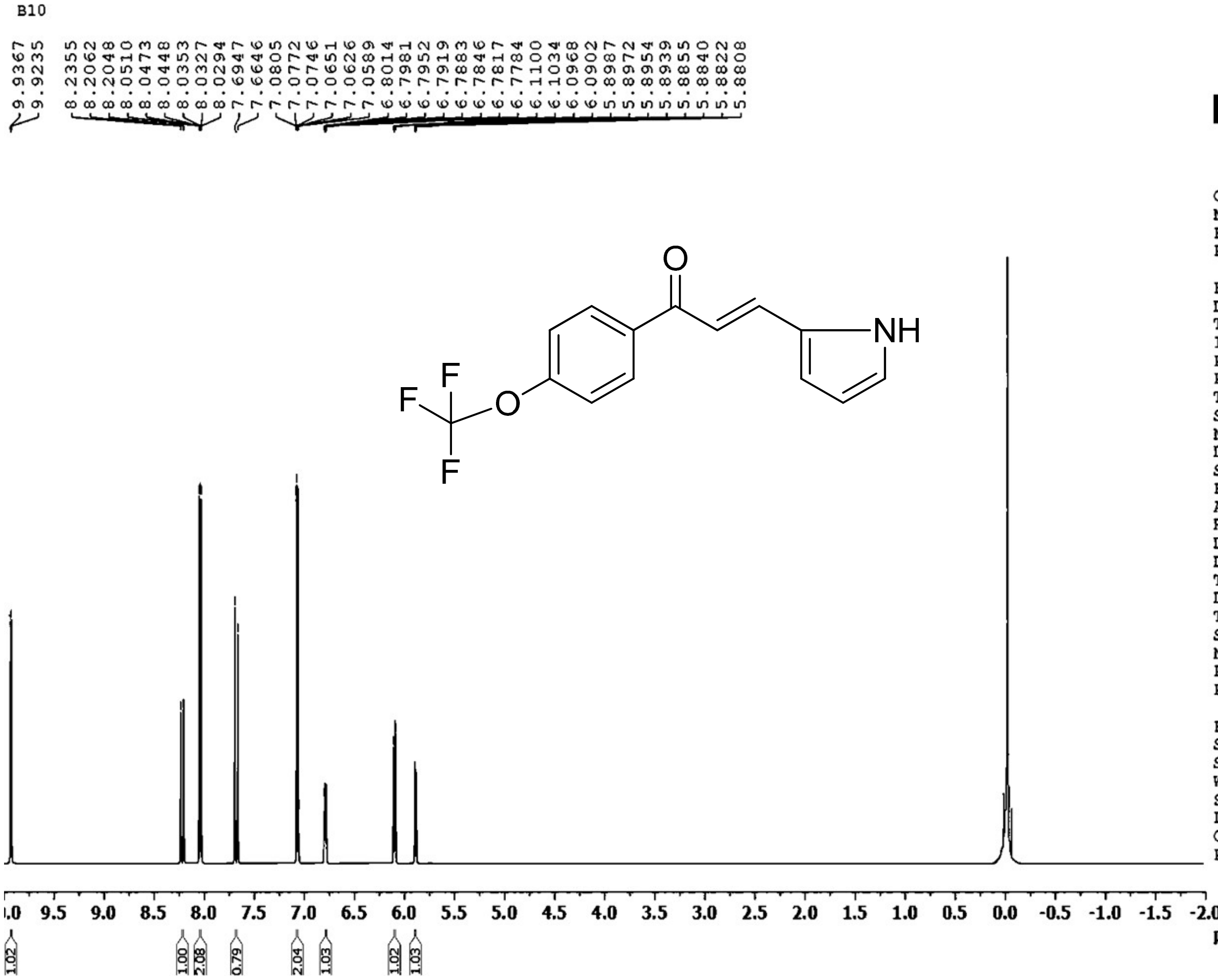

Current Data Parameters

NAME	05042019
EXPNO	1
PROCNO	1

F2 - Acquisition Parameters

Date_	20190405
Time	12.24 h
INSTRUM	spect
PROBHD	Z108618_0646 (
PULPROG	zg30
TD	65536
SOLVENT	CDCl3
NS	16
DS	2
SWH	8012.820 Hz
FIDRES	0.244532 Hz
AQ	4.0894465 sec
RG	132.41
DW	62.400 usec
DE	6.50 usec
TE	298.0 K
D1	1.00000000 sec
TD0	1
SFO1	400.1324708 MHz
NUC1	1H
P1	15.00 usec
PLW1	9.91339970 W

F2 - Processing parameters

SI	65536
SF	400.1300000 MHz
WDW	EM
SSB	0
LB	0.30 Hz
GB	0
PC	1.00

1H NMR spectrum of (E)-3-(1H-pyrrol-2-yl)-1-(4-(trifluoromethoxy)phenyl)prop-2-en-1-one (B10)

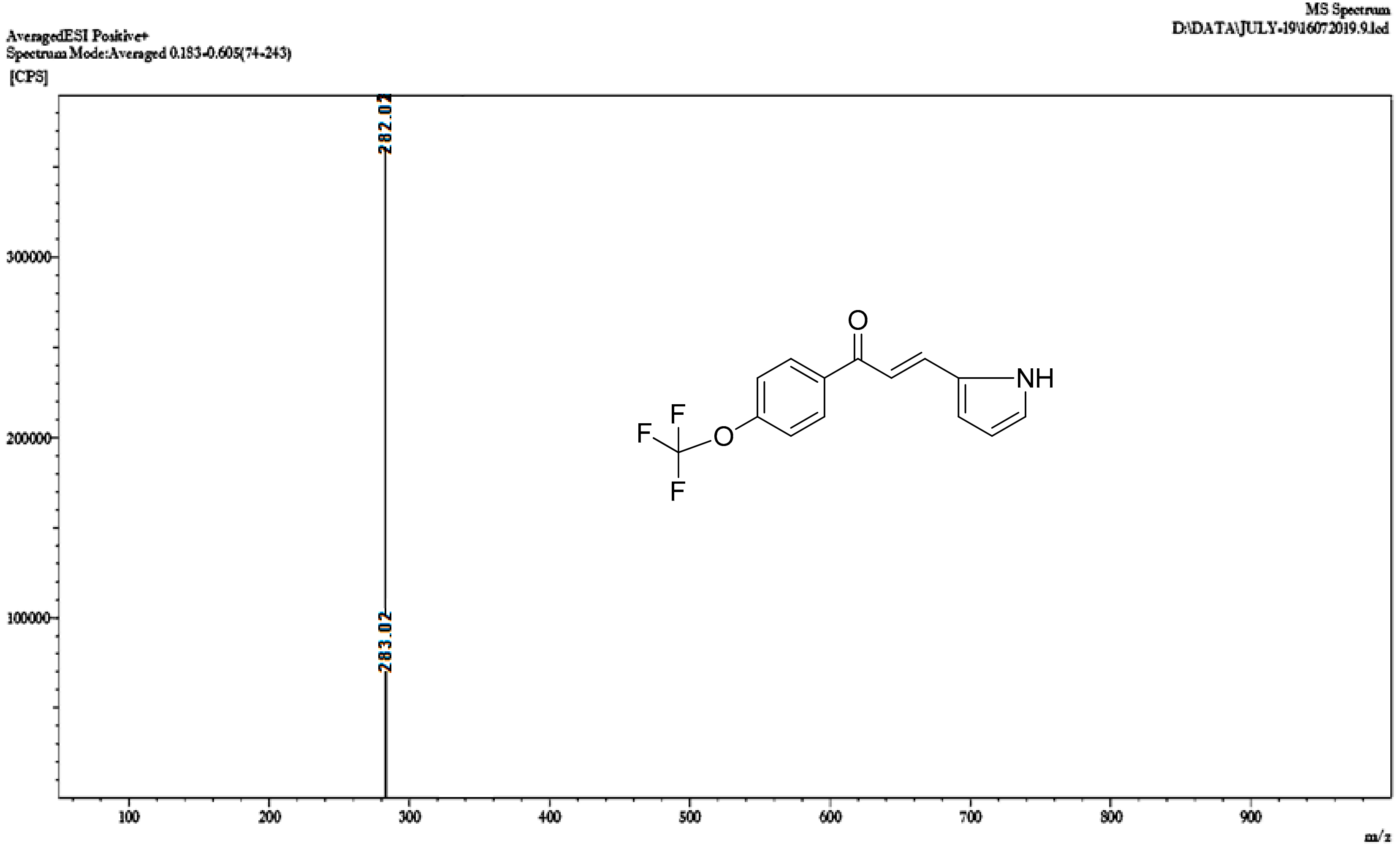

Mass spectrum of (E)-3-(1H-pyrrol-2-yl)-1-(4-(trifluoromethoxy)phenyl)prop-2-en-1-one (B10)
